# Evaluation of toxicity of aerosols from flavored e-liquids in Sprague–Dawley rats in a 90-day OECD inhalation study, complemented by transcriptomics analysis

**DOI:** 10.1007/s00204-020-02759-6

**Published:** 2020-05-05

**Authors:** Jenny Ho, Davide Sciuscio, Ulrike Kogel, Bjoern Titz, Patrice Leroy, Gregory Vuillaume, Marja Talikka, Elyette Martin, Pavel Pospisil, Stefan Lebrun, Wenhao Xia, Tom Lee, Yun Xuan Chng, Blaine W. Phillips, Emilija Veljkovic, Emmanuel Guedj, Yang Xiang, Nikolai V. Ivanov, Manuel C. Peitsch, Julia Hoeng, Patrick Vanscheeuwijck

**Affiliations:** 1 PMI S&I, Philip Morris International Research Laboratories Pte. Ltd., Science Park II, Singapore, Singapore; 2 PMI S&I, Philip Morris Products S.A., Quai Jeanrenaud 5, CH-2000 Neuchâtel, Switzerland

**Keywords:** E-liquid, Flavor, Inhalation toxicology, OECD, System toxicology

## Abstract

The use of flavoring substances is an important element in the development of reduced-risk products for adult smokers to increase product acceptance and encourage switching from cigarettes. In a first step towards characterizing the sub-chronic inhalation toxicity of neat flavoring substances, a study was conducted using a mixture of the substances in a base solution of e-liquid, where the standard toxicological endpoints of the nebulized aerosols were supplemented with transcriptomics analysis. The flavor mixture was produced by grouping 178 flavors into 26 distinct chemical groups based on structural similarities and potential metabolic and biological effects. Flavoring substances predicted to show the highest toxicological effect from each group were selected as the flavor group representatives (FGR). Following Organization for Economic Cooperation and Development Testing Guideline 413, rats were exposed to three concentrations of the FGR mixture in an e-liquid composed of nicotine (23 µg/L), propylene glycol (1520 µg/L), and vegetable glycerin (1890 µg/L), while non-flavored and no-nicotine mixtures were included as references to identify potential additive or synergistic effects between nicotine and the flavoring substances. The results indicated that the inhalation of an e-liquid containing the mixture of FGRs caused very minimal local and systemic toxic effects. In particular, there were no remarkable clinical (in-life) observations in flavored e-liquid-exposed rats. The biological effects related to exposure to the mixture of neat FGRs were limited and mainly nicotine-mediated, including changes in hematological and blood chemistry parameters and organ weight. These results indicate no significant additive biological changes following inhalation exposure to the nebulized FGR mixture above the nicotine effects measured in this sub-chronic inhalation study. In a subsequent study, e-liquids with FGR mixtures will be aerosolized by thermal treatment and assessed for toxicity.

**Electronic supplementary material:**

The online version of this article (10.1007/s00204-020-02759-6) contains supplementary material, which is available to authorized users.

## Introduction

For many decades, the foundational strategy for reducing smoking-related harm has been focused on preventing smoking initiation and promoting smoking cessation (Golechha 2016). In fact, the best way to reduce smoking-related harm is to quit tobacco use altogether. For adult smokers who would otherwise continue to smoke, switching to reduced-risk products^1^ (RRP) that emit significantly lower levels of harmful and potentially harmful constituents compared with those from cigarette smoke, may reduce the harm. However, it is important that adult smokers find these alternatives satisfying compared to smoking cigarettes. Therefore, the use of flavoring substances is an important factor in the development of RRPs to maximize product acceptance by adult smokers and encourage them to switch away from cigarettes. There is a large number of flavoring substances that can be potentially used in RRPs, and most of them are generally recognized as safe (GRAS) for use in food products (Hallagan 2016; Smith et al. 2003); however, there are limited data available on the safety of these flavoring substances in the context of inhalation (Helen et al. 2017; Kaur et al. 2018; Werley et al. 2016). There is a data gap in the efforts to understand the potential impact of flavoring substances on health through inhalation exposure.

Another concern about adding flavoring substances to tobacco products or RRPs is centered on the fact that substances may potentiate the toxicity of cigarette smoke or aerosol by producing new toxic constituents (due to pyrolysis or reaction), affecting new target organs, adding carcinogenicity, or acting in a synergistic manner to increase the toxicity. Testing programs or screening have been performed to evaluate the potential effects of tobacco ingredients—including flavoring substances added to typical commercial blended test cigarettes (Baker et al. 2004a, 2004b; Carmines 2002; Coggins et al. 2011; Paschke et al. 2002; Roemer et al. 2002; Rustemeier et al. 2002; Vanscheeuwijck et al. 2002) or to e-liquids (Costigan and Meredith 2015; Marescotti et al. 2019)—on smoke or aerosol chemistry and toxicity. Often, this involved complex chemical composition analysis and in vitro assessment (Famele et al. 2015; Iskandar et al. 2016; Lisko et al. 2015). The classical approach to evaluate the safety of a flavoring substance for inhalation would require a series of in vivo studies on individual substances. However, in light of the large number of flavoring substances that can potentially be used in RRPs, such an approach would be very laborious and would require many experimental animals. Therefore, we used a novel, combinational flavor group-based approach using standard and system toxicological methods to acquire information on the safety of flavoring substances via inhalation. Under this approach, a list of flavoring substances that can potentially be used in RRPs has been defined and subsequently clustered to pre-defined chemical groups of structurally related substances that are expected to exhibit similar metabolic and biological effects according to the European Commission Regulation (EC) No. 1565/2000 (European-Commission 2000). Using a combination of predictive software and extensive literature review, toxicological data for the flavoring substances in the list were collected. Following this, at least one flavoring substance predicted to possess the worst toxicological profile was selected from each chemical group as the flavor group representative (FGR). The concentration of each FGR was chosen to be similar to or higher than the concentration used in commercial formulations. Finally, a mixture of FGRs was generated for testing.

Propylene glycol (PG) and vegetable glycerin (VG) are humectants that moisten tobacco and are commonly used as vehicles and aerosol formation agents in e-cigarette formulations (i.e., e-liquids), while PG also serves as a solvent for flavors. The inhalation toxicity of a nebulized base solution for an e-liquid (PG and VG with nicotine) was previously assessed in a 90-day rat study and demonstrated that the base liquid had limited toxicity, which merely linked to the known effects of nicotine (Phillips et al. 2017). The aim of the current study was to characterize the toxicity following repeated inhalation exposure to the nebulized aerosols of flavored e-liquids (at three different concentration levels of the FGR mixture) with target nicotine, PG, and VG concentrations of 23 µg/L, 1520 µg/L, and 1890 µg/L, respectively. This exposure study was conducted for 13 weeks in Sprague–Dawley rats and in accordance with the Organization for Economic Organization for Economic Cooperation and Development (OECD) Guidelines For Testing of Chemicals—Test No. 413: Sub-chronic Inhalation Toxicity: 90-day study (TG 413) (OECD 2009). The toxicity of the FGR mixture was assessed in a base liquid without thermal treatment to understand the toxicity of the neat flavorings via the inhalation route. A group of rats exposed to phosphate-buffered saline (PBS) was included as a vehicle control group, while rats exposed to non-flavored e-liquid and no-nicotine FGR mixture (medium concentration) in PG/VG were also included as reference groups to reveal potential additive or synergistic effects between nicotine and the FGR mixture. The reversibility of the findings was assessed in selected treatment groups after a 42-day post-exposure recovery period. This experimental design represents the first step in the comprehensive assessment of the potential intrinsic toxicological impact of the FGR mixture in an e-liquid to determine the biological outcomes of nebulization exposure, independent of thermal treatment for aerosol generation and/or diverse commercial aerosol generation devices. Besides the analyzed endpoints listed in the OECD TG 413 for sub-chronic inhalation studies, respiratory physiology, pulmonary inflammation markers, and biomarkers of exposure in urine and blood were also measured. In addition, measurements of global transcriptome changes in nasal epithelial, lung, and liver tissues were performed to get molecular insights on the effect of the flavoring substances or nicotine. In a subsequent step, the effect of the thermally generated aerosol will be investigated, where the potential reaction or pyrolysis products of the flavoring substances and/or base liquid will be unraveled. This first step in a stepwise approach will allow basic understanding of the effects of unchanged flavoring substances delivered to the lungs.

## Methods

### Flavor toolbox generation

Given the specific aim for which RRPs are conceived for potential harm reduction, flavoring substances should be subjected to appropriate quality and safety standards to ensure that they are safe in the context of their intended use. The flavoring substances used are GRAS; however, the GRAS status is not sufficient to assess their potential toxicological impact on human health when administered via inhalation. To minimize risks from potential contaminants, only synthetic, high-purity, food-grade (FG) flavoring substances were used in this study. In general, the designation of “FG” for flavoring substances is indicative of the fact that the substances are not carcinogenic, mutagenic, or reprotoxic (CMR). However, classification criteria can differ by geography and local regulatory frameworks, and several flavoring substances have been excluded from the selection based on historic use, but exceptions may exist. Therefore, any substances classified as group 1, 2A, or 2B carcinogens by the International Agency for Research on Cancer, as well as those classified as CMR by the European Union (EU) (EU-OSHA 2014), were explicitly excluded. Moreover, such substances should not be used in any e-vapor product. The final list of flavoring substances considered in this study consisted of 178 flavors, the categorization scheme of which reported here is called a ‘flavor toolbox’ (Supplemental Table 1).

### Flavor clustering

To cluster molecules and associate them to groups by structural similarities, the 178 flavoring substances considered in this study and 2761 flavoring substances from the European Food Safety Authority (regulated as the list of flavoring substances used or intended to be used on the EU market in regard to EC No 872/2012) were drawn, or their chemical structures were generated using their Chemical Abstract Services registry numbers to calculate chemical similarities (European-Commission 2012). The 178 flavoring substances were then assigned into 26 of the 34 groups defined by EC No 1565/2000 (European-Commission 2000), which are recognized by the Joint Expert Committee on Food Additives as not presenting safety concerns (Supplemental Table 2). Flavoring substances within a given chemical group were expected to exhibit similar metabolic and biological properties. It can be postulated that the toxicological properties of a substance that is the most toxic of the cluster—the representative of the group (FGR)—should be considered to represent those of all structurally related flavoring substances within the same group. Because many (62) of the 178 flavors were allocated to the flavor group 1 (see Supplemental Table 2), it was decided to further split group 1 into group 1A (acids) and 1B (esters), each having its own group representative.

### Toxicological and physico-chemical fingerprinting

Using the European Chemicals Agency (ECHA Cited: 2014) and ToxPlanet (ToxPlanet) databases, toxicological data for each of the 178 flavoring substances in the toolbox were collected. Whenever multiple values for the same toxicological endpoint (e.g., median lethal dose [LD_50_]) were available, the ECHA approach was applied to select the most relevant one (ECHA Cited: 2014). In particular, in the context of Registration, Evaluation, Authorisation and Restriction of Chemicals (REACH), evaluation of data quality includes the assessment of the adequacy of the information for hazard/risk assessment and classification and labeling purposes in addition to relevance and reliability (REACH Cited: 2014). ECHA data categorization was used to only select data from reliable “key studies”.^2^ However, the available information for most flavoring substances was sparse and specific to the route of exposure (i.e., oral, only LD_50_ available). Therefore, the data from the published studies were supplemented with in silico toxicology predictions using the commercially available Toxicity Prediction by Komputer-Assisted Technology software (TOPKAT), which employs robust and cross-validated quantitative structure toxicity relationship (QSTR) models for assessing various measures of toxicity (BIOVIA Cited: 2014). The software was used to predict acute inhalation toxicity (median lethal concentration [LC_50_]), ocular irritancy, rodent carcinogenicity, developmental toxicity, and chronic toxicity. Furthermore, physico-chemical properties, such as volatility and solubility, were extracted for each flavor from SciFinder (SciFinder Cited: 2014) (calculated using ACD/Labs Software V11.02) with the purpose to exclude FGRs that would be insoluble in the e-liquid or too volatile for the inhalation experiment. Finally, Cramer classes (assigned using OECD QSAR Toolbox software (OECD Cited: 2014)) were used to estimate the threshold of toxicological concern (TTC) for each flavoring substance based on its chemical structure. The TTC concept and approach have recently been recommended by several regulatory and advisory authorities for chemical and contaminant risk assessment, and the application of inhalation TTC can be useful for substances with limited toxicological data needed for a quantitative inhalation risk assessment (EFSA-Scientific-Committee 2012; Nielsen and Larsen 2011).

### Selection of FGRs

The selection of the FGR was achieved using a scoring approach. Overall scores of 2, 1, and 0 were attributed to each flavoring substance according to its toxicological endpoint categories (lowest, second-lowest, and others), true or false values, Cramer classes, and compound volatility (Table 1).

**Table 1:** Scoring system

Score	LD_50_	Cramer class	Predicted LC_50_	Predicted ocular irritancy^a^	Weight of evidence rodent carcinogenicity^b^	Predicted chronic lowest observed adverse-effect level (LOAEL)	Predicted developmental toxicity^c^	Volatility
2	Lowest	Class III	Lowest	–	–	Lowest	–	Highest
1	Second lowest	Class II	Second lowest	True	True	Second lowest	True	Second highest
0	Others; > 5000 mg/kg	Class I	Others	False	False	Others	False	Others

^a^True means that it is an irritant

^b^True means that it is a carcinogen

^c^True means that it is toxic

Oral toxicity data do not provide information on potential effects on the respiratory tract, but they can provide valuable information with regard to potential systemic toxicity of the flavoring substances. Therefore, flavoring substances with an LD_50_ higher than 5000 mg/kg were considered of low concern (LD_50_ initial filtering) and excluded from further comparisons. Thus, within each group, there is one compound with score 2 (lowest LD_50_ in the group), one with score 1 (second-lowest LD_50_ in the group), and the rest were given score 0. Similarly, score 2, 1, or 0 was given for other toxicological endpoints (i.e., predicted LC_50_, predicted ocular irritancy, predicted rodent carcinogenicity, predicted chronic LOAEL, and predicted developmental toxicity) and volatility. Finally, Cramer classification was used to support the selection of FGR (Cramer et al. 1976). Cramer classes were used to define TTC for inhalation toxicity, and several studies consistently reported that chemicals assigned to Cramer class I are less toxic than those assigned to Cramer class III at both local (respiratory) and systemic levels. In particular, the application of the Cramer classification tree to a data set of 92 sub-acute or sub-chronic rat inhalation studies resulted in TTC values of 1400 µg/person/day (class 1) and 470 µg/person/day (class 3) for local effects (based on human lung weight of 650 g) and of 980 µg/person/day (class 1) and 170 µg/person/day (class 3) for systemic effects (Carthew et al. 2009). Although these TTC values have recently been proposed as a suitable tool in toxicological risk assessment of flavoring substances in e-liquids (Costigan and Meredith 2015), Cramer classes were only applied here to rank flavors based on their potential to induce toxicity.

Finally, a cumulated score (addition) was used to identify the FGR (Table 1) for each pre-defined flavor group. Thus, overall score 0 was attributed to flavoring substances of the least concern. It should be noted that this empirical approach may have some limitations. Indeed, because experimental data are not available in many cases, this approach used predicted outcomes for the different endpoints. Therefore, the selection of the most toxic FGR is approximate, based on the model. However, the final aim was to create a representative hypothetical mixture (probable worst-case scenario) to test in this inhalation study (Table 2).

**Table 2:** FGR used in this study

FGR	Flavor group (EC No 1565/2000)
Butyric acid	1A
Ethyl acetate, ethyl formate	1B
Isobutyl alcohol	2
Allyl hexanoate	3
*D*,*l*-citronellol	4
2-Heptanone	5
Linalool	6
*l*-carvone ^a^, *l*-menthone	8
*gamma*-Valerolactone	9
3-Methyl-2,4-nonanedione	10
Ethyl maltol	12
Furaneol	13
Phenethyl alcohol	15
Eucalyptol	16
Eugenyl acetate	18
3-(Methylthio)propionaldehyde	20
4-(*p*-Hydroxyphenyl)butan-2-one	21
Cinnamyl alcohol	22
Methyl salicylate	23
2-Ethyl-3,5-dimethylpyrazine	24
Guaiacol	25
Methyl anthranilate	27
2-Acetylpyridine	28
2-Acetylthiazole	29
Methyl cyclopentenolone (Cyclotene)	30
*alpha*-Pinene	31

^a^l-carvone was selected from the flavor list as a representative for aerosol marker and biomarker of exposure

### Selection of concentration

A mixture of FGRs was generated and tested in vivo using both standard and system toxicological methods. Testing was done at three different FGR mixture concentrations. The lowest concentration of each FGR in the mixture was selected based on the potential use of this FGR in RRPs according to odor thresholds and sensorial properties. All FGRs at these concentrations were brought together in a mixture designated as low flavor level. For the high flavor-level mixture, the highest concentration of each FGR in the mixture was selected based on the solubility limit and technical feasibility for this FGR, and brought together. Finally, the medium flavor-level mixture was assembled using FGR concentrations at an intermediate point between the low and high concentrations (which, for most flavoring substances, was at two-thirds of the highest level).

### Animals and treatment

Six-week-old outbred male and nulliparous, non-pregnant female Sprague–Dawley rats, bred under specific-pathogen-free conditions, were obtained from Charles River Laboratories (breeding area Raleigh R04, Raleigh, NC, USA). The health status of rats was verified by examining the health report from the sourced breeding area. Additional health checks, including comprehensive microbiological, serological and histopathological evaluation, were performed. The rats were housed and exposed in animal rooms with restricted access and under controlled conditions of good hygiene. Details on animal husbandry are provided in the Supplemental Material. The rats were individually identified by means of subcutaneously implanted transponders and were randomly (stratified by sex and body weight) allocated to the treatment groups (Table 3). Following the allocation, the relative standard deviation (RSD) of mean body weight for each group was ≤ 10% (maximum RSD of 8.0% for males and 5.1% for females). For the measurement of endpoints listed in the OECD guideline, 10 male and 10 female rats were allocated to each group, the “OECD” groups. In addition, 8 male and 8 female rats were added to selected groups to assess the reversibility of the toxicity. To characterize exposure-related effects at the transcriptome level, 8 male and 8 female rats were allocated to each group, the “OECD Plus” groups.

**Table 3:** Allocation of rats to exposure groups

Group		Nicotine (µg/L)	Flavor level	OECD		OECD Plus	
				Number of male rats	Number of female rats	Number of male rats	Number of female rats
PBS		NA	NA	10	10	0	0
PG/VG + Nic		23	NA	10	10	8	8
PG/VG + F-Med		NA	Medium	10	10	8	8
PG/VG + Nic + F-Low		23	Low	10	10	8	8
PG/VG + Nic + F-Med		23	Medium	10	10	8	8
PG/VG + Nic + F-High		23	High	10	10	8	8
PG/VG + Nic (R) ^a^		23	NA	8	8	0	0
PG/VG + Nic + F-High (R) ^a^		23	High	8	8	0	0
			Total	76	76	48	48

^a^ (R) Post-exposure (“recovery”) groups

The nose-only exposure method was used in this study, which ensured daily reproducibility of aerosol uptake while minimizing aerosol deposition on the animal fur to prevent subsequent uptake by grooming. The rats were individually exposed to nebulized aerosols in the respective chambers. The test groups were exposed to 3 different concentrations of FGR mixture in e-liquids and compared with vehicle control group (PBS) or reference groups (i.e., non-flavored or no-nicotine) to assess the general toxicity of flavoring substances and nicotine on multiple biological endpoints following inhalation exposure. The exposure concentrations were controlled based on the base solution of the e-liquid, namely nicotine, PG, and VG levels in the test atmospheres, which were targeted at 23 µg/L, 1520 µg/L, and 1890 µg/L, respectively, in all groups (except the vehicle control group). Prior to the 90-day exposure, a 5-day tolerance test was performed on separate group of rats to confirm that the rats would tolerate the highest concentration of flavored e-liquid foreseen to be tested during the sub-chronic inhalation study, thereby minimizing the possibility of unexpected adverse effects during the study (not shown).

This study was conducted in compliance with the OECD Principles of Good Laboratory Practice (GLP) (OECD 1998), with the exception of bronchoalveolar lavage fluid (BALF) analysis using Luminex bead-based multiplex assay, determination of selected exposure biomarkers (Analytisch-biologisches Forschungslabor GmbH (ABF), Munich, Germany), and the transcriptomic investigations. The test facility is National Parks Board/Animal and Veterinary Service (NParks/AVS)-licensed and Association for Assessment and Accreditation of Laboratory Animal Care (AAALAC)-accredited, and care and use of the rats were conducted in accordance with the National Advisory Committee for Laboratory Animal Research Guideline (NACLAR 2004) and AAALAC requirements (AAALAC 2011). All animal experiments were approved by the Philip Morris International Research Laboratories Institutional Animal Care and Use Committee.

### Aerosol generation and test system exposure

Aerosols were generated as previously described (Phillips et al. 2015; Phillips et al. 2017), using commercially available 6-jet Collison nebulizers (BGI, Waltham, MA) that function to nebulize liquid solutions through small apertures by applying compressed dry air at 19 L/min and 37.5 PSI, resulting in the production of fine aerosols. Stock solutions were stored in a controlled access fridge (2–8 °C) and used within 6 days of preparation. The composition of the various stock solutions for test and reference items are listed in Supplemental Table 3. All batches of the stock solution were found to be free of microbial contamination, and endotoxin levels (performed by an ISO 17,025-accredited laboratory) were < 1 EU/mL. Additionally, the concentrations of stock solution components (i.e., nicotine, PG, VG, and flavoring substances) were determined and verified.

The stock solutions were constantly mixed using a magnetic bar and magnetic stir plate before entering the nebulizer reservoir, and warm water (30 ± 2 °C) was circulated around the nebulizers to reduce the viscosity of the stock solution to generate aerosols of appropriate concentration and droplet size distributions. Each inhalation chamber required the output of two 6-jet nebulizers (except for the PBS chamber, which required only one) to be combined. The aerosol was subsequently diluted with filtered conditioned air immediately downstream of the nebulizers to achieve the desired composition of PG and VG in the flow-past inhalation chambers (FPC1-232, anodized aluminum, with volume inside the compartment of approximately 3 L). The mean temperature of supplied aerosols at the inlet to exposure chambers was maintained at 22 ± 3 °C.

The repeated-dose exposure was conducted for at least 13 weeks, for 6 h per day, 5 days per week. A 7-day time-adaptation phase was included in the first week, in which the rats were exposed for increasing exposure durations over 7 days (1.5 h for the first and second days, 3 h for the third and fourth days, and 4.5 h for days 5 to 7), and from day 8 onward for the planned 6-h exposure. Weekend exposures (7 days per week exposure) were performed prior to scheduled necropsy, as necessary, to ensure that all rats were subjected to a minimum of 2 consecutive exposure days before necropsy. A post-exposure recovery period of 42 days was included to investigate reversibility, persistence, and delayed occurrence of exposure effects in selected groups of rats.

### Analytical characterization of test atmospheres and biomonitoring

To characterize the test atmosphere and to check the reproducibility of aerosol generation, the concentrations of total particulate matter (TPM), nicotine, PG, VG, *l*-carvone (a representative of the FGR mixture), flow rate through the exposure chamber, conductivity (ion concentration of aerosol from PBS only), relative humidity (aerosol from PBS only), and droplet size distribution were sampled for or tested at the breathing zone of the exposure chambers (Supplemental Table 4). To monitor aerosol uptake by the rats, biomonitoring was performed through analysis of selected biomarkers of exposure in urine and blood. Further details of sample collection and analysis are available in the Supplemental Material.

### Biological parameters

Biological parameters such as body weight, food consumption, ophthalmic condition, respiratory physiology, and in-life observations were monitored throughout the study and determined as described elsewhere (Wong et al. 2016) (see Supplemental Table 5 for the methods and frequency of determination). Details on evaluation of biological parameters are also described in the Supplemental Material.

### Pathology

Gross pathological analysis and organ collection were performed in all OECD groups (Supplemental Table 7). The organs were fixed and histological sections were processed as described in the Supplemental Material. The histopathological evaluation was performed by Dr. Patricia Ferreira Neves and peer-reviewed by Dr. Vasanthi Mowat (Envigo CRS Limited, Huntingdon, UK). Morphometric analysis and evaluation of bone marrow smears were also performed as described in the Supplemental Material.

### Transcriptomics analysis ("Plus" part)

The transcriptomics analysis was performed on samples from OECD Plus groups (Table 3). To avoid overlapping dissection dates with OECD groups and to harmonize the dissection workflow, OECD Plus groups were dissected 1 week after the OECD dissection (i.e., study days 96–102), thereby slightly exceeding the set 90-day exposure period. Dissection and tissue collection for the OECD Plus groups were performed on non-fasted animals 16–24 h post-exposure. Further details on organ collection and sample and data analyses are available in the Supplemental Material. GLP compliance was not claimed for this specific endpoint.

### Statistical evaluation

For in-life observations and mortality/morbidity, ophthalmology, and gross pathology, only individual or group findings were listed. No summary statistics were computed. For the aerosol uptake (selected nicotine metabolites, biomarkers of exposure, and respiratory physiology) and other OECD toxicity endpoints, descriptive statistics were computed for each exposure group and sex and, when relevant, for various time points (e.g., body weight). In addition, pairwise differences were also calculated separately for each sex (Supplemental Table 6), as described in the Supplemental Material. For endpoints measured during the 90-day exposure period (e.g., body weight), recovery groups were merged with the group having received the same treatment for statistical analysis (e.g., PG/VG + Nic + F-High OECD rats and PG/VG + Nic + F-High OECD (R) rats were considered as belonging to the same and unique groups).

## Results

### Test atmosphere characterization and aerosol uptake

The use of Collison Nebulizers as a means of aerosol generation and exposure has been demonstrated previously as appropriate approach for studying exposure via inhalation (Phillips et al. 2015, 2017; Shao et al. 2012) that allowed for exact control of exposure conditions, including the reproducible exposure to different concentrations of flavored e-liquids. The concentrations of nicotine, TPM, PG, VG, and *l*-carvone in the exposure chambers were monitored daily, and the results (Table 4) demonstrated that the aerosols were reproducibly generated and delivered to the exposure chambers throughout the exposure period. The study mean nicotine, PG, and VG concentrations in the exposure chambers were within the target range (target ± 10%), while *L*-carvone (one of the 28 flavors, selected as a representative flavoring substance) levels showed stable and consistent delivery in the expected concentration-dependent manner when measured in the test atmosphere. The droplet size distribution was determined using a spectrophometric aerodynamic particle sizer at least once per week per exposure chamber during the study, and the resulting mass median aerodynamic diameter (MMAD) and geometric standard deviation (GSD) values were consistent throughout the exposure period and equally respirable among all groups (i.e., MMAD ranged from 1.2 to 2.0 µm; GSD ranged from 1.5 to 2.0). These ranges were within the recommended range of 1–3 µm (MMAD) and 1.5–3 (GSD) in OECD TG 413 (OECD 2009). In addition, the MMAD and GSD values were found to be consistent with previously published results using similar e-liquid vehicle (Phillips et al. 2017).

**Table 4:** Characterization of test atmospheres

Chamber	Droplet size distribution		Nicotine (µg/L)	PG (µg/L)	VG (µg/L)	*l*-carvone (µg/L)
	MMAD (µm)	GSD				
PBS	1.20–1.41 (15)	1.54–1.69 (15)	0.0 ± 0.0 (21)	NM	NM	NM
PG/VG + Nic	1.46–1.99 (15)	1.68–2.07 (15)	22.7 ± 1.7 (79)	1600 ± 153 (80)	2012 ± 148 (80)	0.0 ± 0.0 (19)
PG/VG + F-Med	1.46–2.02 (15)	1.79–1.96 (15)	0.6 ± 2.7 (23) ^a^	1656 ± 183 (80)	2057 ± 214 (80)	4.0 ± 0.8 (80)
PG/VG + Nic + F-Low	1.57–1.96 (15)	1.78–2.04 (15)	22.7 ± 1.9 (80)	1608 ± 127 (80)	2016 ± 163 (80)	1.4 ± 0.5 (80)
PG/VG + Nic + F-Med	1.57–1.95 (15)	1.84–1.99 (15)	22.9 ± 2.1 (80)	1658 ± 160 (80)	2063 ± 170 (80)	4.0 ± 0.9 (80)
PG/VG + Nic + F-High	1.57–1.94 (15)	1.81–1.97 (15)	22.3 ± 1.6 (80)	1645 ± 115 (80)	2057 ± 156 (80)	6.0 ± 1.3 (80)

Measurements were performed in the breathing zones of the exposure chambers

Results are presented as mean ± SD; droplet size distribution-related measurements (MMAD and GSD) are presented as range [min–max]

Abbreviations: MMAD, mass median aerodynamic diameter; GSD, geometric standard deviation; NM, not measured

The number of determinants or daily means is given in parentheses

The raw data with values < limit of detection (LOD) were replaced by LOD/2 at the point of statistical calculation to compute the study mean values, except the samples with technical errors

^a^All samples were < LOD, except one sample (due to technical error)

TPM concentrations were measured on a daily basis. However, a technical issue with static discharge encountered during the weighing, causing high variations, rendered the results inconclusive. As a result, the TPM data in this study were excluded for further evaluation. Nevertheless, the delivery of aerosol to the exposure chambers was sufficiently well verified by analytically determining the levels of other components (nicotine, PG, VG, and *l*-carvone) in the aerosol.

Respiratory physiology measurements were obtained once in the study (study days 29–40) to assess potential irritation in the upper respiratory tract or changes in respiratory behavior caused by the aerosols (Supplemental Fig. 1a–d). In general, no significant differences were observed in breathing parameters of male rats exposed to all aerosols when compared with PBS control, suggesting that the flavors had no irritating effects on the upper respiratory tract of the male rats. Female rats exposed to medium and high concentrations of flavored e-liquids showed lower breathing frequency (*p* ≤ 0.05) in comparison to females exposed to PBS control, but there were no significant flavor concentration-dependent changes, while the tidal volume was higher relative to PBS control and non-flavored exposed groups (*p* ≤ 0.05). Subtle effects of exposure on minute volume were observed mainly in females exposed to medium concentration of flavored e-liquid and in males exposed to non-flavored e-liquid aerosols (both *p* ≤ 0.05), likely due to the higher tidal volume seen in both exposure groups (*p* ≤ 0.01) (Supplemental Table 8).

The aerosol uptake by the rats was reflected in the concentrations of nicotine, cotinine, PG, and selected flavoring substances (*l*-carvone, linalool, and citronellol) in the blood (plasma) as well as the amounts of nicotine metabolites recovered in 24-h urine samples. The nicotine concentrations in plasma were similar across groups exposed to nicotine-containing aerosols (nicotine aerosol concentration 23 µg/L) (Supplemental Table 9 and Supplemental Fig. 2a, b), which showed similar uptake of nicotine for the relevant exposure groups. During analysis, it was noted that there were trace amounts of nicotine in the plasma from rats in the recovery groups. However, the mean values were lower than or close to the quantification limit of the analytical detection method (100.6 ng/mL) and mainly derived from a few rats from different groups, therefore, likely to be crossover contaminants from analytical procedures or background. Cotinine levels were low in the recovery groups, further proving that the rats were not exposed to nicotine during that period of time.

A similar result was observed for the nicotine and total of the five major urinary nicotine metabolites (cotinine, nicotine-N’-oxide, nornicotine, norcotinine, trans-3′-hydroxycotinine) in all groups (Supplemental Fig. 2C). The total amount of urinary nicotine metabolites excreted by the rats during the 24-h collection period was similar among males and females, with the exception of the PG/VG + Nic + F-Low group, where a statistically significantly higher recovery was noted (*p* ≤ 0.01) (Supplemental Table 9). Absolute amounts of the five nicotine metabolites were similar across the male groups, indicating similar nicotine metabolism in both male and female rats.

**Fig. 1:**
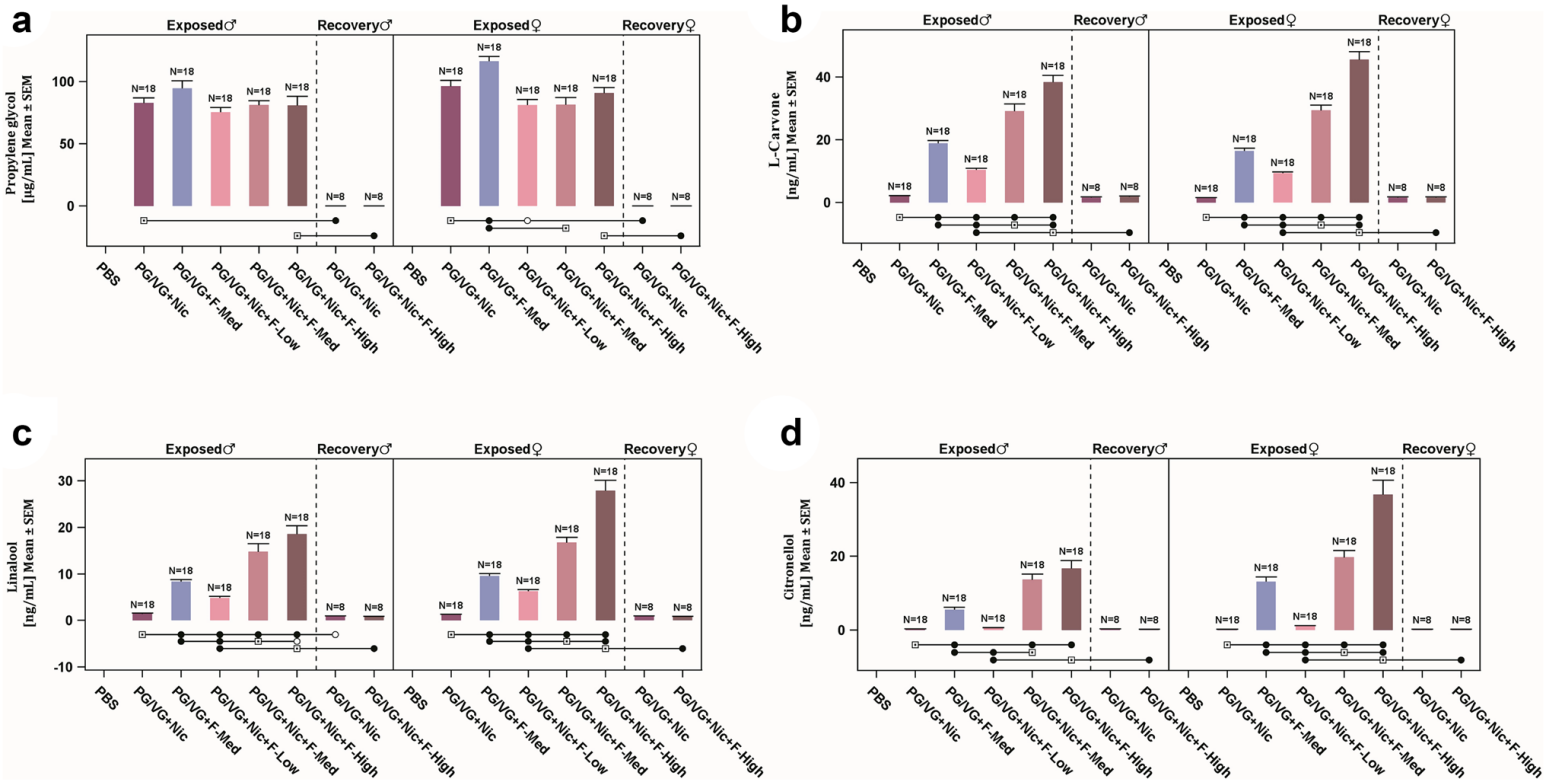
Plasma biomarkers of exposure. Results are presented as mean ± SEM for PG (**a**), *l*-carvone (**b**), linalool (**c**), and citronellol (**d**) concentrations in plasma for male and female rats. Statistically significant differences are represented by empty circles (*p* ≤ 0.05) or filled circles (*p* ≤ 0.01) when compared with the groups indicated by the squares underneath the bars

Four additional biomarkers of exposure, namely PG, *l*-carvone, linalool, and citronellol, were also quantified in the plasma samples (Analytisch-biologisches Forschungslabor GmbH, Munich, Germany). Plasma concentrations of PG were measured at levels expected based on test atmosphere concentration. The PG concentrations recovered in the plasma from the rats exposed to flavored e-liquids were lower than those from rats exposed to non-flavored or no-nicotine reference aerosols, independent of sex (Fig. 1a), despite the fact that the PG levels were matched in test atmosphere. Nonetheless, the plasma PG concentrations reported here, especially for the non-flavored reference (PG/VG + Nic)-exposed group, generally correspond to those determined in the previous sub-chronic inhalation study in rats with a similar e-liquid formulation (Phillips et al. 2017). Similarly, the analysis of the three additional biomarkers of exposure for flavoring substances (i.e., *l*-carvone, linalool, and citronellol) in plasma showed a concentration dependency in both sexes (Fig. 1b–d), thus further confirming the presence of the flavoring substances in the test atmospheres and their consistent delivery. However, it was noted that in the PG/VG + Nic + F-High chamber, the plasma concentration of the flavoring substances linalool, citronellol, and, to a certain extent, *l*-carvone for males were similar with the levels for males in the PG/VG + Nic + F-Med chamber, while in the females, the expected concentration dependency was observed. Because males and females were exposed in the same inhalation chamber, this variation cannot be due to the quality of the aerosol or the transfer rates from the solution to the aerosol. The observed variation in biomarkers of exposure between males and females in the PG/VG + Nic + F-High chamber may be due to gender differences in hepatic metabolism, which often results from differences in expression of hepatic enzymes (Czerniak 2001). In addition, the recovered flavor compound levels were lower in both sexes following exposure to the no-nicotine (PG/VG + F-Med) reference aerosol (*p* ≤ 0.05) when compared with flavored e-liquid at medium concentration (PG/VG + Nic + F-Med), which may be due to lower minute volume.

### In-life observations

The rats showed typical signs of stress related to restraint and exposure in the nose-only exposure tubes. This was manifested by post-exposure Harderian gland secretion and slightly reduced activity (decreased grooming) (Supplemental Table 10) observed in all exposure groups, including the PBS control group, though with a rapid recovery. After removal of the animals from the exposure tubes following exposure, reduced exploration and gripping ability were observed in rats of both sexes in the majority of exposure groups. Females had slightly higher incidences of delayed righting reflex but recovered rapidly post-exposure. In addition, any animals identified with a condition requiring further evaluation were placed under a clinical observation schedule for daily monitoring. During the course of this study, wounds associated with restraint and movement (tail, forelimb, and hindlimb) in the exposure tubes during exposure were the most prevalent symptoms requiring further clinical observations and were also observed in the PBS-exposed animals (data not shown). In general, the wounds were more prominent in the PG/VG + Nic + F-Med and PG/VG + Nic + F-High groups, and incidences were higher in female rats than males. These wounds were treated using the application of petroleum jelly and/or gauze, generally resulting in improvements in the wounds over the study period.

Ophthalmoscopy was performed at three time points, before exposure (day-3), during exposure (days 76 and 83), and in the post-exposure period (day 130). During the pre-exposure period, there were a few sporadic findings (2 findings in total) of pupil constriction and retinal hemorrhage. The evaluation during the exposure and recovery periods detected one case with iridial changes and one case with vascular proliferation. No abnormalities were seen in the other rats when compared with baseline (i.e., health check animals). These cases appeared to be incidental and therefore were not considered to be exposure-related effects on ophthalmology.

During the course of this study, there were 4 technical deaths, and 4 rats were declared moribund by the Attending Veterinarian and euthanized. These unplanned events were dispersed among the different exposure groups, including 2 rats found dead on study day 1 in the exposure tube, and were considered either incidental or of technical origin.

### Body weight and food consumption

**Fig. 2:**
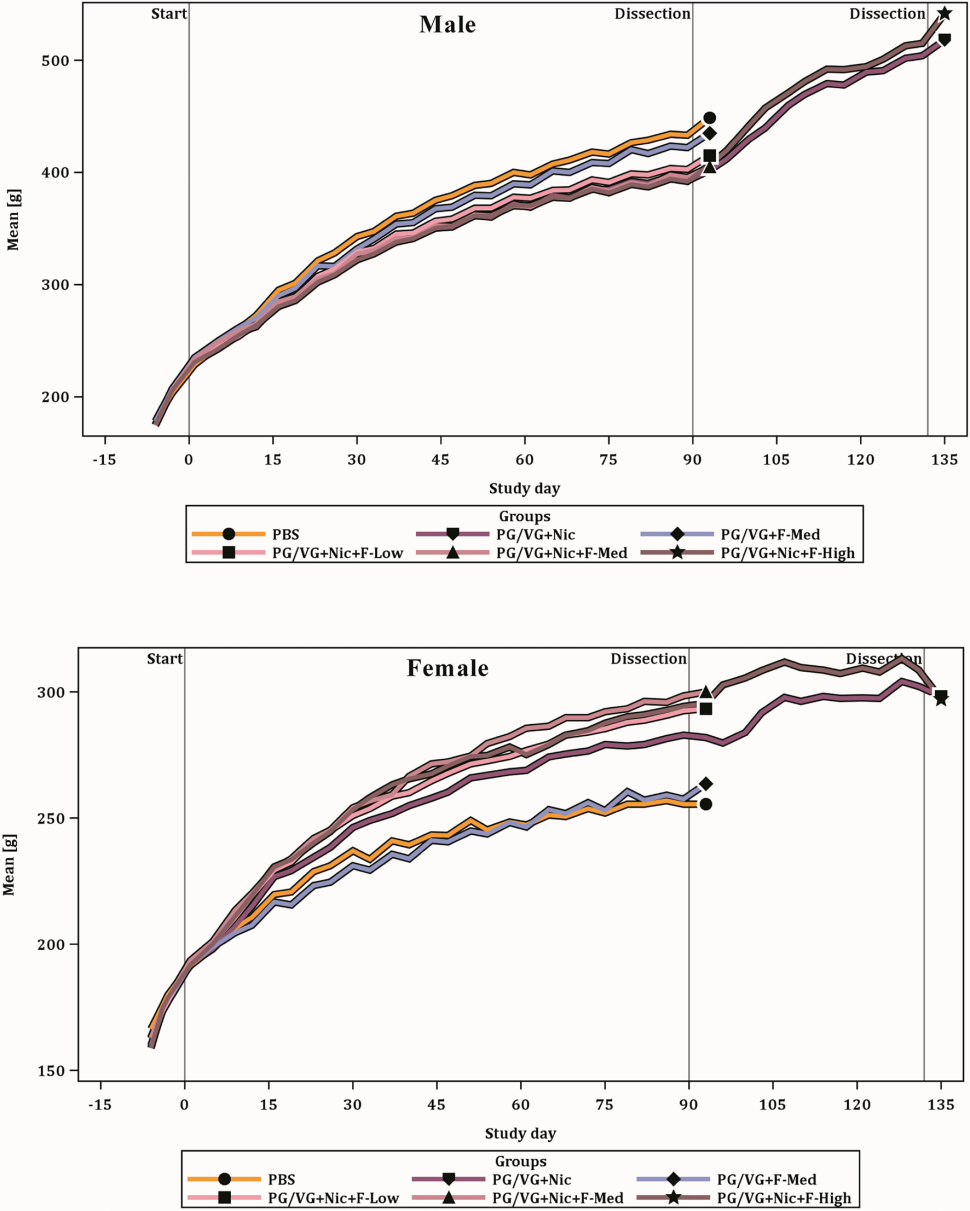
Body weight development. The body weight results for male (top) and female (bottom) rats over time are shown. The results from the similarly exposed OECD, OECD Plus, and Recovery groups were combined for the first 90 exposure days for presentation and for statistical analysis. For additional details, see Table 6

The body weight development over time in the study showed exposure-dependent effects in both male and female rats (Fig. 2). The rats from all exposure groups exhibited a progressive body weight gain over time throughout the 90-day exposure and recovery periods, though the effect of aerosol exposure on body weight was different for the two sexes. Body weights of males in flavored e-liquid groups were similar to that of males in the non-flavored reference (PG/VG + Nic) group, except for higher body weights in the PG/VG + Nic + F-Low group (not statistically significant). The male rats exposed to nicotine-containing aerosols had lower body weights relative to the males exposed to the PBS control and no-nicotine reference (PG/VG + F-Med) aerosols, indicative of lower body weight caused by exposure to nicotine. This trend was different and opposite in the female rats, where female rats exposed to nicotine-containing aerosols had significantly higher body weights compared with those exposed to aerosols from PBS control and no-nicotine reference (PG/VG + F-Med), but this was not flavor concentration-dependent. Male rats had lower body weight gain over time, resulting in lower terminal body weights (approximately 5–9%) when exposed to nicotine-containing aerosols, whereas female rats in the nicotine exposure groups had higher (approximately 11–18%) body weights. Similar nicotine-driven body weight observations for male and females rats were reported in the previous studies (Phillips et al. 2017, 2018).

There was no difference in food consumption between non-flavored e-liquid (PG/VG + Nic) and flavored e-liquid groups (at all concentrations). However, higher food consumption was observed following exposure to nicotine-containing aerosols in female rats (Table 5) compared with the PBS control group, and this was also evident when the medium flavored e-liquid-exposed rats (PG/VG + Nic + F-Med) were compared with rats exposed to no-nicotine (PG/VG + F-Med) reference aerosol. A similar trend (not statistically significant) was seen in male rats and was contrary to lower body weights in male rats when exposed to nicotine-containing aerosols. These results are aligned with those observed in the previous studies, where higher food consumption was seen following exposure to nicotine-containing aerosols, including RRP and nebulized mixture of nicotine (Phillips et al. 2015, 2017, 2018; Wong et al. 2016).

**Table 5:** Food consumption and terminal body weight

Parameter	Sex	Exposed						Recovery	
		PBS	PG/VG + Nic	PG/VG + F-Med	PG/VG + Nic + F-Low	PG/VG + Nic + F-Med	PG/VG + Nic + F-High	PG/VG + Nic	PG/VG + Nic + F-High
Terminal body weight (g)^a^	M	424.8 ± 11.23 (18)	384.8 ± 5.43 (18)**	411.5 ± 14.92 (18)	391.3 ± 10.25 (18)*	382.9 ± 6.95 (18)**	382.7 ± 7.89 (18)**	490.9 ± 19.17 (8)***	500.1 ± 26.77 (8)**
	F	246.4 ± 4.80 (18)	272.9 ± 6.13 (18)**	245.7 ± 4.39 (18)	279.6 ± 5.36 (18)***	286.8 ± 6.11 (18)***	278.8 ± 5.31 (18)***	288.9 ± 7.60 (8)	297.0 ± 7.02 (8)
Food consumption (g/day/100 g of body weight)	M	6.74 ± 0.341 (12)	7.18 ± 0.198 (14)	6.78 ± 0.217 (13)	7.37 ± 0.202 (13)	7.07 ± 0.196 (13)	7.07 ± 0.176 (14)	6.25 ± 0.227 (5)*	5.98 ± 0.241 (5)**
	F	7.53 ± 0.141 (12)	8.02 ± 0.111 (14)*	7.22 ± 0.185 (13)	8.16 ± 0.220 (13)*	8.15 ± 0.148 (13)**	8.39 ± 0.196 (14)**	7.30 ± 0.333 (5)	6.81 ± 0.279 (5)**

Results represent mean ± SEM. The sample size is in parentheses

The Exposed groups are compared against PBS during “Exposed,” while the Recovery groups are compared against the same treatment group from “Exposed.” Significance: *p < 0.05; **p < 0.01; ***p < 0.001

*M* male, *F* female

^a^The body weights presented were collected on study day 91 during “Exposed” and study day 138 during “Recovery.”

### Hematology and clinical chemistry

The red blood cell evaluation revealed minimal changes in the male rats in response to the flavored e-liquid aerosols (PG/VG + Nic + flavors), except a few sporadic changes in hematocrit, mean corpuscular volume (MCV), mean corpuscular hemoglobin (MCH), and mean corpuscular hemoglobin concentration (MCHC) without consistent trend when compared with PBS control and reference aerosols (Supplemental Table 11). Females had significantly higher MCV and MCH and lower MCHC in response to the non-flavored e-liquid and flavored e-liquid (at all concentrations) aerosols (all *p* ≤ 0.01) when compared with PBS control. There were sporadic changes in erythrocyte, reticulocyte, and platelet counts, but these effects were either not consistent or not statistically significant. Minimal flavor-concentration-dependent changes in the total white blood cell counts were observed for flavored e-liquid-exposed groups when compared with the PBS control and non-flavored e-liquid reference (PG/VG + Nic) groups. Changes included lower relative lymphocyte counts in females exposed to flavored e-liquid aerosols at all concentrations (*p* ≤ 0.05) and in males exposed to flavored e-liquid aerosol at high concentration (*p* ≤ 0.01). Alternatively, female rats exposed to nicotine-containing aerosols had higher neutrophil counts (relative, *p* ≤ 0.05) when compared with PBS control, indicating a nicotine effect on this parameter. A similar adaptive nicotine effect on white blood cell counts has been observed in a previously conducted 28-day sub-acute study, where the differences were significant with exposure to a higher nicotine concentration of 50 µg/L that generated a delivered dose of nicotine aerosol equivalent to 13.6 mg nicotine/kg body weight (Phillips et al. 2015). The effects on neutrophils and lymphocytes were resolved after the 42-day recovery period. Counts of other white blood cell types (monocytes, basophils, and eosinophils) showed no obvious trends, and any significant findings were, therefore, considered incidental.

Prothrombin times, activated partial thromboplastin times, and fibrinogen concentrations were evaluated in plasma samples to determine the efficiency of the extrinsic and intrinsic coagulation pathways. Increased prothrombin time (*p* ≤ 0.05) was observed in the male rats exposed to nicotine-containing aerosols (Supplemental Table 11), while there were only limited changes in females from flavored e-liquid-exposed groups when compared with PBS control and non-flavored reference aerosol-exposed rats. Higher prothrombin time and fibrinogen were observed only in females of the PG/VG + Nic + F-Med group when compared with no-nicotine reference group (PG/VG + F-Med) (both *p* ≤ 0.01). These observations were resolved following the recovery period.

There were minimal effects on liver function parameters (Supplemental Table 11) as seen from the clinical chemistry profile in serum. Alanine aminotransferase and alkaline phosphatase activities were higher in females from the groups exposed to flavored e-liquid at low and medium concentrations when compared with PBS control and no-nicotine reference aerosol (*p* ≤ 0.05), while aspartate aminotransferase was significantly lower (*p* ≤ 0.05) in female rats exposed to flavored e-liquid at high concentration versus PBS control. Note that the high aspartate aminotransferase and alanine aminotransferase activities in the PG/VG + Nic + F-High group were mainly attributed to one animal with approximately sevenfold greater values when compared with other rats in the same group. The microscopic observation of the liver for this rat revealed moderate hepatocellular necrosis and vacuolation, in addition to mild hemorrhage, which most likely accounted for these high values. Total protein and albumin levels were lower in females exposed to nicotine-containing aerosols (either non-flavored e-liquid or flavored e-liquid aerosols at all concentrations, *p* ≤ 0.05) compared with the PBS control or no-nicotine reference aerosols. Reduced protein synthesis by the liver in response to nicotine exposure is a possible cause for lower serum albumin and total protein content (Sershen et al. 1982). Subtle changes (lower total protein and globulin) were seen in males exposed to flavors at medium and high concentrations, with or without nicotine, when compared with the PBS control group. There were no significant changes in bilirubin levels in the exposed groups of both sexes compared with either the PBS control or reference exposure groups. In general, the albumin, total protein, and total bilirubin levels were higher in male and female rats following the 42-day recovery period, indicating a possible aging effect.

There were limited exposure-related effects on parameters reflecting renal function (Supplemental Table 11)—mainly lower urea levels in male rats exposed to flavor at low and medium concentrations. The high creatinine and urea levels for females exposed to PG/VG + Nic + F-Med test aerosol are mainly related to high levels observed in one animal (approximately twofold increase when compared with others in the group). However, no histopathological changes were noted in kidneys of this animal, so organ pathology may not be the causal factor for this finding. Parameters of energy metabolism, comprising total cholesterol, triglyceride, and glucose levels, were generally reduced in both sexes following exposure to nicotine-containing aerosols, and these changes were reversed after the recovery period (Supplemental Table 11). These findings are consistent with previous inhalation toxicology studies for nicotine-containing test aerosols or cigarette smoke (Phillips et al. 2015, 2017, 2018; Wong et al. 2016).

There were minimal effects on various electrolyte and mineral levels following flavored e-liquid exposure, with most values showing no obvious trends. Therefore, these changes were considered incidental findings not related to flavored e-liquids exposure. However, inorganic phosphate levels were higher in male rats exposed to flavor mixture at all concentrations (*p* ≤ 0.01), and the levels were inversely proportional to flavor concentrations when compared with the males in the PBS group.

There were limited changes in the myeloid-to-erythroid cell ratio (M/E ratio) from bone marrow evaluation in female rats exposed to flavored e-liquid aerosols, and these changes were not statistically significant when compared with females exposed to either the PBS control or reference groups, although the M/E ratios were higher in females exposed to medium and high concentrations of flavored e-liquid (both *p* ≤ 0.05) than those exposed to low concentration (Supplemental Table 11). The M/E ratios were statistically significantly high in males following exposure to flavored e-liquid at low and medium concentrations, as well as non-flavored (PG/VG + Nic) reference, when compared with PBS control (*p* ≤ 0.01). In general, the M/E ratios decreased following the 42-day recovery period.

### Effects on the respiratory system

**Fig. 3:**
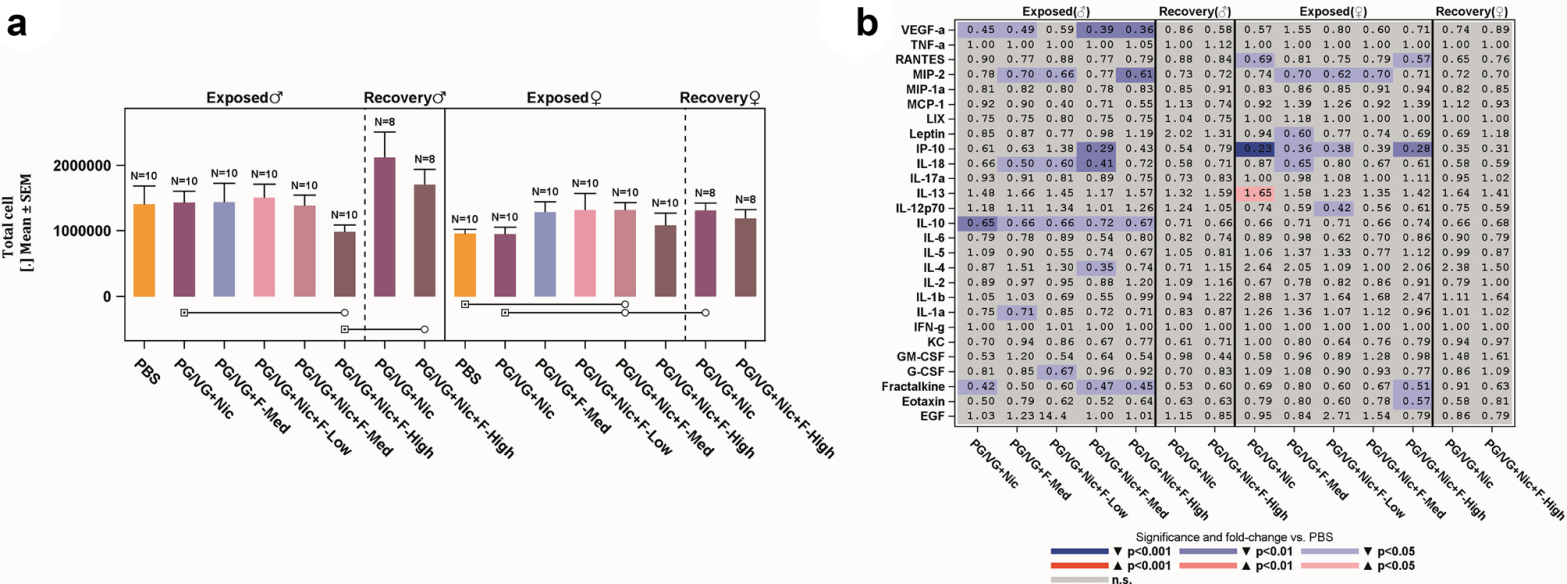
Effects on lung inflammation. The total number of free lung cells recovered from the OECD groups (**a**) and heat map of multiple analyte profile (listed on *y*-axis) in BALF (**b**). Statistically significant differences are represented by empty circles (*p* ≤ 0.05) or filled circles (*p* ≤ 0.01) when compared with the groups indicated by the squares underneath the bars. The numbers in the heat map represent the fold increase relative to the PBS-exposed rats (of similar sex), and significance is indicated using the color scale. For details, see Supplemental Table 12

The exposure to flavored e-liquid aerosols had a minimal effect on the number of free lung cells collected in the BALF, indicating that there was a limited effect on lung inflammation. The only changes were lower total recovered free lung cell count for the males in the PG/VG + Nic + F-High group (*p* ≤ 0.05 compared with non-flavored e-liquid reference) and higher recovered free lung cell count for females exposed to PG/VG + Nic + F-Med aerosol (*p* ≤ 0.05 compared with PBS and non-flavored e-liquid reference) (Fig. 3a). This translated to only minor differences in differential cell counts, without flavor concentration dependency, in rats of both sexes which were considered incidental findings (Supplemental Table 12). Rats had higher total cell and macrophage counts after the recovery period when compared with the exposure-matched groups, probably due to maturation (Petruccelli et al. 2010; Yamamoto et al. 2003).

In addition to free lung cell determination, inflammatory mediator concentrations in cell-free BALF were determined, and the differences from the PBS group are depicted by means of a heat map (Fig. 3b). No clear test or reference item exposure-related changes were detected in the analyte levels, which is consistent with the low levels of recruited inflammatory cells observed in the BALF. Interleukin (IL)-13 was statistically significantly higher (1.65-fold, *p* ≤ 0.01), and interferon gamma-induced protein 10 (IP-10) was statistically significantly lower (0.23-fold, *p* ≤ 0.001), in females following exposure to non-flavored e-liquid reference aerosol (PG/VG + Nic). IL-10, an anti-inflammatory cytokine, was the only analyte whose BALF levels were statistically lower in all males (*p* ≤ 0.05) relative to PBS control following exposure. Other changes in the cytokine concentrations did not show consistent trends that related to any particular exposure conditions and, therefore, were considered as incidental. No effects were observed in the post-exposure/recovery groups. These results indicated that flavored e-liquid aerosol had no significant effect on the absolute or differential cell counts in BALF and minimal/limited effects on the inflammatory mediators in BALF.

**Fig. 4:**
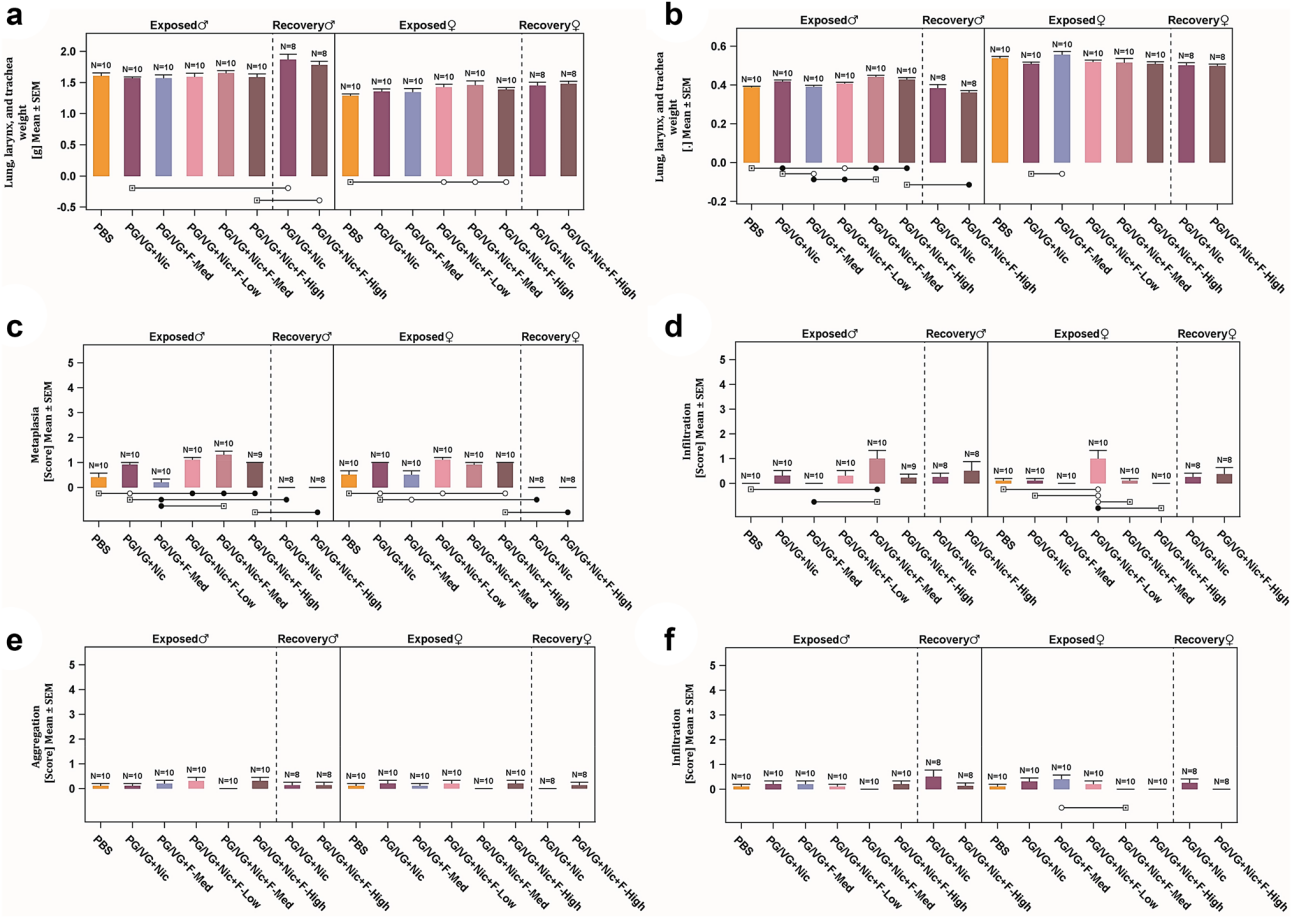
Effects on respiratory organs. The absolute lung (with larynx and trachea) weights (**a**), lung weights (with larynx and trachea) relative to body weights (**b**), and histopathology of the larynx (metaplasia at epiglottis base and infiltration at lamina propria) (**c**–**d**), and histopathology of the lung (macrophage aggregation at alveolar and infiltration of mononuclear or mixed cell at alveolar or perivascular) (**e**–**f**)*.* Statistically significant differences are represented by empty circles (*p* ≤ 0.05) or filled circles (*p* ≤ 0.01) when compared with the groups indicated by the squares underneath the bars

There were very slight exposure-related effects on the absolute and relative weights of the lungs (with larynx and trachea) (Fig. 4a, b). Higher absolute lung weights were observed in female rats of flavored e-liquid groups at all concentrations (*p* ≤ 0.05) compared with the PBS group; however, these effects disappeared after normalization to body and brain weights, except for females from the PG/VG + Nic + F-Low group. Statistically significantly higher lung weights relative to body weights were noted in male rats exposed to nicotine-containing aerosols (*p* ≤ 0.05), indicating a nicotine effect similar to what has been reported previously (Phillips et al. 2017). Inconsistent changes were found in lung weights relative to body weights between males and females, mainly due to the body weight effect following nicotine exposure, as explained. In general, male rats had higher absolute lung weights and lung weights relative to brain weights following the 42-day recovery period, and this effect was minimal in female rats (Supplemental Table 13).

The histopathological evaluation of the respiratory tract tissues showed that lesions were mainly limited to the larynx (Supplemental Table 14). In the nasal cavity, low-severity/incidence lesions were noticed at levels 1–3 of the nose across groups. Sporadic findings (minimal hyperplasia and cell infiltration) were observed in the nasal epithelia in certain 90-day exposure and 42-day recovery groups (both sexes), including PBS control, but these were not statistically significant. Similarly in trachea and lung, findings such as infiltration of mononuclear or mixed cells were observed in a few rats across groups, including PBS control (Fig. 4e, f). In the larynx (base of epiglottis) of both sexes, minimal to mild squamous cell metaplasia (flattening of epithelium, without keratinization) in laryngeal epithelium and infiltration at lamina propria were observed in some animals from all groups, including PBS control (Fig. 4c, d). However, incidence and severity were statistically significantly higher in nicotine-containing groups, particularly in males; therefore, an effect of nicotine or flavoring substances of larynx metaplasia could not be ruled out. The epithelium fully recovered following the 42-day recovery period.

Morphometric analysis of epithelial thickness was performed at three sites of the larynx: base of epiglottis, arytenoid projections, and ventrolateral floor of arytenoid (Supplemental Table 15). There were limited flavored e-liquid exposure-related changes in the epithelial thickness; although there was a slight trend in increase of thickness following nicotine exposure, the majority are not statistically significant, especially in males, and statistical differences are mainly found in females exposed to non-flavored reference and sporadically flavored e-liquid aerosols (*p* ≤ 0.01). Taken together, the quantitative morphometry results were in agreement with the histopathological assessment.

### Transcriptome effects on respiratory nasal epithelium

**Fig. 5:**
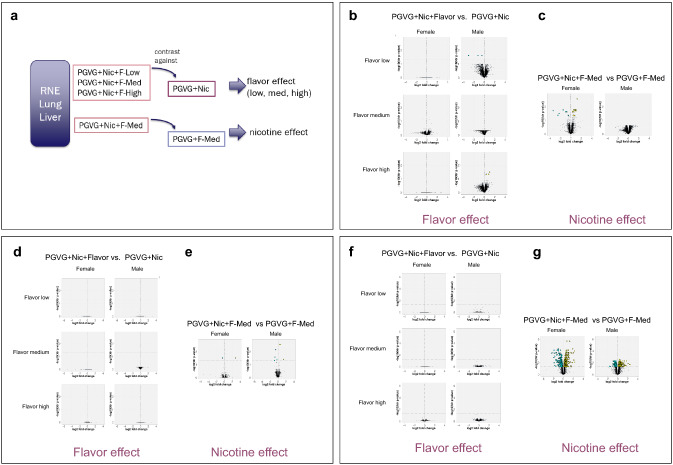
Gene-expression response profiles. Gene-expression comparisons conducted to explore the flavor or nicotine effect (**a**). Volcano plots representing gene-expression changes in RNE (**b**, **c**), lung tissue (**d**, **e**), and liver tissue (**f**, **g**). For each gene, the log2 fold change is represented on the *x*-axis, and the statistical significance, as the negative log10 FDR-adjusted *p* value, is represented on the *y*-axis. Yellow and cyan dots highlight genes that are statistically significantly upregulated and downregulated, respectively (FDR < 0.05)

Consistent with the minimal and histopathological findings in the nasal cavity, transcriptomic analysis of nasal epithelium identified only three significant differentially expressed genes (FDR < 0.05) in the nasal epithelium of male rats exposed to the low and high flavor concentrations when compared with PG/VG + Nic (Fig. 5b and Supplemental Fig. 3A). No differentially expressed genes were detected in those comparisons in the nasal epithelial transcriptome of female rats (Fig. 5b).

When the nasal epithelial transcriptome of the PG/VG + Nic + F-Med-exposed female rat was compared with PG/VG + F-Med to understand the nicotine effect (Fig. 5a), the levels of 23 differentially expressed genes were significantly changed (FDR < 0.05). In the male nasal epithelium, no significantly differentially expressed genes were found in this comparison (Fig. 5c). Biological relevance of these single genes appearing in only one sex and in only one flavor concentration is unlikely.

To further investigate the gene-expression response in the nasal epithelium, gene-set analysis (GSA) was performed to evaluate the responses of predefined biological pathways. GSA is a threshold-free approach to identity significantly affected pathways taking into account the whole gene-expression profiles (Sect. 2.11 above). Addition of flavors to PG/VG + Nic did not significantly affect biological pathways. In contrast, only for female rats, the addition of nicotine (PG/VG + Nic + F-Med versus no-nicotine reference PG/VG + F-Med) yielded gene sets with significant changes between the groups. This included induction of both xenobiotic (e.g., metabolism of xenobiotics by cytochrome P450) and endogenous metabolism gene sets (mitochondrial fatty acid beta oxidation and beta alanine metabolism). Moreover, four immune-related gene sets were significantly downregulated in response to nicotine exposure in female rats (e.g., IL5 pathway) (Supplemental Fig. 4A).

### Transcriptome effects on lung

**Fig. 6:**
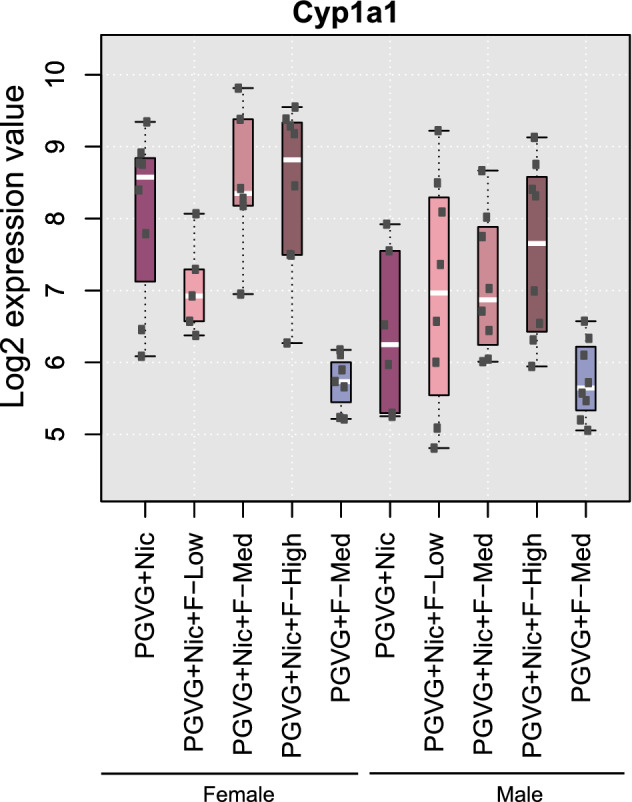
Boxplot of *Cyp1a1* gene expression in lung. Box plots show the median and the first and third quartiles. In addition, the individual data points are shown as black dots

No significant effects of exposure to flavor on the rat lung transcriptome were observed, evidenced by no differentially expressed genes detected in the lungs of rats exposed to flavored PG/VG + Nic compared with rats exposed to the non-flavored reference (PG/VG + Nic) (Fig. 5d). When the lung transcriptome of rats exposed to PG/VG + Nic + F-Med was compared with that of rats exposed to no-nicotine reference (PG/VG + F-Med) to understand the nicotine effect, one upregulated and one downregulated gene were detected in the females, and three upregulated and four downregulated genes were detected in males (Fig. 5e and Supplemental Fig. 3B). In agreement with the previous studies (Phillips et al. 2015, 2017), *Cyp1a1* was significantly upregulated in female lungs for the PG/VG + Nic + F-Med versus no-nicotine reference comparison—with males demonstrating consistent, but non-significant upregulation of Cyp1a1 upon nicotine exposure (Fig. 6). Consistent with the very low gene-level responses, GSA did not yield any gene set with a significant change between the groups (Q2 hypothesis) suggesting that the measured effect on the transcriptome cannot be related to a distinct pathway.

### Effects on non-respiratory organs

In females, absolute and relative (to brain weight) liver weights were higher in rats exposed to nicotine-containing aerosols than those exposed to PBS control and no-nicotine reference (PG/VG + F-Med) aerosols (*p* ≤ 0.01), likely an effect of nicotine exposure. These findings normalized following the 42-day recovery period. However, these effects were only seen in males exposed to flavored e-liquid aerosols at low and medium concentrations after normalization to body weights (*p* ≤ 0.05) when compared with the PBS control group. Similar results were reported in the previous studies when rats were exposed to up to 50 µg nicotine/L (Phillips et al. 2015), where increased liver weights corresponded to vacuolization of the liver when examined microscopically and were considered a metabolic effect on the liver rather than liver pathology. There were no such findings in this study; although nicotine may have had an effect on liver metabolism, this was not reflected by liver vacuolization. Mild-to-moderate subcapsular hepatocellular degeneration was noticed in three males from the PG/VG + Nic + F-High group. The lesions had a specific pattern and were not accompanied by inflammation; thus, they were considered to have been caused by the intraperitoneal injection during terminal anesthesia procedures. Additionally, one female rat in the recovery phase had hepatocellular necrosis with associated inflammation, but the foci were randomly distributed without a specific hepatotoxic pattern. No similar finding was observed for any other 90-day animal. Therefore, this finding was regarded as incidental.

There were limited effects of flavored e-liquid exposure on the kidney weights (Table 6). The males had higher absolute kidney weights and kidney weights relative to brain weights after the recovery period (*p* ≤ 0.05), but the same increase was no longer evident when kidney weights were normalized to body weights and was, therefore, likely due to aging and body weight variance during the 90-day exposure and recovery periods. In females, higher absolute kidney weights and kidney weights relative to brain weights were observed following exposure to flavored e-liquid aerosols (*p* ≤ 0.05 when compared with PBS) as well as for PG/VG + Nic + F-Med versus no-nicotine reference (*p* ≤ 0.01), but the effect was masked by the high body weight gain in nicotine-exposed females. Similarly, the female rats exposed to nicotine-containing aerosols had higher absolute heart weights and heart weights relative to brain weights compared with females exposed to PBS aerosol (*p* ≤ 0.05). This effect was masked by higher body weights of the groups following nicotine exposure and no abnormal pathology findings.

**Table 6:** Exposure-related organ weight changes

Type	Parameter	Sex	Exposed						Recovery	
			PG/VG + Nic	PG/VG + F-Med	PG/VG + Nic + F-Low	PG/VG + Nic + F-Med	PG/VG + Nic + F-High		PG/VG + Nic	PG/VG + Nic + F-High
Absolute weight after exsanguination	Adrenal gland weight (g)	M	↥**	↥	↥**	↥*	↥		↧***	↧**
		F	↥***	↥	↥***	↥**	↥***		↧***	↧***
	Brain weight (g)	M	↧	↧	↧	↧*	↧		↥	↥**
		F	↧	↧	↧	↧	↥		↥	↧
	Epididymis weight (g)	M	↥	↥	↥*	↥	↥		↥*	↥***
	Heart weight (g)	M	↥	↧	↧	↧	↧		↥*	↥***
		F	↥*	↥	↥*	↥**	↥**		↥	↧
	Kidney weight (g)	M	↧	↥	↥	↥	↧		↥**	↥**
		F	↥	↥	↥**	↥**	↥**		↥	↧
	Liver weight (g)	M	↧	↧	↥	↧	↧		↥**	↥*
		F	↥**	↧	↥**	↥**	↥***		↧*	↧*
	Lung, larynx, and trachea weight (g)	M	↧	↧	↧	↥	↧		↥*	↥*
		F	↥	↥	↥*	↥*	↥*		↥	↥
	Ovary weight (g)	F	↥**	↥	↥**	↥	↥**		↧	↧
	Spleen weight (g)	M	↧*	↥	↧*	↧**	↧**		↥***	↥***
		F	↥	↥	↥	↥	↥		↥*	↥*
	Testes weight (g)	M	↥	↥	↥	↥	↥		↥*	↥*
	Thymus weight (g)	M	↧*	↥	↧*	↧**	↧*		↥**	↥*
		F	↧	↥	↧	↧	↧		↥**	↥*
	Thyroid and parathyroid weight (g)	M	↥	↧	↧	↧	↥		↧	↥
		F	↥*	↥	↥	↥*	↥*		↧	↥
	Uterus and cervix weight (g)	F	↥	↥*	↧	↧*	↥		↥	↥*
Weight relative to body weight	Adrenal gland weight	M	↥***	↥	↥***	↥**	↥**		↧***	↧***
		F	↥**	↥	↥**	↥	↥*		↧***	↧***
	Brain weight	M	↥*	↥	↥	↥	↥		↧***	↧***
		F	↧*	↧	↧***	↧***	↧**		↧	↧*
	Epididymis weight	M	↥*	↥	↥*	↥*	↥*		↧	↥
	Heart weight	M	↥*	↥	↥	↥	↥		↧	↧
		F	↥	↥	↥	↧	↧		↧	↧*
	Kidney weight	M	↥	↥	↥*	↥*	↥		↧	↧
		F	↧	↧	↥	↧	↥		↧	↧***
	Liver weight	M	↥	↥	↥*	↥*	↥		↥	↧
		F	↥	↧*	↥**	↥	↥		↧***	↧***
	Lung, larynx, and trachea weight	M	↥**	↥	↥*	↥***	↥**		↧	↧***
		F	↧	↥	↧	↧	↧		↧	↧
	Ovary weight	F	↥	↥	↥**	↥	↥*		↧*	↧
	Spleen weight	M	↧	↥	↧	↧	↧		↥	↥**
		F	↧	↥	↧	↧	↧		↥	↥
	Testes weight	M	↥	↥	↥	↥	↥*		↧*	↧**
	Thymus weight	M	↧	↥	↧	↧*	↧		↥	↥
		F	↧*	↥	↧*	↧*	↧*		↥**	↥*
	Thyroid and parathyroid weight	M	↥	↧	↥	↧	↥		↧	↧
		F	↥	↥*	↥	↥	↥		↧	↧
	Uterus and cervix weight	F	↥	↥*	↧*	↧**	↧		↥	↥
Weight relative to brain weight	Adrenal gland weight	M	↥**	↥	↥**	↥*	↥*		↧***	↧**
		F	↥***	↥	↥***	↥**	↥***		↧***	↧***
	Epididymis weight	M	↥	↥	↥*	↥	↥		↥*	↥***
	Heart weight	M	↥	↥	↧	↧	↧		↥*	↥**
		F	↥*	↥	↥**	↥**	↥*		↥	↧
	Kidney weight	M	↧	↥	↥	↥	↧		↥**	↥*
		F	↥	↥	↥**	↥**	↥*		↥	↧
	Liver weight	M	↧	↧	↥	↥	↧		↥**	↥
		F	↥**	↧	↥***	↥**	↥**		↧	↧
	Lung, larynx, and trachea weight	M	↥	↧	↥	↥	↥		↥*	↥
		F	↥*	↥	↥*	↥	↥		↥	↥
	Ovary weight	F	↥**	↥	↥***	↥*	↥**		↧	↧
	Spleen weight	M	↧	↥	↧	↧*	↧*		↥***	↥***
		F	↥	↥*	↥	↥	↥		↥*	↥**
	Testes weight	M	↥	↥	↥	↥	↥		↥	↥
	Thymus weight	M	↧*	↥	↧*	↧*	↧*		↥**	↥*
		F	↧	↥	↧	↧	↧		↥**	↥*
	Thyroid and parathyroid weight	M	↥	↧	↧	↧	↥		↧	↥
		F	↥*	↥*	↥	↥*	↥*		↧	↥
	Uterus and cervix weight	F	↥	↥*	↧	↧*	↥		↥*	↥*

Symbols, ↓ indicates response lower in Exposed relative to PBS (measured at identical time point); = indicates no difference; ↑ indicates response higher in Exposed relative to PBS (measured at identical time point); while the Recovery groups are compared against the same treatment group from Exposed

Significance: *p < 0.05; **p < 0.01; ***p < 0.001

*M* male, *F* female

For detailed data, see Supplemental Table 13

There were no apparent exposure-related effects on the absolute brain weights or brain weights relative to body weights, except lower relative brain weights in females following exposure to nicotine, compared with female rats in the PBS group (*p* ≤ 0.05). This was most likely a result of nicotine exposure, with females exposed to nicotine having higher body weights. The male rats had significantly lower relative brain weights following the recovery period, which was most likely the result of high body weight gain during the recovery phase (*p* ≤ 0.01).

There were trends of higher thyroid and parathyroid weights, both absolute and relative to brain weights, in female rats exposed to PG/VG-containing aerosols when compared with the PBS control group. No consistent and significant changes were observed in male rats and are, therefore, considered incidental findings.

The uterus weights (both absolute and relative) were lower (*p* ≤ 0.05) for female rats in the PG/VG + Nic + F-Med group, versus PBS and no-nicotine reference (PG/VG + F-Med) groups. The decrease in uterus weight is commonly interpreted as a sensitive endpoint indicating systemic (non-specific) toxicity (Michael et al. 2007); however, the effect was subtle and not concentration-dependent in this study. Exposure of the female rats to nicotine-containing aerosols resulted in numerically higher absolute and relative ovary weights, although the differences were mainly statistically significant after normalization to brain weights (*p* ≤ 0.05). Exposure to nicotine that resulted in lower uterus weights and higher ovary weights in the rats in the current study was also reported after 90 days of exposure in response to conventional cigarettes and heated tobacco sticks (Oviedo et al. 2016; Phillips et al. 2018; Wong et al. 2016) and by others (Iranloye and Bolarinwa 2009; Patil et al. 1999).

**Fig. 7:**
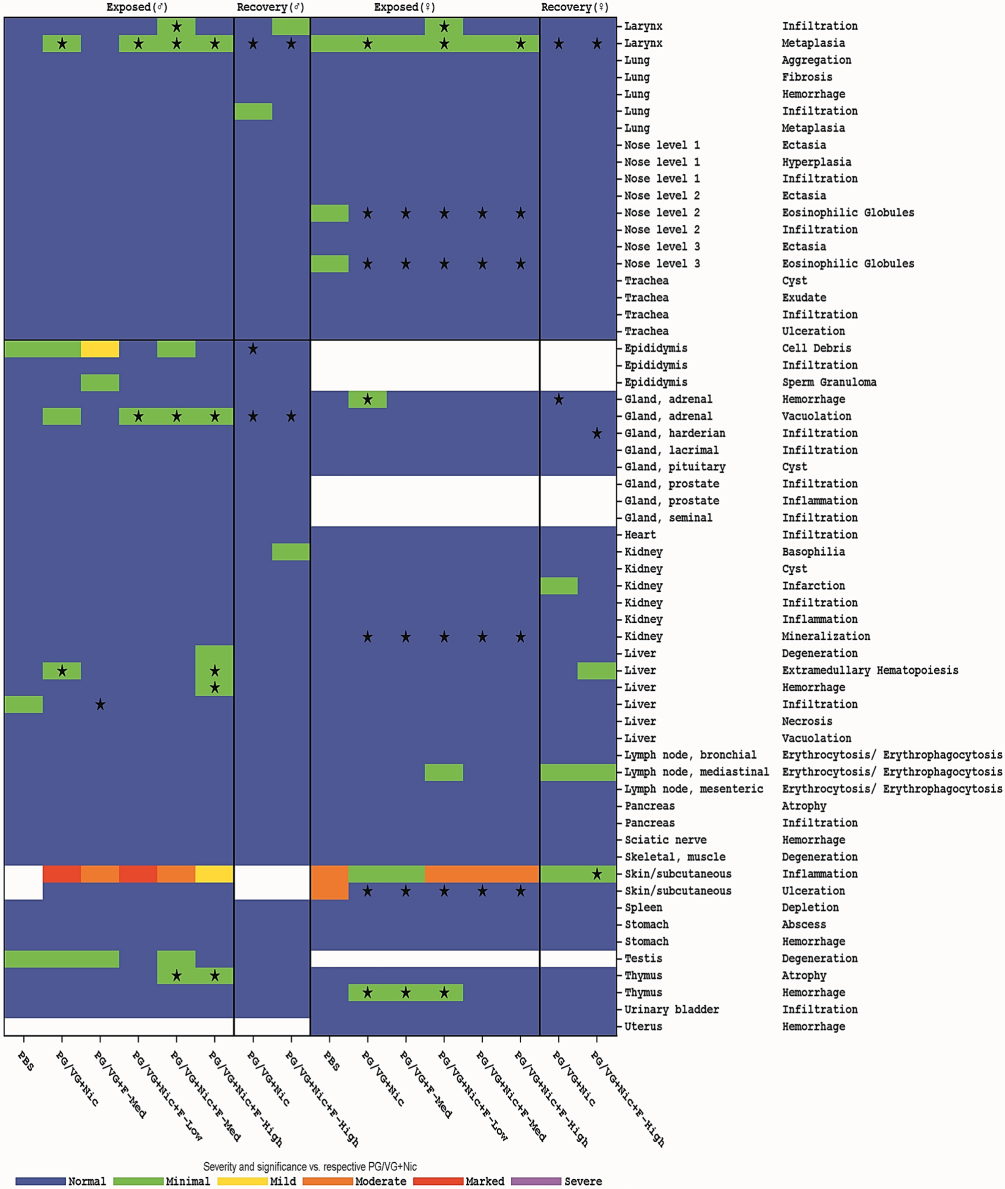
Exposure-related histopathological changes. The histopathological changes in male rats (left) and female rats (right). Median scores were color-coded as follows: [0–0.5] = “Normal;” [0.5–1.5] = "Minimal;” [1.5–2.5] = “Mild;” [2.5–3.5] = “Moderate;” [3.5–4.5] = “Marked; [4.5–5.0] = “Severe.” For exposed groups, the asterisk indicates a statistically significant (*p* < 0.05) difference from the PBS group. For recovery groups, the asterisk indicates a statistically significant (*p* < 0.05) difference from the respective same treatment group during Exposed. Note: signs in the “Normal” fields indicate significantly lower scores/incidences than observed in the comparing group. For a detailed presentation of all histological findings, see Supplemental Table 14

High absolute and relative testis weights were noticed in males following exposure to nicotine, but the differences were only significant when testis weights relative to body weights were compared with rats in the reference groups (non-flavored and no-nicotine aerosols) as well as between rats in the high flavored e-liquid concentration and PBS control groups (both *p* ≤ 0.05). A similar nicotine-related effect was also reported in the previous studies (Phillips et al. 2017, 2018; Wong et al. 2016). The testis weights relative to body weights were lower after the recovery period, but this decrease was not observed for absolute testis weights or testis weights relative to brain weights, thus likely to be attributed to body weight gain in males during the recovery period. Pathological evaluation of testis in this study revealed the cases of bilateral tubular degeneration independent of study group (not statistically significant) following the 90-day exposure, probably a consequence of restraint rather than exposure. In addition, cell debris was recorded in bilateral epididymis as a consequence of tubular degeneration (Fig. 7). Tubular degeneration and epididymal changes attributed to the injury caused by the confinement of rats in nose-only inhalation exposure tubes were consistent to procedure-related effects described in the literature (Rothenberg et al. 2000). Therefore, subtle changes in testis weights could not be concluded as a nicotine effect reported previously for orally or subcutaneously administered nicotine (Aydos et al. 2001; Oyeyipo et al. 2010); however, the testicular degeneration is consistent with the phenomenon reported for restrained rats, even in controls for inhalation exposure (Lee et al. 1993).

### Systemic stress responses

The adrenal weights, absolute and relative to body and brain weights, were higher following exposure to nicotine-containing aerosols for male and female rats when compared with the PBS groups (*p* ≤ 0.05). This finding was no longer observed after the 42-day recovery period (Table 6). A similar finding was observed in females between PG/VG + Nic + F-Med and no-nicotine reference (PG/VG + F-Med) groups (*p* ≤ 0.01) for absolute weights and after normalization to brain weights. These effects were more pronounced in females than in males and could reflect a general nicotine aerosol inhalation-related effect plus an additional exposure-related stress factor. During histopathological evaluation, bilateral cortical vacuolation was seen in some males, but not in females, following the 90-day exposure, while mild adrenal hemorrhage was observed in some female rats, but was found in only one male rat. Chronic stress may impact organ weights, and increased adrenal gland weights were reported previously (Everds et al. 2013). In this study, the procedure-related stress during exposure was fully reversible following the 42-day recovery period.

Lower thymus weights, both absolute and relative to brain weights, were found in males exposed to nicotine-containing aerosols when compared with the PBS control group (*p* ≤ 0.05) (Table 6). A similar trend was noticed in females, but this was not significant, with the exception of thymus weights relative to body weights (*p* ≤ 0.05) when a comparison was made with those exposed to PBS control and no-nicotine reference (PG/VG + F-Med) aerosol, which likely indicates a body weight effect in female rats. Histopathological evaluation revealed minimal lymphoid atrophy in both sexes from all groups, and this change was considered stress-related, as the thymus is the lymphoid organ most sensitive to stress (Everds et al. 2013). A similar effect was observed in previous 90-day rat inhalation studies (Oviedo et al. 2016; Phillips et al. 2017; Wong et al. 2016), where lower thymus weights were detected in rats exposed to nicotine-containing aerosols, although not in a concentration-dependent manner. The findings were no longer observed after the recovery period.

Both absolute and relative spleen weights were lower in the male rats exposed to nicotine-containing aerosols, and it was statistically significant when PG/VG + Nic + F-Med was compared with no-nicotine reference (PG/VG + F-Med). A similar observation was made for spleen weights relative to body weights in females (*p* ≤ 0.01). These changes were resolved during the recovery period (Table 6). Similar results were reported in a previous study (Phillips et al. 2015), along with decreased weight of the thymus, and are considered to be a procedure-related stress response.

### Transcriptome effects on liver

No differentially expressed genes were identified in the livers of rats exposed to flavored e-liquids compared with rats exposed to non-flavored reference (PG/VG + Nic) (Fig. 5d), indicating that the flavor mixtures do not have an impact on the liver transcriptome. In contrast, consistent with a previous nicotine exposure study (Phillips et al. 2017), an effect of nicotine on the liver transcriptome was evident. When the liver transcriptome of rats exposed to PG/VG + Nic + F-Med was compared with that of rats exposed to no-nicotine reference (PG/VG + F-Med), 481 upregulated and 283 downregulated genes were detected in the females, and 281 upregulated and 145 downregulated genes were detected in males (Fig. 5d and Supplemental Fig. 3C).

Consistent with the gene-level results, no statistically (Q2 hypothesis) differentially affected enriched gene sets were found between the flavored e-liquids and non-flavored reference groups (Supplemental Fig. 4B). However, many gene sets were significantly affected in the livers of female and male rats exposed to PG/VG + Nic + F-Med versus no-nicotine reference, with many gene sets significantly enriched both in the Q1 and Q2 hypothesis. The enriched gene sets were related to various different cellular processes, highlighting cholesterol biosynthesis (upregulated), metabolism of xenobiotics (upregulated), and antigen processing and presentation (downregulated).

## Discussion

### Use of flavor toolbox in toxicity assessment

The use of flavoring substances in RRPs is essential to improve product acceptance by adult smokers and encourage switching from cigarettes. However, in light of the large number of flavoring substances that can potentially be used in RRPs and the necessity to acquire safety data on flavoring substances by inhalation, the classical approaches, where flavoring substances are evaluated for toxicity one-by-one, are not suitable, because they would require years and thousands of animals to be completed. The first step in the assessment of toxicity of flavoring substances involves determination of the effect of neat flavoring substances, that is, without potential changes related to thermal treatment during aerosol generation; therefore, a nebulization approach was used in this study.

In this work, we proposed a combinatorial flavor group-based approach using both standard and systems toxicological methods to acquire information on the safety of flavoring substances via inhalation. Under this approach, the flavoring substances have been clustered in groups of structurally related substances that are expected to exhibit similar metabolic and biological properties. Then, FGRs with the predicted worst toxicological profile within each chemical group were chosen. In particular, 28 FGRs have been identified and tested as a mixture in this 90-day rat inhalation study. This in vivo study allowed the definition of acceptable use levels (AUL) for FGRs. Finally, toxicological information acquired on FGRs could be used to predict the toxicity of structurally related flavoring substances within the same group and set AULs. This concept is based on the foundational principle of the read-across approach, where available data of a data-rich substance (the source) are used for a data-poor substance (the target) that is considered similar enough to the source substance, and use the same data as a basis for the safety assessment (Schultz et al. 2015).

This approach has the advantage to drastically reduce the time to generate data on a large number of chemicals, while at the same time minimizing the number of animals used for the assessment during product design. However, this approach also has some limitations. First, the clustering approach relies entirely on the chemical groups defined by EC No 1565/2000, which are somewhat general. Therefore, a refinement or subgrouping may be considered in future studies. Secondly, FGRs were selected mostly using predicted toxicological data; therefore, in the absence of experimental values, it mostly relies on predictions for the selection of the most toxic substance of each group. Finally, it is unlikely that FGRs in the mixture equally participate to the overall mixture’s toxicity. This means that the toxicity of the most toxic flavoring substances in the tested mixture is driving the definition of safety levels of the less-toxic substances and may lead to the establishment of overly conservative levels for non-toxic flavoring substances.

E-cigarettes function by heating an e-liquid to generate an aerosol (commonly called vapor), which users inhale. Besides transferring the e-liquid compounds in the aerosol, this thermal process may indeed also generate other compounds or new toxic constituents. This, however, depends on the device or technology used to produce the aerosol. With the current technology used in e-cigarettes, the temperature of the heater can vary significantly depending on how the e-cigarette is puffed on as well as the temperature profile in the device. Nevertheless, the bulk composition of the aerosol should contain the base components including the flavoring substances added to the base solution of e-liquid (nicotine, PG, and VG). Therefore, it is critical to assess the potential intrinsic toxicity of the flavoring substances generated through nebulization—independent of thermal treatment for aerosol generation and/or diverse aerosol generation devices—as done in this study. However, in a subsequent step, a thorough analysis of the toxicity of the aerosol formed upon thermal treatment of the substances in e-liquid formulations will be performed to supplement the dataset provided by this study and to understand whether new chemicals are generated through liquid component reactions and/or pyrolysis.

### Exposure and biological effects

The exposures of the different study groups in this study were well executed, with high consistency in the aerosol constituent concentrations monitored at the exposure chamber throughout the study, and through confirmation of uptake of selected substances in blood and urine. There was no ocular irritation, and no remarkable exposure-related in-life changes were observed in this study. The rats showed the typical signs of stress related to restraint and exposure in nose-only exposure tubes, with high frequency of post-exposure Harderian gland secretion and low frequency of reduced grooming in the different exposure groups, with no flavor-specific effects. Similarly, the individual observations showed sporadic-reduced gripping ability in both sexes and higher incidences of delayed righting reflex in females after removal from exposure tubes. The body weights of rats increased progressively over the study period, though exposure to nicotine had different effects on the body weight trends of both sexes, there was no flavoring concentration-dependent effect. Higher food consumption paralleled higher body weights in females exposed to nicotine; however, the same effect was not seen in males. Similar changes were observed in the previous in-house 90-day rat inhalation studies. Weight gains that were observed during the recovery period in both genders of all groups underlined the exposure- and tube restraint-related stress on body weights.

In this study, laryngeal squamous cell metaplasia was observed at the base of the epiglottis in all groups, with severity ranging from minimal to mild. The larynx is the most sensitive site for detecting the irritation-based effects of inhaled aerosol particles in the respiratory tract (Osimitz et al. 2007). A similar adaptive response was observed after exposure to the vehicle control and nicotine (Phillips et al. 2015), PG (Werley et al. 2011), and VG (Renne et al. 1992); this response was mainly associated with a sensitive adaptive endpoint to a wide range of conditions in inhalation studies, including dehydration by low-humidity air, rather to than a toxicologically relevant response (Burger et al. 1989; Osimitz et al. 2007). These effects following exposure to the e-liquids were considered mild irritation effects by contrasting the findings with those observed after exposure to cigarette smoke from cigarette in a comparable exposure regimen for rats (Oviedo et al. 2016; Phillips et al. 2018; Wong et al. 2016).

Low levels of flavor, such as menthol, suppressed respiratory irritation by smoke irritants (Ha et al. 2015), while the other flavoring substances may contribute to irritation of respiratory airways (Goniewicz et al. 2014; Leigh et al. 2016; Soussy et al. 2016). No irritation effects following exposure to flavored e-liquids were seen in this study, as evidenced by the absence of changes in the respiratory physiology measurements. In addition, the absence of lung inflammation in response to the flavors, at all concentrations, is also indicative for the lack of irritation (Osimitz et al. 2007). This is in agreement with the pathological assessment, in which only minimal macroscopic and microscopic findings were identified in the lungs of the rats across different groups, while measurement of inflammatory mediators in the cell-free BALF also confirmed that there was little effect of the flavoring substances on pulmonary inflammation markers. It has been reported previously (Gerloff et al. 2017) that e-cigarette liquids/aerosols containing flavoring substances induced a pro-inflammatory cytokine response in vitro in lung epithelial cells. The local concentrations of the flavors in the lung tissue in the current in vivo study, and also in e-cigarette users, are probably much lower than those that elicited an inflammatory response in the cited in vitro study. Moreover, the fact that the FGR mixture used did not cause any changes in the nasal epithelia, where a much higher local concentration of flavoring substances can be expected than in the lung, shows that the FGR mixture used did not cause inflammation. Extrapolation of in vitro data to more complex biological systems should thus be done with care.

Transcriptomics analysis of RNE, lung, and liver revealed a very low or absence of effect of flavoring substances. For reference, upon conventional cigarette smoke exposure (at similar nicotine concentration), transcriptomics analysis of RNE commonly identifies several thousand genes as differentially changed (Oviedo et al. 2016). In the current study, the only significant changes in expressed genes were found in the RNE of male rats when the transcriptome of PG/VG + Nic added with low or high concentrations of flavoring substances was compared with the transcriptome of the RNE after PG/VG + Nic exposure. Biological relevance of these single genes appearing in only one sex and in only one flavor concentration is unlikely. Gene set enrichment analysis confirmed that flavor addition to PG/VG + Nic did not induce significant gene sets (Q1 and Q2 hypothesis) in the nose, lung, and liver. This finding suggests that the genes that changed even below the significant threshold upon flavor exposure do not play a role in a common pathway. Thus, in the current study, the flavoring substances added to PG/VG + Nic did not induce a measurable biological response on the transcriptome level.

Together, the data indicate that the inhalation of flavoring substances caused very minimal local and systemic toxic effects in rats, such as changes in blood chemistry (globulin, total protein, and inorganic phosphate) and higher liver weight in males. There were also minimal effects on the red blood cell parameters in both sexes from all exposure groups. The evaluation of the differential leukocyte counts revealed only minor nicotine-related effects, namely high relative neutrophil and low relative lymphocyte counts, especially in female rats. In addition, there was a nicotine effect on several clinical chemistry parameters, namely liver enzyme activities, protein synthesis, and energy metabolism, in female rats and occasionally in male rats, without obvious contribution of the flavor mixture at any concentration, and this effect was reversed following the 42-day recovery period.

Metabolic shifts following nicotine exposure in female rats were reported in the previous study (Phillips et al. 2017). Increased food consumption that was associated with increased body weights in female rats, and decreased blood concentrations of glucose, cholesterol, and triglycerides in both sexes, were consistent with the observations in this study. Systemic response to immobilization stress or chronic stress causing metabolic changes were observed (de Oliveira et al. 2014; Ricart-Jan et al. 2002); however, other non-inhalation nicotine exposure studies reported increases in lipid and cholesterol levels in plasma (Ashakumary and Vijayammal 1997; Chattopadhyay and Chattopadhyay 2008; Waldum et al. 1996). Therefore, the differences in physiological states are likely dependent on the nicotine exposure route, concentrations, and duration, potentially interlinked with complex nicotine effects on nervous system and the general exposure stress.

Nicotine exerts complex effects on the stress response and affects different stress regulation systems, predominantly the hypothalamic–pituitary–adrenal (HPA) axis (Faraday et al. 2005; Matta et al. 1998; Rhodes et al. 2001). In this study, the effect of exposure on organ weights is similar to the previous sub-chronic studies (Oviedo et al. 2016; Phillips et al. 2015, 2017; Wong et al. 2016) as a result of general exposure stress due to restraining in the nose-only method. The changes including altered thymus, spleen, and adrenal weights, increased neutrophils with decreased lymphocytes, and in-life post-exposure observations that were interpreted as secondary responses to stress. Sensory stimuli can cause increased catecholamine release and activation of the HPA axis, thereby increasing serum glucocorticoid levels, which may contribute to a variety of downstream stress responses (Everds et al. 2013). Nicotine-mediated adaptive response following exposure to cigarette smoke, aerosols from RRPs, and nicotine-containing aerosols also support the hypothesis of an HPA-mediated general exposure stress seen in this study (Mendelson et al. 2005; Oviedo et al. 2016; Phillips et al. 2015, 2017, 2018; Wong et al. 2016).

Likely reflecting this systemic response to nicotine exposure, among the three tissues, liver displayed the most pronounced transcriptome effects for the PG/VG + Nic + F-Med versus no-nicotine reference comparison. Consistent with a previous study (Phillips et al. 2017), several metabolic processes were affected by nicotine exposure (Supplemental Fig. 5). For example, in this study, GSA analysis determined steroid biosynthesis and biosynthesis of cholesterol, a precursor for the biosynthesis of steroid hormones, as highly affected pathways in both genders. In blood, total cholesterol levels in both sexes following exposure to nicotine-containing aerosols were lower, possibly reflecting a related metabolic alteration.

## Conclusion

In this study, the biological effects on Sprague–Dawley rats of inhaled aerosols generated by nebulization of flavored e-liquids were evaluated and compared with those of non-flavored e-liquid and nicotine-free references, according to OECD TG 413. Standard toxicological endpoints were complemented with system toxicological analyses using transcriptomics of respiratory nasal epithelium, lung, and liver tissues. Both standard and systems toxicology endpoints demonstrated very limited biological effects of the tested FGR mixture. When tested at elevated levels in excess of those typically used in commercial products, the FGR mixture did not increase the inhalation toxicity. Several adaptive changes due to nicotine exposure have been observed, but no major adverse effects were caused by the FGRs in e-liquid. Because of the study design with multiple FGRs in the mixture, it can be concluded that there were no apparent additive or synergistic toxic effects among the flavoring substances as they were tested. Systemic effects due to constraint stress following inhalation modalities and the nicotine effects have been shown to be reversible on cessation of treatment, except for testicular degeneration. There were no changes in the severity or incidence of respiratory tract findings, and no new target organ toxicity was identified in this study. These results are consistent with the findings of 90-day inhalation studies (Heck et al. 1998; Vanscheeuwijck et al. 2002). This innovative and practical flavor toolbox approach to screen and perform toxicological risk assessment of FGR mixture encompasses a comprehensive strategy for responsible selection of flavoring substances and their threshold levels of usage in RRPs, and this is guided by toxicological principles to fill the data gaps of ingredients added to tobacco- or nicotine-containing products or delivery systems.

## Electronic supplementary material

Below is the link to the electronic supplementary material.

Supplementary file1 (PDF 1411 kb)

Supplementary file2 (PDF 2416 kb)

Supplementary file3 (PDF 319 kb)

Supplementary file4 (PDF 166 kb)

Supplementary file5 (PDF 323 kb)

Supplementary file6 (PDF 19 kb)

Supplementary file7 (DOCX 256 kb)

Supplementary file8 (PDF 281 kb)

## Abbreviations

AAALAC: American Association for the Accreditation of Laboratory Animal Care
ABF: Analytisch-biologisches Forschungslabor GmbH
AB-PAS: Alcian blue/periodic acid–Schiff's stain
ALP: Alkaline phosphatase
ALT: Alanine aminotransferase
APTT: Activated partial thromboplastin time
AST: Aspartate aminotransferase
AU: Acceptable use level
AVS: Animal and Veterinary Service
BALF: Bronchoalveolar lavage fluid
CMR: Carcinogenic, mutagenic, or reprotoxic
COT: Cotinine
EC: European Commission
ECHA: European Chemical Agency
EU: European Union
F: Flavoring substances
FA: Formaldehyde
FDR: False discovery rate
FG: Food-grade
FGR: Flavor group representatives
FPC: Flow-past chamber
GLP: Good Laboratory Practice
GRAS: Generally recognized as safe
GSA: Gene-set analysis
GSD: Geometric standard deviation
H&E: Hematoxylin and eosin
Hb: Hemoglobin
HBSS: HEPES-buffered saline solution
HCT: Hematocrit
HOCOT: Trans-3′-hydroxycotinine
HPA: Hypothalamic–pituitary–adrenal
HPLC: High-performance liquid chromatography
IL: Interleukin
IP: Interferon gamma-induced protein
ISO: International Organization for Standardization
LC–MS: Liquid chromatography–tandem mass spectrometry
LC_50_: Median lethal concentration
LD_50_: Median lethal dose
LOAEL: Lowest observed adverse-effect level
LOD: Limit of detection
LOQ: Lower limit of quantification
MCH: Mean corpuscular hemoglobin
MCHC: Mean cell hemoglobin content
MCV: Mean corpuscular volume
MMAD: Mass median aerodynamic diameter
NACLAR: National Advisory Committee for Laboratory Animal Research
NCOT: Norcotinine
Nic: Nicotine
NNIC: Nornicotine
NNO: Nicotine-*N*′-oxide
OECD: Organization for Economic Cooperation and Development
PBS: Phosphate-buffered saline
PG: Propylene glycol
PIF: Peak inspiratory flow
PMI: Philip Morris International
PT: Prothrombin time
QSAR: Quantitative structure–activity relationship
RBC: Red blood cells/erythrocytes
REACH: Registration, Evaluation, Authorisation and Restriction of Chemicals
RF: Respiratory frequency
RLE: Relative log expression
RNA: Ribonucleic acid
RNE: Respiratory nasal epithelium
RRP: Reduced-risk products
SD: Standard deviation
SEM: Standard error of mean
TPM: Total particulate matter
TTC: Threshold of toxicological concern
TV: Tidal volume
UK: United Kingdom
USA: United States of America
VG: Vegetable glycerin

## Footnotes

1 "Reduced-Risk Products" or "RRPs" is the term which we use to refer to products that present, are likely to present, or have the potential to present less risk of harm to smokers who switch to these products versus continued smoking. We have a range of RRPs in various stages of development, scientific assessment, and commercialization. Because our RRPs do not burn tobacco, they produce far lower quantities of harmful and potentially harmful compounds than found in cigarette smoke.

2 A study generally expected to be the most adequate, reliable, and relevant for a specific element/endpoint. If properly reported, key study may fulfill a REACH information requirement on its own.
