# Supplementary figures and images for: Evaluation of toxicity of aerosols from flavored e-liquids in Sprague–Dawley rats in a 90-day OECD inhalation study, complemented by transcriptomics analysis
Source: Arch Toxicol. 2020 May 5;94(6):2179–206. doi: 10.1007/s00204-020-02759-6 (PMC7303093; doi:10.1007/s00204-020-02759-6)

A

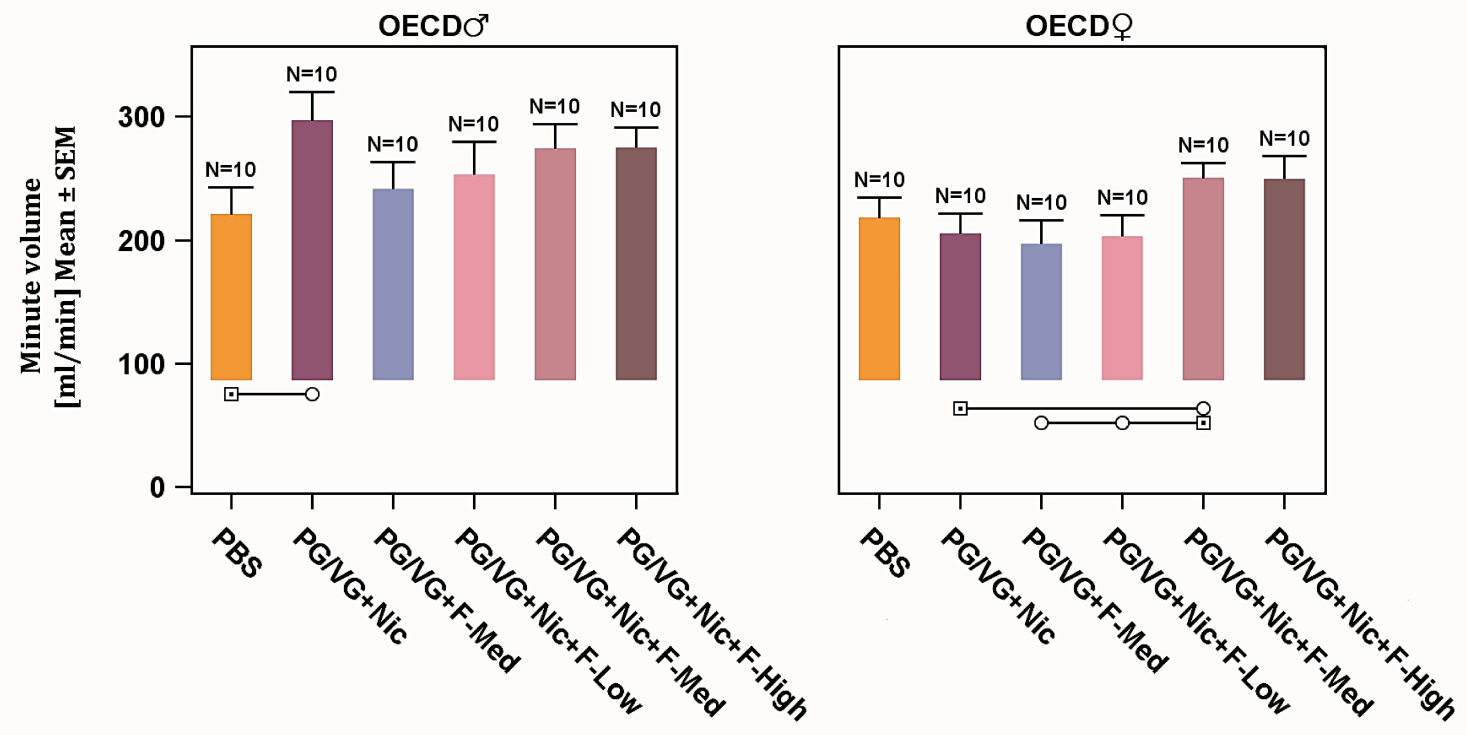

B

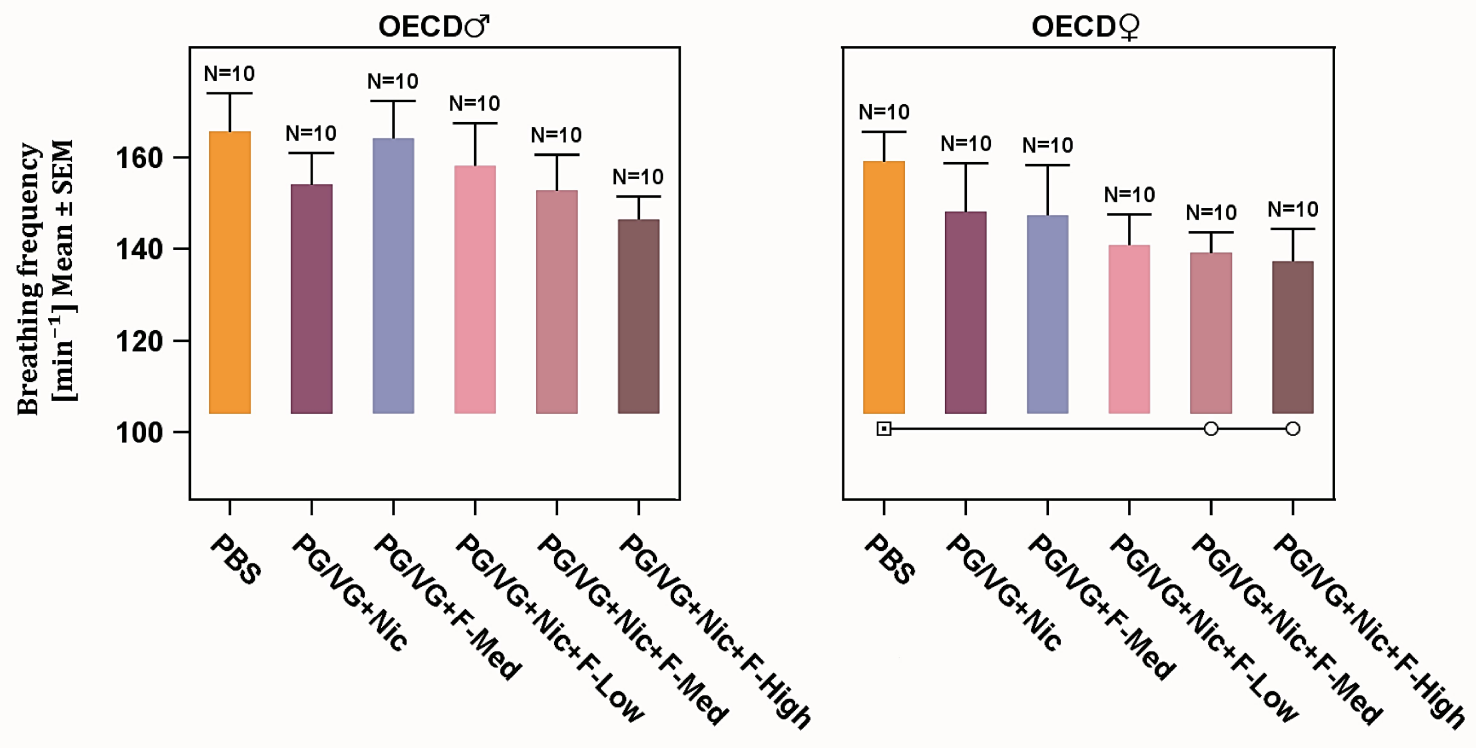

Supplement: Supplementary file 1 — Supplementary file1 (PDF 1411 kb) [file 204_2020_2759_MOESM1_ESM.pdf]

A

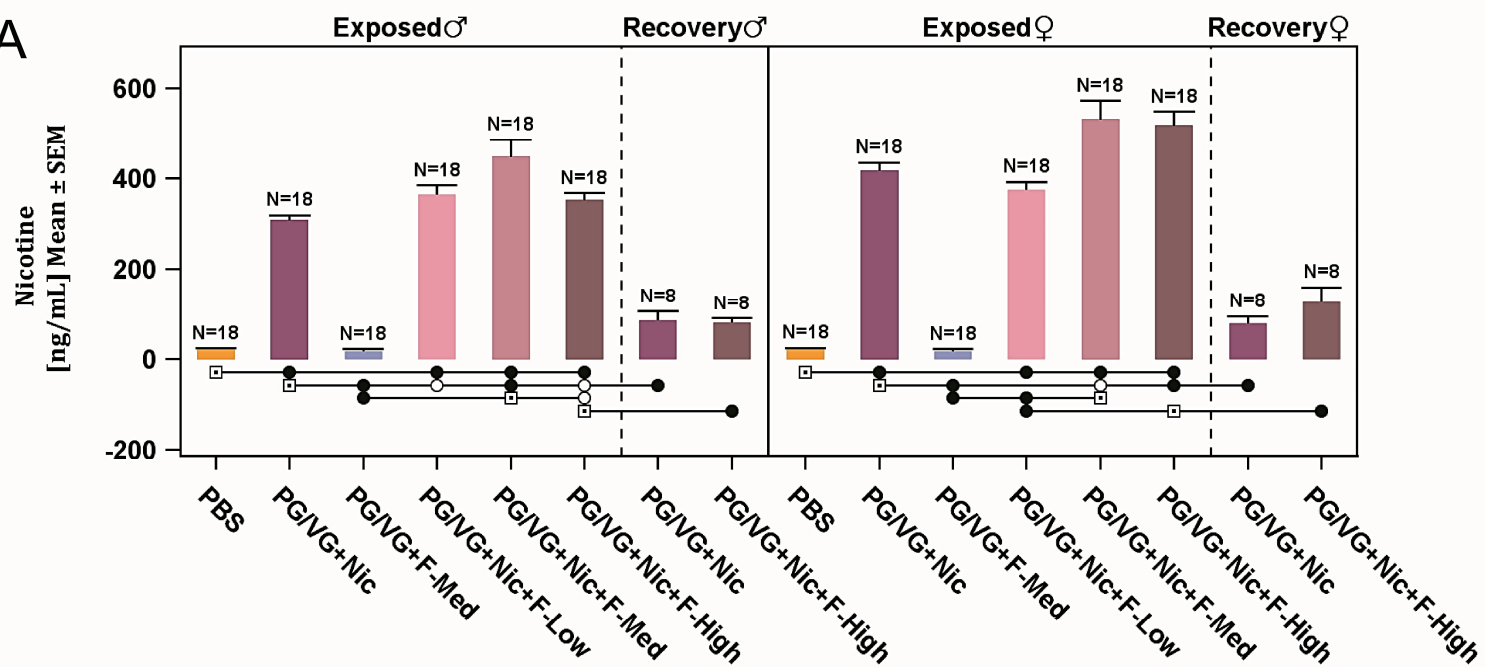

B

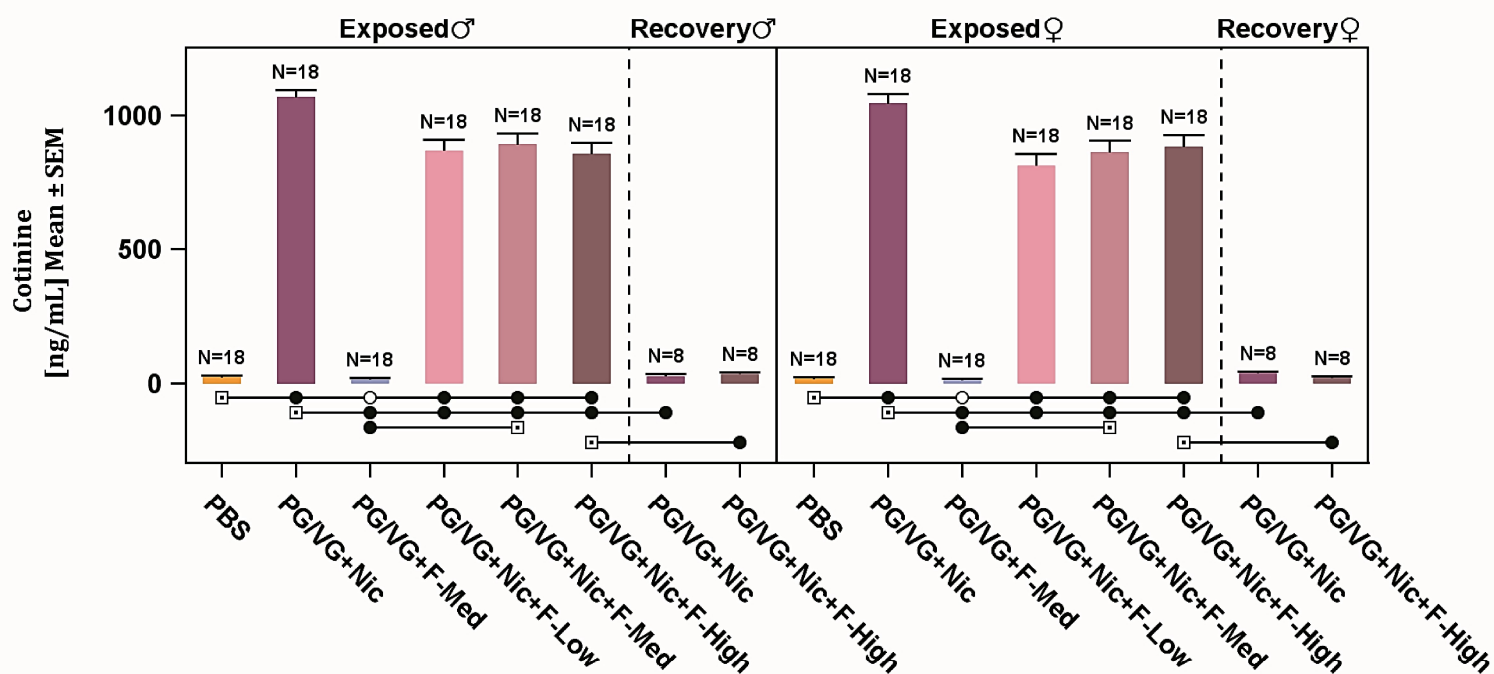

C

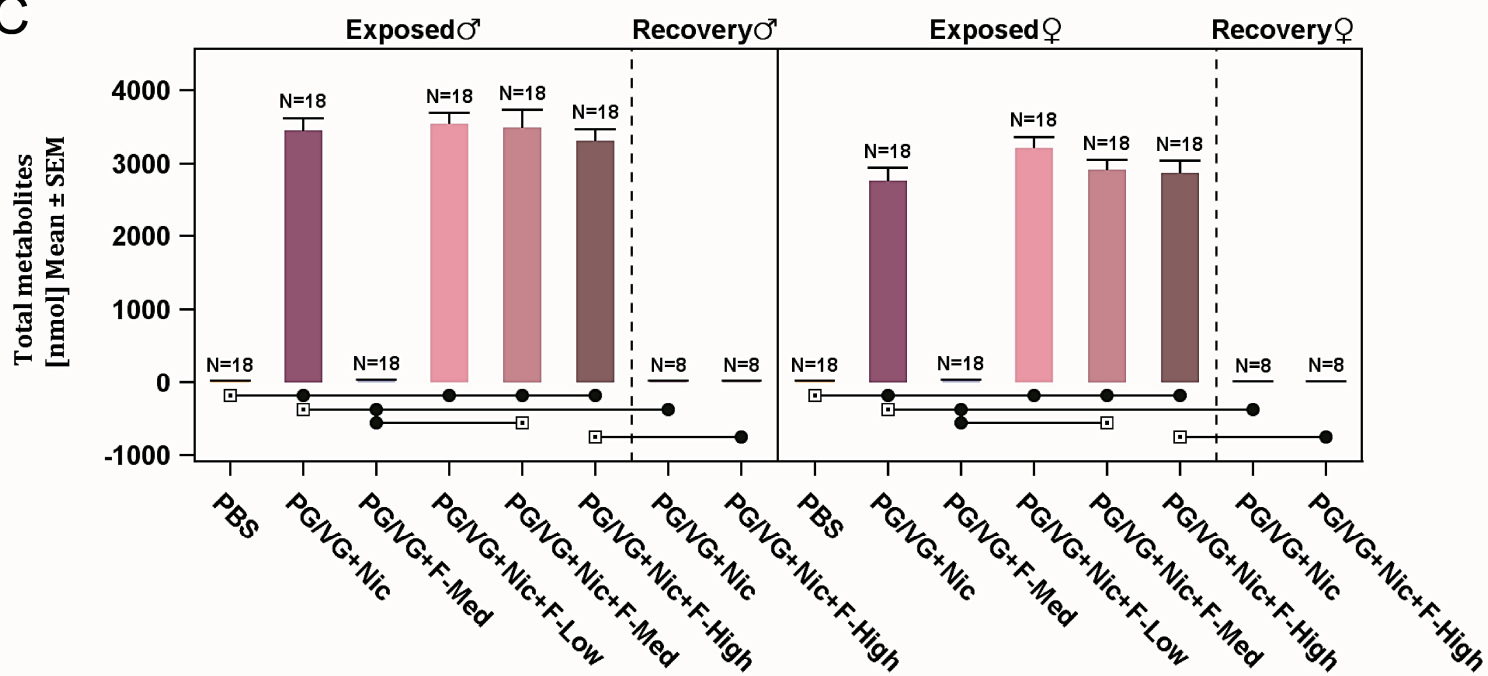

Supplement: Supplementary file 2 — Supplementary file2 (PDF 2416 kb) [file 204_2020_2759_MOESM2_ESM.pdf]

A

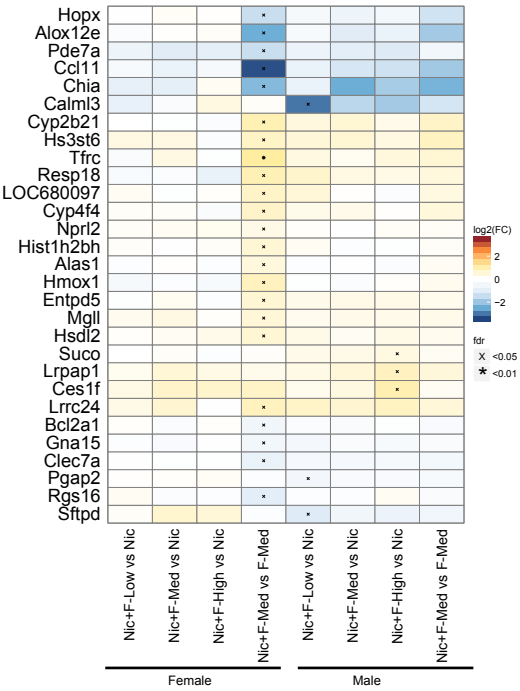

B

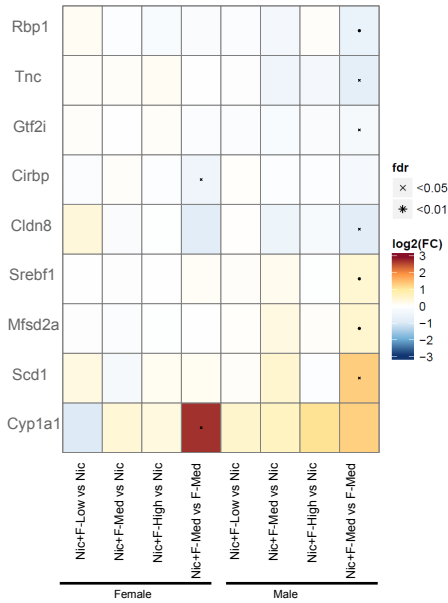

C

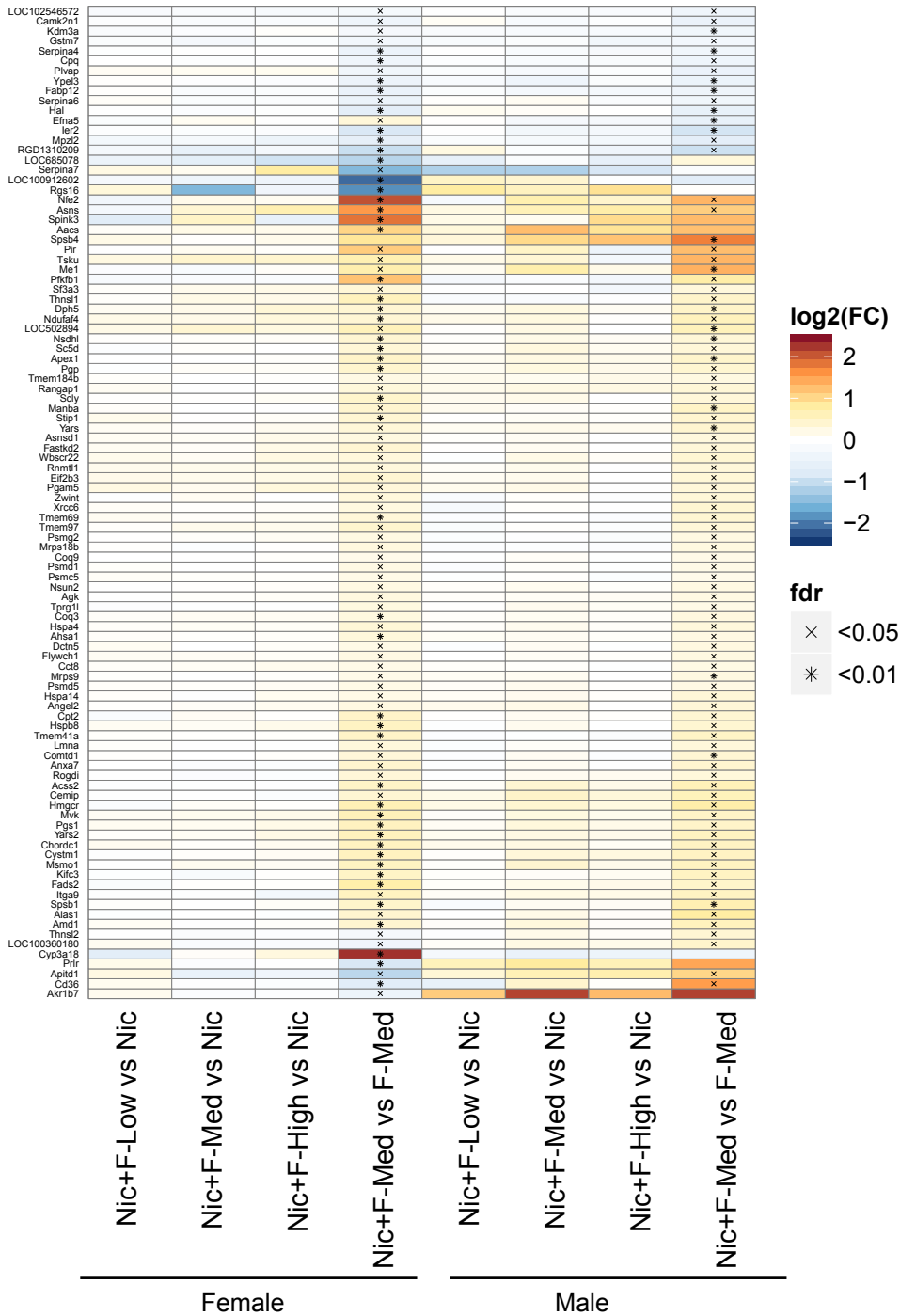

Supplement: Supplementary file 3 — Supplementary file3 (PDF 319 kb) [file 204_2020_2759_MOESM3_ESM.pdf]

A

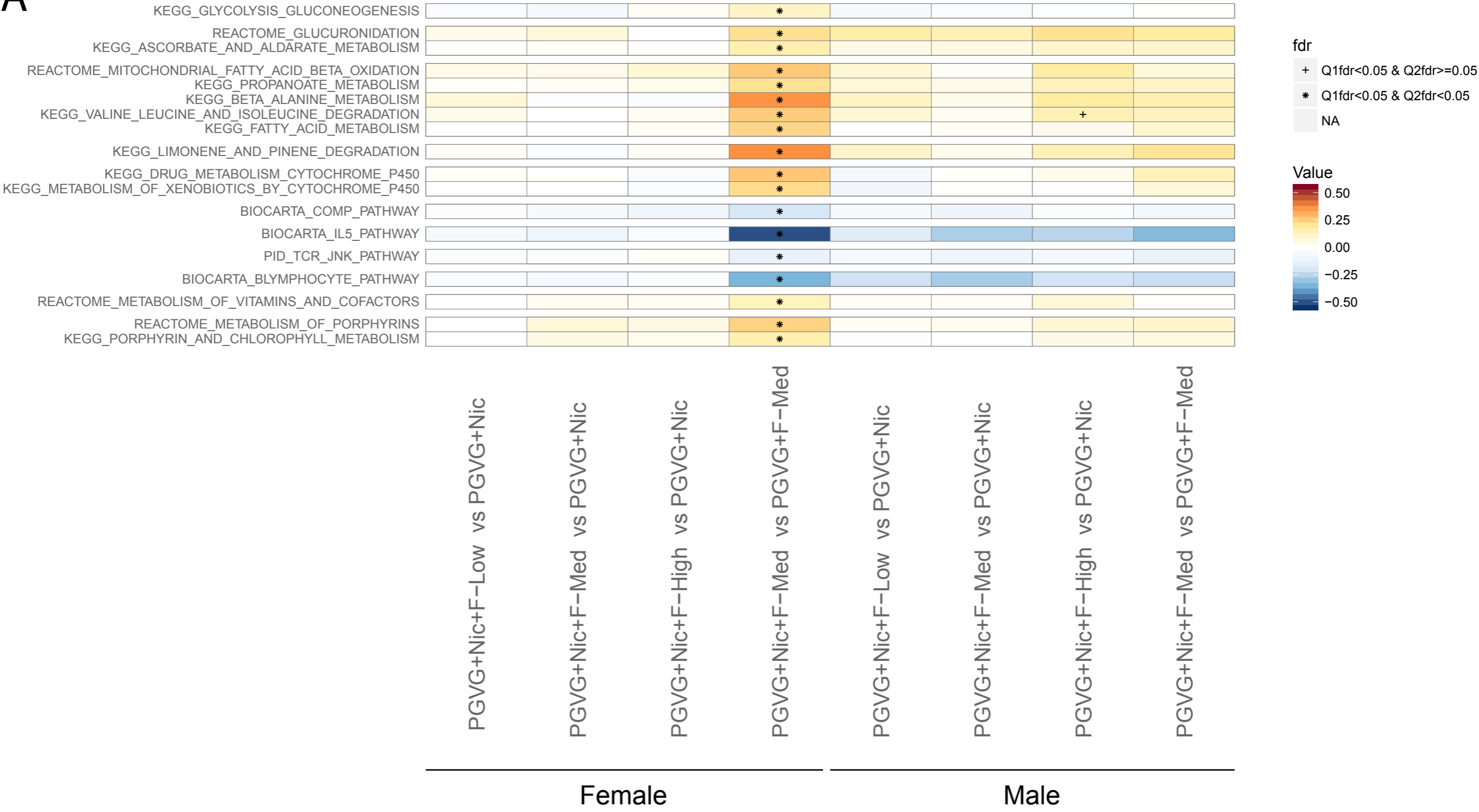

Supplement: Supplementary file 4 — Supplementary file4 (PDF 166 kb) [file 204_2020_2759_MOESM4_ESM.pdf]

B

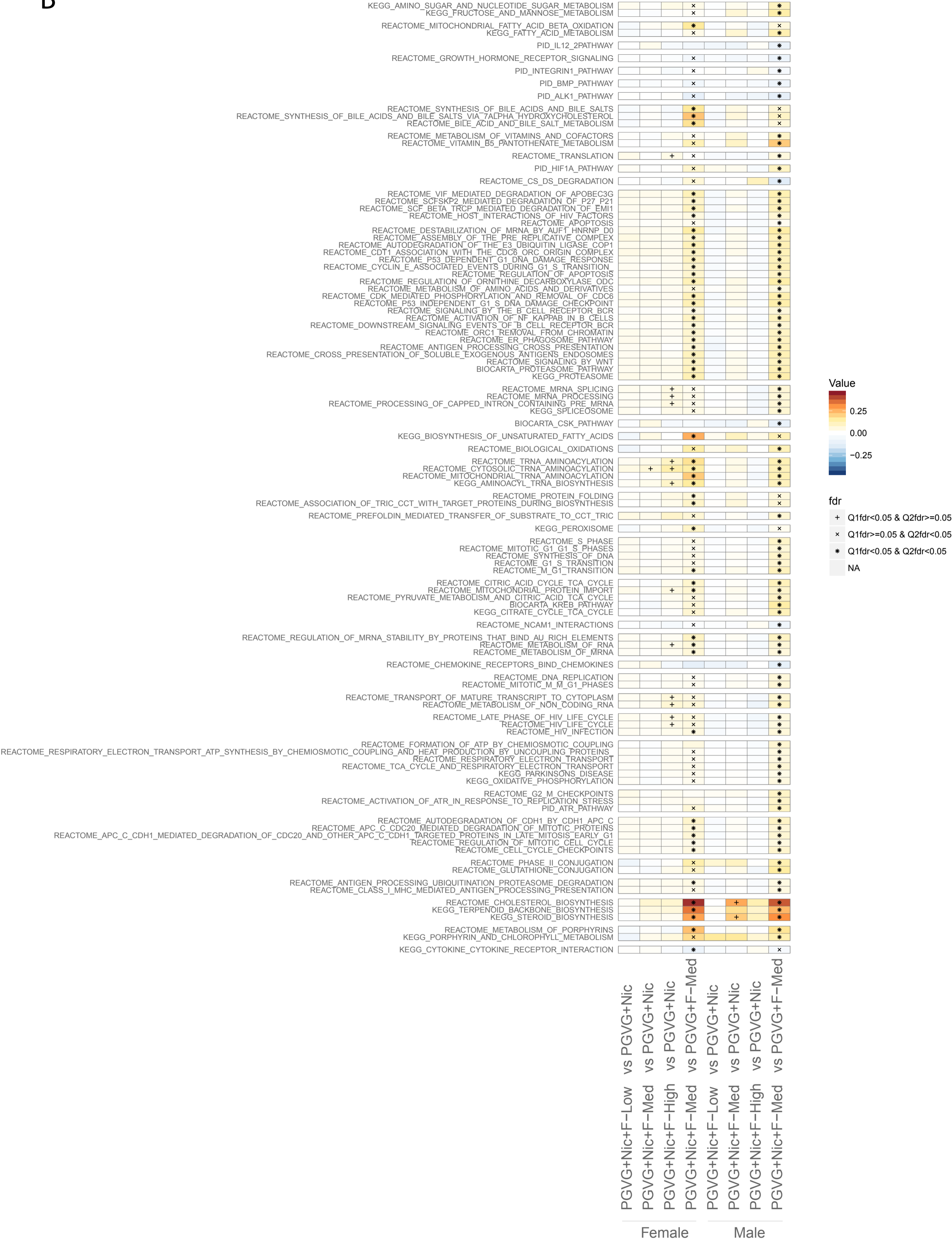

Supplement: Supplementary file 5 — Supplementary file5 (PDF 323 kb) [file 204_2020_2759_MOESM5_ESM.pdf]
