# Supplementary material for: Evaluation of toxicity of aerosols from flavored e-liquids in Sprague–Dawley rats in a 90-day OECD inhalation study, complemented by transcriptomics analysis
Source: Arch Toxicol. 2020 May 5;94(6):2179–206. doi: 10.1007/s00204-020-02759-6 (PMC7303093; doi:10.1007/s00204-020-02759-6)

Liver panel (Phillips et al. 2017)

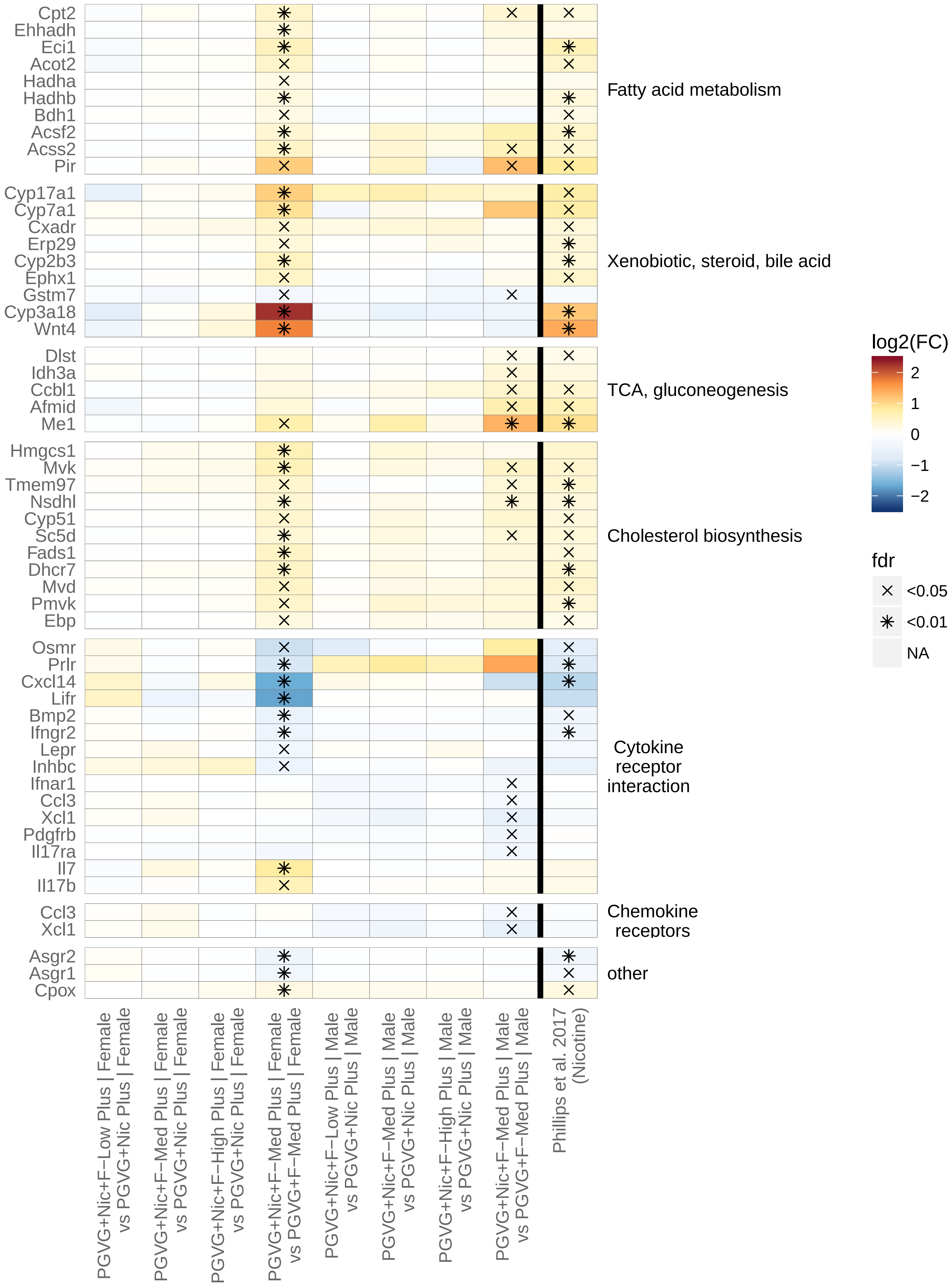

Supplement: Supplementary file 6 — Supplementary file6 (PDF 19 kb) [file 204_2020_2759_MOESM6_ESM.pdf]
