# Supplementary material for: Evaluation of toxicity of aerosols from flavored e-liquids in Sprague–Dawley rats in a 90-day OECD inhalation study, complemented by transcriptomics analysis
Source: Arch Toxicol. 2020 May 5;94(6):2179–206. doi: 10.1007/s00204-020-02759-6 (PMC7303093; doi:10.1007/s00204-020-02759-6)
Supplement: Supplementary file 7 — Supplementary file7 (DOCX 256 kb) [file 204_2020_2759_MOESM7_ESM.docx]

Evaluation of toxicity of aerosols from flavoured e-liquids in a 90-day OECD inhalation study, complemented by transcriptomics analysis

Jenny Ho^a^, Davide Sciuscio^b^, Ulrike Kogel^b^, Bjoern Titz^b^, Patrice Leroy^b^, Gregory Vuillaume^b^, Marja Talikka^b^, Elyette Martin^b^, Pavel Pospisil^b^, Stefan Lebrun^b^, Wenhao Xia^a^, Tom Lee^a^, Yun Xuan Chng^a^, Blaine W. Phillips^a^, Emilija Veljkovic^b^, Emmanuel Guedj^b^, Yang Xiang^b^, Nikolai V. Ivanov^b^, Manuel C. Peitsch^b^, Julia Hoeng^b^, Patrick Vanscheeuwijck^b*^

^a^ PMI S&I, Philip Morris International Research Laboratories Pte. Ltd., Science Park II, Singapore

^b^ PMI S&I, Philip Morris Products S.A., Quai Jeanrenaud 5, CH-2000 Neuchâtel, Switzerland

***Corresponding author**
E-mail: [Patrick.Vanscheeuwijck@pmi.com](mailto:Patrick.Vanscheeuwijck@pmi.com)

Tel: +41 (58) 242 2511

# Supplemental Material

## **Animals and treatment**

The air supplied to the animal rooms was filtered fresh air, and positive pressure was maintained inside the animal rooms. The room temperature was kept at 22 ± 2°C, and the relative humidity remained within the targeted 30–70%. The light/dark cycle was 12 hours/12 hours, with the light period starting at 07:00. Identification, housing, feeding, and watering were performed as previously described ([Phillips et al. 2015](#_ENREF_7)). Two rats of the same sex were housed together per cage; enrichment (e.g., gnawing bones and tunnels) was provided. Autoclaved softwood (spruce and fir) granulate (Lignocel^®^ BK 8-15; Rettenmaier & Soehne, Rosenberg, Germany) was used as bedding, and gamma-irradiated pellet diet (2914C irradiated rodent diet; Envigo, USA) and sterilized drinking water were provided *ad libitum*, except during exposure. Rats were allowed to acclimatize to their environment for 10 days before exposure to test aerosols.

## **Analytical characterization of test atmosphere and biomonitoring**

TPM, PG, and VG were collected using Cambridge filters, while nicotine was trapped on sulfuric acid-impregnated diatomaceous earth (EXtrelut^®^ NT3, Merck Millipore, USA). The samples were analyzed according to previously described methods ([Phillips et al. 2017](#_ENREF_8)). L-carvone in the test atmosphere was separated by capillary gas chromatography (GC) on a 30 m x 0.25 mm internal diameter, 0.5 µm film thickness DB-WAXetr (Part number: 122-7333, Agilent J&W, USA) column and detected using a flame ionization detector after trapping on 2-propanol-impregnated diatomaceous earth (EXtrelut^®^ NT3, Merck Millipore, USA). The column was equilibrated at 110°C for one minute before being increased to 130°C over 5 minutes and held for 3 minutes. The temperature was ramped to 150°C in 2 minutes (hold for 5 minutes) and to 240°C at 80°C/minute before being held for another 5 minutes. For details of the frequency and methods of determination for aerosol parameter, see **Supplemental Table 4** for details.

Urinary nicotine metabolites (24-hour urine samples, including the 6-hour exposure period and an 18-hour post-exposure period) were measured by high-performance liquid chromatography (HPLC) following derivatization with 1,3-diethyl-2-thiobarbituric acid. The HPLC system (Agilent, Santa Clara, United States) consisted of a 1200 Infinity LC system attached with quaternary pump, Diode Array HPLC Detector, CTC PAL automated sample injectors, PAL Stack Cooler 6 DW, and OpenLAB CDS ChemStation. Chromatographic separation was achieved with a Phenomenex Synergi Max-RP (150 mm x 2.0 mm internal diameter, 4 µm particle diameter) fitted with a guard column (Phenomenex Security Guard Assembly and Cartridge C12, 4 mm x 3.0 mm internal diameter, 5 µm particle diameter). The column oven was maintained at 36°C. Separation of the analytes of interest was obtained using gradient elution with mobile phase A (aqueous solution of 9.5 g/L 1-pentanesulfonic acid, 2.7 g/L 1-heptanesulfonic acid, 0.6 g/L 1-octanesulfonic acid, and 0.15 g/L ammonium acetate adjusted to pH 4.72 with 10% acetic acid and conductivity 4.7–4.9 mS/cm), mobile phase B (0.45 g/L ammonium acetate in methanol), and mobile phase C (96/4 v/v of acetonitrile/tetrahydrofurane) at a flow rate of 0.62 mL/minute. The initial condition of 68% A, 22% B, and 10 % C was changed to 68% A, 5% B, and 27% C at 4 minutes. The gradient was changed to 55% A and 45% C at 9 minutes and 20% A and 80% C at 12 minutes. Subsequently, the 100% C was maintained from 12.2 minutes to 13 minutes before being gradually changed to 76% A and 24% B at 14 minutes and back to initial condition at 14.5 minutes. Blood samples were collected from all rats (OECD and OECD Plus groups) from the saphenous vein under isoflurane anesthesia within 20 minutes after removal from the exposure chamber and prepared into plasma. The nicotine and cotinine in plasma were extracted (liquid-liquid) with binary solvents and derivatized with 1,3-diethyl-2-thiobarbituric acid before separation using the aforementioned HPLC method. Plasma PG, L-carvone, linalool, and citronellol were determined using LC coupled to tandem mass spectrometry or headspace solid-phase microextraction coupled to GC–mass spectrometry methods (Analytisch-Biologisches Forschungslabor GmbH, Munich, Germany).

## **Biological parameters**

In brief, respiratory physiology was measured once in the study using head-out plethysmography (EMKA Technologies, Paris, France) for the 10 male and 10 female rats in OECD groups to assess breathing frequency, tidal volume, peak inspiratory flow, and respiratory minute volume. Food consumption was determined weekly by group and sex, with values normalized to body weight and presented in g/(day x 100 g body weight).

All rats in the OECD groups were euthanized after approximately 13 weeks of exposure (16–24 hours after the final exposure) according to OECD TG 413. The rats were not fasted before the terminal procedures. For blood full differential analysis, blood samples were collected from the retro-orbital venous plexus under pentobarbital anesthesia and analyzed using a Sysmex XT-2000i hematology analyzer (Sysmex Corporation, Kobe, Japan). For blood clinical chemistry analysis, serum was collected from the abdominal aorta, and evaluation was performed using a UniCel^®^ DxC 600i clinical analyzer system (Beckman Coulter, USA). Aliquots of blood were processed into plasma using citrated tubes, and the clotting potential parameters were determined using the STA Compact Max® coagulation analyzer (Diagnostic Stago Inc., USA).

BALF was collected from the right lung during necropsy using 5 consecutive cycles (cycle 1, PBS; cycles 2-5, PBS + 0.325% w/v bovine serum albumin) of instillation at 15 cm H_2_O pressure. The cell pellets from cycle 1 to 5 were combined, and the cellular contents of free lung cells were determined using flow cytometry as previously described ([Wong et al. 2016](#_ENREF_10)). Cell-free BALF from cycle 1 (remaining after centrifugation of cells for BALF) was frozen and used for multi-analyte profiling using Luminex xMAP® technology (Merck Millipore, USA) in a 96-well microtiter plate (GLP compliance was not claimed for this specific analysis).

## **Pathology**

The organs listed in OECD TG 413 (see **Supplemental Table 6**) were harvested, and the weights of the spleen, thymus, lung with larynx and trachea, heart, kidneys, adrenal glands, gland thyroid and parathyroid, testes, epididymis, ovaries, uterus, brain, and liver were determined. Organs were weighed in pairs for bilateral organs. Both respiratory tract and non-respiratory tract organs were fixed in 4% formaldehyde solution (4% FA), except for the sternum, testes, and eyes, which were fixed in Schaffer’s, Bouin’s, and Davidson’s solutions, respectively. After 24 hours in Davidson’s solution, eyes were transferred to and stored in 70% ethanol; after 48 hours in Bouin’s solution, testes were transferred to 4% FA. Histological sections of respiratory tract organs were prepared at test facility for defined levels and stained with hematoxylin and eosin. The nasal cavity level 1, tracheal bifurcation, and lung with mainstem bronchus were also stained with Alcian blue/periodic acid Schiff’s stain to detect the mucus-secreting cells in the tissue, especially goblet cells in the lung tissue. The histological slides of non-respiratory organs were prepared by Laboratory of Pharmacology and Toxicology GmbH (Hamburg, Germany).

Incidence of histopathologic findings was recorded, and the severity of the lesions was evaluated using a scoring system of 0 to 5 in a semi-blinded manner (i.e., the rats were grouped into their respective treatment groups, but only the identity of the PBS control and PG/VG + Nic-exposed groups were revealed) with the following definitions:

- GRADE 0 = Normal
- GRADE 1 = Minimal
- GRADE 2 = Mild/slight
- GRADE 3 = Moderate
- GRADE 4 = Marked
- GRADE 5 = Severe

Morphometric analysis of the laryngeal epithelial thickness of OECD groups was performed on sections from the floor of the larynx and the lower medial region of vocal cords (level of arytenoid projections). Bone marrow was collected by using brush dip with a solution (prepared from fetal bovine serum and 7.5% w/v ethylenediaminetetraacetic acid in a ratio of 2:1) to obtain some marrow from the exposed cavity of the right femur of rats, applied on glass slides, and fixed in methanol. The bone marrow smears were subsequently stained according to the Pappenheim method and evaluated by Laboratory of Pharmacology and Toxicology GmbH (Hamburg, Germany) by counting 200 nucleus-containing cells.

## **Transcriptomics analysis (“Plus” part)**

Prior to organ removal, a whole-body perfusion with ice cold [saline](https://www.sciencedirect.com/topics/pharmacology-toxicology-and-pharmaceutical-science/sodium-chloride) was performed to remove all blood from the organs by passage of the fluid through blood vessels. The saline was perfused with peristaltic pump and 20G needle inserted into the heart left ventricle of an anesthetized rat. An ‘exit cut’ was made at right atrium of heart and the organs of interest (e.g. liver and lungs) were massaged for approximately 8 minutes until all organs appeared pale, the needle was re-inserted into the right ventricle to further perfuse the lungs for approximately 1 minute before organ collection. Resected organs were stored at ≤−70°C. The left lung lobe was cryosectioned into 40 μm slices, and the slices were collected alternately for transcriptomics and backups. RNA was isolated from the lung slices, from the respiratory nasal [epithelium](https://www.sciencedirect.com/topics/earth-and-planetary-sciences/epithelium) (RNE) that was taken from the left side of the nose, and from a 5x5x5 mm piece of the liver with the miRNeasy extraction kit (Qiagen, Hilden, Germany). RNA was processed for hybridization on GeneChip^®^ Rat Genome 230 2.0 arrays (Affymetrix, Santa Clara, California, USA) using a High-Throughput 3′ In Vitro Transcription PLUS kit (Affymetrix).

Raw data files were processed in the custom Chip Description File environment Rat2302_Rn_ENTREZG v19.0.0 (rat2302rnentrezgcdf) ([Dai et al. 2005](#_ENREF_3)) and normalized using frozen robust microarray analysis ([McCall et al. 2010](#_ENREF_6)). Quality controls, including log-intensities, normalized unscaled standard error, relative log expression (RLE), median absolute value RLE, and pseudo-images as well as raw image plots, were performed with the affyPLM package (Bioconductor suite) ([Bolstad et al. 2013](#_ENREF_2)). After quality control, the nasal tissue, female non-flavored reference group (PG/VG + Nic) had only 4 biological replicates left. All other groups had at least 5 and up to 8 biological replicates. Raw *p*-values were generated for pairwise group comparisons with the limma package ([Smyth 2004](#_ENREF_9)) and adjusted using the Benjamini-Hochberg false discovery rate (FDR) multiple test correction ([Gentleman et al. 2004](#_ENREF_4)). The pairwise comparisons that were used to extract information on the flavor effect on the transcriptome were flavored e-liquids (PG/VG + Nic + F-Low, PG/VG + Nic + F-Med, PG/VG + Nic + F-High) versus non-flavored reference (PG/VG + Nic). The pairwise comparison that was used to extract information on the nicotine effect on the transcriptome was PG/VG + Nic + F-Med versus no-nicotine reference (PG/VG + F-Med).

Gene sets were obtained from the Molecular Signatures Database (mSigDB) ([Liberzon et al. 2015](#_ENREF_5)). Significance of the gene-set enrichment was assessed using a competitive null hypothesis (Q1) and a self-contained null hypothesis (Q2) ([Ackermann and Strimmer 2009](#_ENREF_1)). Q1 tests for the significance of genes in the set versus those not in the set, and Q2 tests for a significant difference between the conditions. With this, Q2 represents the primary assessment of significant exposure effects, while Q1 can be used for gene-set prioritization, highlighting gene-sets that dominate the significant exposure effects. Q1 statistics were calculated with the Camera approach, and the Q2 statistics were calculated with the Roast approach, which both take the gene correlation structures into account ([Wu et al. 2010](#_ENREF_11); [Wu and Smyth 2012](#_ENREF_12)). The resulting *p*-values were adjusted for multiple hypothesis testing using the Benjamini–Hochberg procedure.

## **Statistical evaluation**

Unless otherwise stated, statistical analysis of continuous parameter values was conducted by computing basic descriptive statistics (N, mean, and standard deviation (SD) or standard error of mean (SEM), median, minimum, and maximum); if the parameter was ordinal, N and a frequency table (absolute and relative to N) were computed as well as mean and SD or SEM. For incidence parameters, N and a frequency table (absolute and relative to N) were computed.

For comparative statistics, pairwise differences between groups described in **Supplemental Table 6** were calculated separately for each sex. For continuous variables, this was performed using *t*-tests accounting for variance heterogeneity (Satterthwaite method). If both groups exhibited strong deviation from the normality assumption (as assessed by the Shapiro-Wilk test at 5% applied on the Pearson residuals of both groups being compared), a non-parametric Mann–Whitney–Wilcoxon rank sum test was performed. For incidences, this was done using Fisher’s exact test. For ordinal variables, this was done by Mann–Whitney–Wilcoxon rank sum tests.

**Supplemental Table 1 Flavouring substances considered in study**

See attached score sheet pdf).

**Supplemental Table 2 Chemical groups defined in the Commission Regulation (EC) No 1565/2000**

| **Group** | **Description** |
| --- | --- |
| Group 1 | Straight-chain primary aliphatic alcohols/aldehydes/acids, acetals and esters with esters containing saturated alcohols and acetals containing saturated aldehydes. No aromatic or heteroaromatic moiety as a component of an ester or acetal. |
| Group 2 | Branched-chain primary aliphatic alcohols/aldehydes/acids, acetal and esters with esters containing branched-chain alcohols and acetals containing branched-chain aldehydes. No aromatic or heteroaromatic moiety as a component of an ester or acetal. |
| Group 3 | α, β-unsaturated (alkene or alkyne) straight-chain and branched-chain aliphatic primary alcohols/aldehydes/acids, acetals and esters with esters containing α, β-unsaturated alcohol and acetal containing α, β-unsaturated alcohols or aldehydes. No aromatic or heteroaromatic moiety as a component of an ester or acetal. |
| Group 4 | Non-conjugated and accumulated unsaturated straight-chain and branched-chain aliphatic primary alcohols/aldehydes/acids, acetals and esters with esters containing unsaturated alcohols and acetals containing unsaturaed alcohols or aldehydes. No aromatic or heteroaromatic moiety as a component of an ester or acetal. |
| Group 5 | Saturated and unsaturated aliphatic secondary alcohols/ketones/ketals/esters with esters containing secondary alcohols. No aromatic or heteroaromatic moiety as a component of an ester or ketal. |
| Group 6 | Aliphatic, alicyclic and aromatic saturated and unsaturated tertiary alcohols and esters with esters containing tertiary alcohols. Esters may contain any acid component. |
| Group 7 | Primary alicyclic saturated and unsaturated alcohols/aldehydes/acids/acetals/esters with esters containing alicyclic alcohols. Esters/acetals may contain aliphatic acyclic or alicylic acids or alcohol component. |
| Group 8 | Secondary alicyclic saturated and unsaturated alcohols/ketones/ketals/esters with ketals containing alicyclic alcohols or ketones and esters containing secondary alicyclic alcohols. Esters may contain aliphatic acyclic or alicyclic acid component. |
| Group 9 | Primary aliphatic saturated or unsaturated alcohols/aldehydes/acids/acetals/esters with a second primary, secondary or tertiary oxygenated functional group including aliphatic lactones. |
| Group 10 | Secondary aliphatic saturated or unsaturated alcohols/ketones/ketals/esters with a second secondary or tertiary oxygenated functional group. |
| Group 11 | Alicyclic and aromatic lactones. |
| Group 12 | Maltol derivatives and ketodioxane derivatives. |
| Group 13 | Furanones and tetrahydrofurfuryl derivatives. |
| Group 14 | Furfuryl and furan derivatives with and without additional side-chain substituents and heteroatoms. |
| Group 15 | Phenyl ethyl alcohols, phenylacetic acids, related esters, phenoxyacetic acids and related esters. |
| Group 16 | Aliphatic and alicyclic ethers. |
| Group 17 | Propenylhydroxybenzenes. |
| Group 18 | Allylhydroxybenzenes. |
| Group 19 | Capsaicin related substances and related amides. |
| Group 20 | Aliphatic and aromatic mono- and di- thiols and mono-, di-, tri-, and polysulfides with or without additional oxygenated functional groups. |
| Group 21 | Aromatic ketones, secondary alcohols and related esters. |
| Group 22 | Aryl-substituted primary alcohol/aldehyde/acid/ester/acetal derivatives, including unsaturated ones. |
| Group 23 | Benzyl alcohols/aldehydes/acids/esters/acetals. Benzyl and benzoate esters included. May also contain aliphatic acyclic or alicyclic ester or acetal component. |
| Group 24 | Pyrazine derivatives. |
| Group 25 | Phenol derivatives containing ring-alkyl, ring-alkoxy, and side-chains with an oxygenated functional group. |
| Group 26 | Aromatic ethers including anisole derivatives. |
| Group 27 | Anthranilate derivatives. |
| Group 28 | Pyridine, pyrrole, and quinoline derivatives. |
| Group 29 | Thiazoles, thiophene, thiazoline and thienyl derivatives. |
| Group 30 | Miscellaneous substances. |
| Group 31 | Aliphatic and aromatic hydrocarbons. |
| Group 32 | Epoxides. |
| Group 33 | Aliphatic and aromatic amines. |
| Group 34 | Amino acids. |

**Supplemental Table 3** **Overview of composition per one liter of stock solution of test and reference items**

| **Compound** | **PG/VG + Nic (g)** | **PG/VG + F-Med (g)** | **PG/VG + Nic + F-Low (g)** | **PG/VG + Nic + F-Med (g)** | **PG/VG + Nic + F-High (g)** |
| --- | --- | --- | --- | --- | --- |
| Nicotine | 6.06 | NA | 6.06 | 6.06 | 6.06 |
| Propylene glycol | 426.40 | 426.40 | 426.40 | 426.40 | 426.40 |
| Vegetable glycerin | 475.00 | 475.00 | 475.00 | 475.00 | 475.00 |
| Ethyl acetate | NA | 5.040 | 0.389 | 5.040 | 7.560 |
| Butyric acid | NA | 5.376 | 0.415 | 5.376 | 8.064 |
| Ethyl formate | NA | 5.152 | 0.397 | 5.152 | 7.728 |
| Isobutyl alcohol | NA | 0.435 | 0.346 | 0.435 | 0.653 |
| Allyl hexanoate | NA | 0.249 | 0.128 | 0.249 | 0.374 |
| D,L-Citronellol | NA | 4.128 | 0.310 | 4.128 | 6.192 |
| 2-Heptanone | NA | 0.268 | 0.134 | 0.268 | 0.401 |
| Linalool | NA | 2.088 | 1.044 | 2.088 | 3.132 |
| L-Menthone | NA | 1.068 | 0.447 | 1.068 | 1.602 |
| L-Carvone | NA | 1.152 | 0.483 | 1.152 | 1.728 |
| *gamma*-Valerolactone | NA | 3.150 | 0.454 | 3.150 | 4.725 |
| 3-Methyl-2,4-nonanedione | NA | 0.019 | 0.010 | 0.019 | 0.029 |
| Ethyl maltol | NA | 10.282 | 5.141 | 10.282 | 15.422 |
| Furaneol | NA | 1.386 | 0.693 | 1.386 | 2.079 |
| Phenethyl alcohol | NA | 3.917 | 1.958 | 3.917 | 5.875 |
| Eucalyptol | NA | 0.662 | 0.099 | 0.662 | 0.994 |
| Eugenyl acetate | NA | 1.555 | 0.778 | 1.555 | 2.333 |
| 3-(Methylthio)propionaldehyde | NA | 0.092 | 0.00005 | 0.092 | 0.137 |
| 4-(*p*-Hydroxyphenyl)butan-2-one | NA | 5.232 | 2.616 | 5.232 | 7.848 |
| Cinnamyl alcohol | NA | 0.524 | 0.075 | 0.524 | 0.786 |
| Methyl salicylate | NA | 2.714 | 0.084 | 2.714 | 4.072 |
| 2-Ethyl-3,5-dimethylpyrazine | NA | 0.155 | 0.00065 | 0.155 | 0.233 |
| Guaiacol | NA | 0.121 | 0.108 | 0.121 | 0.182 |
| Methyl anthranilate | NA | 0.337 | 0.084 | 0.337 | 0.505 |
| 2-Acetylpyridine | NA | 0.907 | 0.454 | 0.907 | 1.361 |
| 2-Acetylthiazole | NA | 0.020 | 0.010 | 0.020 | 0.030 |
| Methyl cyclopentenolone | NA | 2.160 | 0.648 | 2.160 | 3.240 |
| *alpha*-Pinene | NA | 0.413 | 0.077 | 0.413 | 0.619 |

**Supplemental Table 4 Test atmosphere characterization, frequency, and methods of determination**

| **Parameter** | **Assay principle** | **Determination schedule for PBS control chamber** | **Determination schedule for non-flavoured e-liquid chamber** | **Determination schedule for flavours in PG/VG only chamber** | **Determination schedule for flavoured e-liquid chamber** |
| --- | --- | --- | --- | --- | --- |
| TPM | Gravimetry after trapping on Cambridge filters (weighing together with holder) ^a^ | ≥4 times ^b^ per 6 h exposure; 0.5 h per sampling | ≥4 times ^b^ per 6 h exposure; 0.5 h per sampling | ≥4 times ^b^ per 6 h exposure; 0.5 h per sampling | ≥4 times ^b^ per 6 h exposure; 0.5 h per sampling |
| Nicotine | Capillary GC after trapping on sulfuric acid-impregnated diatomaceous earth*^* | ≥1 time per week; 0.5 h per sampling | ≥4 times ^b^ per 6 h exposure; 0.5 h per sampling | ≥1 time per week; 0.5 h per sampling | ≥4 times ^b^ per 6 h exposure; 0.5 h per sampling |
| PG/VG | Capillary GC after trapping on Cambridge filters ^a^ | Not measured | ≥1 time per 6 h exposure; 0.5 h per sampling | ≥1 time per 6 h exposure; 0.5 h per sampling | ≥1 time per 6 h exposure; 0.5 h per sampling |
| L-Carvone | Capillary GC after trapping on 2-propanol-impregnated diatomaceous earth | Not measured | ≥1 time per week; 0.5 h per sampling | ≥1 time per 6 h exposure; 0.5 h per sampling | ≥1 time per 6 h exposure; 0.5 h per sampling |
| Temperature | Thermistor probe Pt100 (located at chamber inlet) | Continuously | Continuously | Continuously | Continuously |
| Dilution flow rate | Pressure difference over Venturi tube | Continuously | Continuously | Continuously | Continuously |
| Compressed air flow rate | Mass flow meter | Continuously | Continuously | Continuously | Continuously |
| Relative humidity | Capacitive measurement | ≥1 time per day (at chamber inlet); intermittent check ^c^ | Not measured ^d^ | Not measured ^d^ | Not measured ^d^ |
| Conductivity | Ion concentration after trapping aerosol in 20 mL deionized water (using impinger) ^e^ | ≥1 time per week; 0.5h per sampling ^c^ |  |  |  |
| Particle size distribution | Aerodynamic particle sizer (spectrophotometric) | ≥1 time per week | ≥1 time per week | ≥1 time per week | ≥1 time per week |

*Remark:*

^a^ *The Cambridge filters were weighed together with the holder to determine TPM.*

^b^ *Sampling during week 1 was performed ≥1 time per day due to time adaptation schedule.*

^c^ *The results were for reference purposes and GLP compliance was not claimed*

^d^ *No measurement of relative humidity was performed in the exposure chambers because the aerosols can potentially interfere with the humidity sensor for measurement.*

^e^ *The ion concentration of deionized water was measured before and after the trapping.*

*Abbreviations: h, hours.*

**Supplemental Table 5** **In-life observations, frequency, and methods of determination**

| **Parameter** | **Method** | **Frequency** | **Number of Rats** | **Remarks** |
| --- | --- | --- | --- | --- |
| Body weight | Gravimetry | ≥ 2 times per week during exposure | All rats | Individually |
| Food consumption | Gravimetry | 1 time per week | All rats in OECD and OECD Recovery groups | Determined per group per sex |
| Ophthalmoscopy | Examination using an indirect and direct ophthalmoscope | Once before 90-d exposure period | All rats in OECD and OECD Recovery groups | Individually |
|  |  | Once towards the end of 90-d exposure period | All rats in OECD and OECD Recovery groups | Individually Groups 18 – 24 were examined |
|  |  | Once towards the end of the recovery period | All rats in OECD Recovery groups | Individually |
| In-life observations and special observations | Individual observations according to checklist in Preclinical Toxicology Software (Provantis^®^) | Daily | Each rat systematically observed ≥ 4 times within the 13-week exposure period | Within 30 minutes after removal from exposure tube. |
|  | Group observations according to checklist in Provantis^®^ | Daily | All rats ^a^ | Within 30 minutes after removal from exposure tube. |
| Mortality | Observation | Daily | All rats | Within 30 minutes after removal from exposure tube. |

^a^ *Except animals scheduled for urine or blood collection on the day.*

**Supplemental Table 6** **List of pairwise comparisons for OECD groups**

| Control | Test |
| --- | --- |
| PBS | PG/VG + Nic |
| PBS | PG/VG + F-Med |
| PBS | PG/VG + Nic + F-Low |
| PBS | PG/VG + Nic + F-Med |
| PBS | PG/VG + Nic + F-High |
| PG/VG + Nic | PG/VG + F-Med |
| PG/VG + Nic | PG/VG + Nic + F-Low |
| PG/VG + Nic | PG/VG + Nic + F-Med |
| PG/VG + Nic | PG/VG + Nic + F-High |
| PG/VG + F-Med | PG/VG + Nic + F-Med |
| PG/VG + Nic + F-Low | PG/VG + Nic + F-Med |
| PG/VG + Nic + F-Low | PG/VG + Nic + F-High |
| PG/VG + Nic + F-Med | PG/VG + Nic + F-High |
| PG/VG + Nic (R) | PG/VG + Nic |
| PG/VG + Nic (R) | PG/VG + Nic + F-High (R) |
| PG/VG + Nic + F-High (R) | PG/VG + Nic + F-High |

**Supplemental Table 7** **Organs collected for histopathological examination and organ weight determination**

| **Male** | **Female** | **Weigh** | **Fixatives** | **Remark** |
| --- | --- | --- | --- | --- |
| Lung, larynx and trachea | Lung, larynx and trachea | Yes | 4% formaldehyde | Weighed as one under ‘Lung’ |
| Nose: 4 transverse sections: - Nasal cavity level 1: one immediately posterior to the upper incisor teeth - Nasal cavity level 2: one posterior to incisive papilla - Nasal cavity level 3: one at second palatal ridge - Nasal cavity level 4: one between first and second molar teeth | Nose: 4 transverse sections: - Nasal cavity level 1: one immediately posterior to the upper incisor teeth - Nasal cavity level 2: one posterior to incisive papilla - Nasal cavity level 3: one at second palatal ridge - Nasal cavity level 4: one between first and second molar teeth |  | 4% formaldehyde |  |
| Larynx: 4 transverse sections: - Mid-base of epiglottis - Distal base of epiglottis - Arytenoid projections - Vocal folds | Larynx: 4 transverse sections: - Mid-base of epiglottis - Distal base of epiglottis - Arytenoid projections - Vocal folds |  | 4% formaldehyde |  |
| Trachea:  - Longitudinal section at bifurcation - Transverse section | Trachea:  - Longitudinal section at bifurcation - Transverse section |  | 4% formaldehyde |  |
| Lung - 3 longitudinal sections of left lobe:  first at main bronchus, then 2 sections 1000 µm apart | Lung - 3 longitudinal sections of left lobe:  first at main bronchus, then 2 sections 1000 µm apart |  | 4% formaldehyde |  |
| Gland, adrenal (left, right) | Gland, adrenal (left, right) | Yes ^a^ | 4% formaldehyde | Paired examination ^b^ |
| Aorta | Aorta | NA | 4% formaldehyde | NA |
| Femur and stifle joint (left) | Femur and stifle joint (left) | NA | 4% formaldehyde | Collected distal end to include femoral tibial joint (stifle joint), decalcified before sectioning |
| Brain | Brain | Yes | 4% formaldehyde | Included sections of cerebrum, cerebellum, medulla/pons |
| Caecum | Caecum | NA | 4% formaldehyde | NA |
| Colon | Colon | NA | 4% formaldehyde | NA |
| Epididymis (left, right) |  | Yes ^a^ | 4% formaldehyde | Paired examination ^b^ |
| Eye (left, right) | Eye (left, right) | NA | Davidson's fixative ^c^ | Paired examination ^b^, the optic nerve, retina, eyelids and Harderian glands (bilateral) were collected with eyes. |
| Optic nerve (left, right) | Optic nerve (left, right) | NA | Davidson's fixative ^c^ | Paired examination ^b^, collected with eyes |
| Gland, Harderian (left, right) | Gland, Harderian (left, right) | NA | Davidson's fixative ^c^ | Paired examination ^b^, collected with eyes |
| Heart | Heart | Yes | 4% formaldehyde | NA |
| Ileum | Ileum | NA | 4% formaldehyde | NA |
| Jejunum | Jejunum | NA | 4% formaldehyde | NA |
| Kidney (left, right) | Kidney (left, right) | Yes ^a^ | 4% formaldehyde | Paired examination ^b^ |
| Gland, lacrimal (left, right) | Gland, lacrimal (left, right) | NA | 4% formaldehyde | Extraorbital; paired examination |
| Liver | Liver | Yes | 4% formaldehyde | NA |
| Lymph node, bronchial | Lymph node, bronchial | NA | 4% formaldehyde | NA |
| Lymph node, mediastinal | Lymph node, mediastinal | NA | 4% formaldehyde | NA |
| Lymph node, mesenteric | Lymph node, mesenteric | NA | 4% formaldehyde | NA |
| Esophagus | Esophagus | NA | 4% formaldehyde | NA |
|  | Ovary (left, right) | Yes ^a^ | 4% formaldehyde | Paired examination ^b^ |
| Pancreas | Pancreas | NA | 4% formaldehyde | NA |
| Gland, pituitary | Gland, pituitary | NA | 4% formaldehyde | NA |
| Gland, prostate |  | NA | 4% formaldehyde | NA |
| Rectum | Rectum | NA | 4% formaldehyde | NA |
| Gland, salivary | Gland, salivary | NA | 4% formaldehyde | Paired examination ^b^, Parotid, mandibular, sublingual |
| Gland, seminal |  | NA | 4% formaldehyde | Paired examination ^b^ |
| Skeletal muscle | Skeletal muscle | NA | 4% formaldehyde | Collected with sciatic nerve |
| Sciatic nerve | Sciatic nerve | NA | 4% formaldehyde | Collected with skeletal muscle |
| Skin | Skin | NA | 4% formaldehyde | NA |
| Gland, mammary | Gland, mammary | NA | 4% formaldehyde | Collected with skin |
| Spinal cord (cervical, thoracic, lumbar) | Spinal cord (cervical, thoracic, lumbar) | NA | 4% formaldehyde | NA |
| Spleen | Spleen | Yes | 4% formaldehyde | NA |
| Sternum | Sternum | NA | Schaffer's fixative | NA |
| Stomach | Stomach | NA | 4% formaldehyde | NA |
| Testis (left, right) |  | Yes ^a^ | Bouin's fluid ^d^ | Paired examination ^b^ |
| Thymus | Thymus | Yes | 4% formaldehyde | NA |
| Gland, thyroid | Gland, thyroid | Yes ^a^ | 4% formaldehyde | Collected and weighed with parathyroid; paired examination ^b^ |
| Gland, parathyroid | Gland, parathyroid | Yes ^a^ | 4% formaldehyde | Collected and weighed with thyroid; paired examination ^b^ |
| Urinary bladder | Urinary bladder | NA | 4% formaldehyde | NA |
|  | Uterus | Yes | 4% formaldehyde | Collected with cervix |
| Duodenum | Duodenum | NA | 4% formaldehyde | NA |
| Bone marrow, femur (right) | Bone marrow, femur (right) | NA | Isolate BM, cytospin and fix | Bone marrow smear was prepared from right femur |

*Remark:*

^a^ *Weighed in pairs for bilateral organs*

^b^ *Bilateral organs were examined in pairs. The most severe lesion was recorded if both sides were affected by the same lesion, and unilateral lesion observed at one side was recorded as such with locator.*

^c^ *After 24 hours, tissue was transferred to 70% ethanol.*

^d^ *After 48 hours, tissue was transferred to 4% formaldehyde (i.e., 10% formalin, neutral phosphate buffer).*

**Supplemental Table 8 Respiratory physiology measurements during exposure**

| **Parameter** | **Sex** | **PBS** | **PG/VG + Nic** | **PG/VG + F-Med** | **PG/VG + Nic + F-Low** | **PG/VG + Nic + F-Med** | **PG/VG + Nic + F-High** |
| --- | --- | --- | --- | --- | --- | --- | --- |
| Peak inspiratory flow (mL/s) | M | 12.8 ± 1.27 (10) | 17.0 ± 1.28 (10)* | 12.3 ± 1.02 (10) | 15.5 ± 1.84 (10) | 15.8 ± 0.94 (10) | 14.4 ± 1.14 (10) |
|  | F | 11.7 ± 0.67 (10) | 11.3 ± 0.80 (10) | 9.2 ± 0.81 (10)* | 11.6 ± 0.44 (10) | 13.0 ± 0.61 (10) | 11.4 ± 0.70 (10) |
| Tidal volume  (mL) | M | 1.3 ± 0.12 (10) | 1.9 ± 0.10 (10)** | 1.5 ± 0.10 (10) | 1.6 ± 0.12 (10) | 1.9 ± 0.16 (10)* | 1.9 ± 0.13 (10)** |
|  | F | 1.4 ± 0.07 (10) | 1.4 ± 0.11 (10) | 1.4 ± 0.08 (10) | 1.5 ± 0.15 (10) | 1.8 ± 0.08 (10)*** | 1.8 ± 0.09 (10)*** |
| Minute volume (mL/min) | M | 220.8 ± 21.92 (10) | 297.1 ± 23.09 (10)* | 241.7 ± 21.43 (10) | 253.0 ± 26.59 (10) | 274.5 ± 19.64 (10) | 275.2 ± 16.03 (10) |
|  | F | 218.3 ± 16.24 (10) | 205.5 ± 15.87 (10) | 197.4 ± 19.06 (10) | 203.3 ± 17.13 (10) | 250.6 ± 11.89 (10) | 250.0 ± 17.83 (10) |
| Breathing frequency (min^-1^) | M | 165.6 ± 8.35 (10) | 154.0 ± 6.99 (10) | 164.0 ± 8.20 (10) | 158.1 ± 9.38 (10) | 152.8 ± 7.80 (10) | 146.4 ± 4.97 (10) |
|  | F | 159.1 ± 6.47 (10) | 148.1 ± 10.63 (10) | 147.3 ± 10.96 (10) | 140.8 ± 6.75 (10) | 139.2 ± 4.42 (10)* | 137.3 ± 7.16 (10)* |

*Remark:*

*Results represent mean ± SEM. The sample size is in parentheses.*

*The Exposed groups are compared against PBS during “Exposed,” while the Recovery groups are compared against the same treatment group from “Exposed.” Significance: *, p<0.05; **, p<0.01; ***, p<0.001.*

*Abbreviations: M, male; F, female; min, minutes*.

**Supplemental Table 9** **Biomarkers of exposure in blood and urine**

| **Type** | **Parameter** | **Sex** | **Exposed** | | | | | |  | **Recovery** | |
| --- | --- | --- | --- | --- | --- | --- | --- | --- | --- | --- | --- |
|  |  |  | **PBS** | **PG/VG + Nic** | **PG/VG + F-Med** | **PG/VG + Nic + F-Low** | **PG/VG + Nic + F-Med** | **PG/VG + Nic + F-High** |  | **PG/VG + Nic** | **PG/VG + Nic + F-High** |
| Nicotine metabolites in urine  (concentration) | Urine volume  (mL) | M | 11.9 ± 1.33 (18) | 12.0 ± 1.31 (18) | 9.9 ± 1.11 (18) | 13.7 ± 1.42 (18) | 11.4 ± 1.52 (18) | 10.7 ± 1.50 (18) |  | 12.8 ± 0.86 (8) | 12.0 ± 2.28 (8) |
|  |  | F | 8.8 ± 0.63 (18) | 12.1 ± 1.26 (18)* | 9.8 ± 0.99 (18) | 13.7 ± 1.49 (18)** | 13.9 ± 1.42 (18)** | 11.2 ± 1.08 (18) |  | 5.9 ± 0.86 (8)*** | 10.6 ± 1.58 (8) |
|  | Nicotine  (µmol/L) | M | 0.2 ± 0.05 (18) | 90.3 ± 17.84 (18)*** | 0.3 ± 0.06 (18) | 76.0 ± 7.16 (18)*** | 94.7 ± 11.97 (18)*** | 100.3 ± 24.38 (18)*** |  | 0.1 ± 0.01 (8)*** | 0.1 ± 0.03 (8)*** |
|  |  | F | 0.2 ± 0.03 (18) | 87.3 ± 11.15 (18)*** | 0.5 ± 0.13 (18) | 117.1 ± 22.28 (18)*** | 95.7 ± 12.11 (18)*** | 102.7 ± 12.63 (18)*** |  | 0.1 ± 0.03 (8)*** | 0.2 ± 0.03 (8)*** |
|  | Cotinine  (µmol/L) | M | 0.1 ± 0.02 (18) | 107.4 ± 10.41 (18)*** | 0.7 ± 0.18 (18) | 95.1 ± 9.87 (18)*** | 110.7 ± 10.19 (18)*** | 114.1 ± 12.58 (18)*** |  | 0.2 ± 0.01 (8)*** | 0.2 ± 0.03 (8)*** |
|  |  | F | 0.2 ± 0.03 (18) | 116.6 ± 10.01 (18)*** | 1.0 ± 0.30 (18) | 114.9 ± 16.78 (18)*** | 89.9 ± 9.06 (18)*** | 114.1 ± 14.05 (18)*** |  | 0.2 ± 0.04 (8)*** | 0.1 ± 0.04 (8)*** |
|  | Nicotine-N’-oxide  (µmol/L) | M | 0.5 ± 0.08 (18) | 125.2 ± 16.66 (18)*** | 0.5 ± 0.15 (18) | 105.3 ± 13.79 (18)*** | 132.5 ± 16.89 (18)*** | 133.4 ± 18.19 (18)*** |  | 0.6 ± 0.01 (8)*** | 0.6 ± 0.01 (8)*** |
|  |  | F | 0.5 ± 0.07 (18) | 60.6 ± 6.23 (18)*** | 0.3 ± 0.09 (18)** | 94.6 ± 20.96 (18)*** | 67.5 ± 6.95 (18)*** | 88.7 ± 13.10 (18)*** |  | 0.2 ± 0.07 (8)*** | 0.5 ± 0.02 (8)*** |
|  | Norcotinine  (µmol/) | M | 0.2 ± 0.05 (18) | 50.9 ± 6.46 (18)*** | 0.6 ± 0.16 (18) | 45.4 ± 5.50 (18)*** | 50.1 ± 5.57 (18)*** | 59.1 ± 7.12(18)*** |  | 0.3 ± 0.08 (8)*** | 0.3 ± 0.06 (8)*** |
|  |  | F | 0.3 ± 0.07 (18) | 37.2 ± 5.35 (18)*** | 0.8 ± 0.16 (18)* | 38.8 ± 6.13 (18)*** | 31.8 ± 3.43 (18)*** | 39.7 ± 6.47(18)*** |  | 0.6 ± 0.06 (8)*** | 0.0 ± 0.04 (8)*** |
|  | Nornicotine  (µmol/L) | M | 0.1 ± 0.03 (18) | 44.1 ± 6.00 (18)*** | 0.3 ± 0.08 (18) | 36.1 ± 4.91 (18)*** | 45.7 ± 5.37 (18)*** | 58.7 ± 8.71(18)*** |  | 0.0 ± 0.00 (8)*** | 0.0 ± 0.04(8)*** |
|  |  | F | 0.1 ± 0.03 (18) | 34.4 ± 3.49 (18)*** | 0.3 ± 0.10 (18) | 42.8 ± 7.66 (18)*** | 40.2 ± 4.25 (18)*** | 49.0 ± 8.21(18)*** |  | 0.0 ± 0.04 (8)*** | 0.0 ± 0.00 (8)*** |
|  | Trans-3-hydroxycotinine  (µmol/L) | M | 0.1 ± 0.04 (18) | 24.2 ± 3.54 (18)*** | 0.4 ± 0.10 (18) | 27.6 ± 4.00 (18)*** | 30.6 ± 3.31 (18)*** | 37.5 ± 4.08(18)*** |  | 0.0 ± 0.05 (8)*** | 0.0 ± 0.00 (8)*** |
|  |  | F | 0.1 ± 0.04 (18) | 12.9 ± 1.99 (18)*** | 0.3 ± 0.08 (18) | 18.0 ± 3.00 (18)*** | 14.7 ± 1.29 (18)*** | 19.0 ± 3.04 (18)*** |  | 0.0 ± 0.04 (8)*** | 0.0 ± 0.00 (8)*** |
|  | Total metabolites  (µmol/L) | M | 1.0 ± 0.15 (18) | 351.8 ± 41.04 (18)*** | 2.5 ± 0.66 (18) | 309.5 ± 35.76 (18)*** | 369.7 ± 39.76 (18)*** | 402.9 ± 48.01 (18)*** |  | 1.1 ± 0.11(8)*** | 1.1 ± 0.11(8)*** |
|  |  | F | 1.3 ± 0.16 (18) | 261.6 ± 24.42 (18)*** | 2.7 ± 0.72 (18) | 309.0 ± 51.12 (18)*** | 244.2 ± 23.47 (18)*** | 310.6 ± 43.19 (18)*** |  | 1.1 ± 0.18 (8)*** | 0.7 ± 0.06 (8)*** |
| Marker of exposure in plasma | Nicotine  (ng/mL) | M | 23.0 ± 0.86 (18) | 307.4 ± 10.46 (18)*** | 16.9 ± 5.06 (18) | 363.5 ± 20.27 (18)*** | 447.5 ± 38.14 (18)*** | 351.5 ± 15.48 (18)*** |  | 85.7 ± 19.88 (8)*** | 81.1 ± 9.25 (8)*** |
|  |  | F | 23.5 ± 1.16 (18) | 417.6 ± 17.31 (18)*** | 17.5 ± 4.42 (18) | 374.3 ± 17.57 (18)*** | 531.0 ± 40.73 (18)*** | 518.5 ± 29.27 (18)*** |  | 79.7 ± 14.54 (8)*** | 126.9 ± 32.61 (8)*** |
|  | Cotinine  (ng/mL) | M | 22.1 ± 5.06 (18) | 1066.7 ± 26.99 (18)*** | 13.4 ± 4.99 (18)* | 868.6 ± 39.44 (18)*** | 890.9 ± 40.65 (18)*** | 857.8 ± 39.21 (18)*** |  | 26.0 ± 8.90 (8)*** | 35.3 ± 4.27 (8)*** |
|  |  | F | 17.8 ± 4.59 (18) | 1042.8 ± 37.12 (18)*** | 10.2 ± 4.76 (18)* | 812.8 ± 45.42 (18)*** | 863.8 ± 41.23 (18)*** | 883.8 ± 42.73 (18)*** |  | 35.4 ± 7.37 (8)*** | 18.0 ± 8.12 (8)*** |
|  | Propylene glycol  (ng/mL) | M |  | 82.8 ± 4.14(18) | 94.6 ± 5.96 (18) | 75.4 ± 3.94 (18) | 80.9 ± 3.82 (18) | 80.6 ± 7.56 (18) |  | 0.0 ± 0.00 (8)*** | 0.0 ± 0.00 (8)*** |
|  |  | F |  | 96.1 ± 4.93(18) | 116.4 ± 3.91 (18) | 81.2 ± 4.28 (18) | 81.5 ± 5.63 (18) | 90.9 ± 4.26 (18) |  | 0.0 ± 0.00 (8)*** | 0.0 ± 0.00 (8)*** |
|  | L-Carvone  (ng/mL) | M |  | 2.0 ± 0.18(18) | 18.8 ± 0.89 (18) | 10.3 ± 0.53 (18) | 29.2 ± 2.25 (18) | 38.4 ± 2.15 (18) |  | 1.5 ± 0.26 (8) | 1.8 ± 0.23 (8)*** |
|  |  | F |  | 1.3 ± 0.13(18) | 16.4 ± 0.93 (18) | 9.3 ± 0.38 (18) | 29.4 ± 1.60 (18) | 45.5 ± 2.64 (18) |  | 1.5 ± 0.21 (8) | 1.6 ± 0.15 (8)*** |
|  | Linalool  (ng/mL) | M |  | 1.4 ± 0.16(18) | 8.3 ± 0.46 (18) | 4.8 ± 0.37 (18) | 14.7 ± 1.69 (18) | 18.5 ± 1.76 (18) |  | 0.9 ± 0.09 (8)* | 0.9 ± 0.06 (8)*** |
|  |  | F |  | 1.2 ± 0.14(18) | 9.5 ± 0.51 (18) | 6.3 ± 0.38 (18) | 16.8 ± 1.05 (18) | 27.9 ± 2.21 (18) |  | 1.0 ± 0.05 (8) | 0.8 ± 0.09 (8)*** |
|  | Citronellol  (ng/mL) | M |  | 0.2 ± 0.07(18) | 5.5 ± 0.64 (18) | 0.5 ± 0.11 (18) | 13.6 ± 1.51 (18) | 16.7 ± 2.14 (18) |  | 0.2 ± 0.11 (8) | 0.1 ± 0.05 (8)*** |
|  |  | F |  | 0.2 ± 0.05(18) | 13.1 ± 1.32 (18) | 1.1 ± 0.09 (18) | 19.8 ± 1.81 (18) | 36.8 ± 3.94 (18) |  | 0.1 ± 0.08 (8) | 0.1 ± 0.09 (8)*** |

*Remark:*

*Results represent mean ± SEM. The sample size is in parentheses.*

*The Exposed groups are compared against PBS during “Exposed,” while the Recovery groups are compared against the same treatment group from “Exposed.” Significance: *, p<0.05; **, p<0.01; ***, p<0.001.*

*Abbreviations: M, male; F, female.*

*The quantification limit of the analytical detection method for nicotine and cotinine are 99.9 ng/mL and 117.5 ng/mL, respectively.*

**Supplemental Table 10 In-life observations**

***A. Group observations***

| **Group name** | **Sex** | **Animals** | **Grooming** | **Harderian gland secretion** | **Piloerection** | **Tremor** |
| --- | --- | --- | --- | --- | --- | --- |
| PBS OECD | M | 10 | 4.1 | 8.4 | 0.0 | 0.0 |
| PG/VG + Nic OECD | M | 10 | 1.4 | 8.9 | 0.0 | 0.1 |
| PG/VG +F-Med OECD | M | 10 | 4.1 | 9.1 | 0.0 | 0.0 |
| PG/VG + Nic +F-Low OECD | M | 10 | 1.2 | 8.9 | 0.0 | 0.0 |
| PG/VG + Nic +F-Med OECD | M | 10 | 1.4 | 9.1 | 0.0 | 0.0 |
| PG/VG + Nic + F-High OECD | M | 10 | 1.2 | 9.0 | 0.0 | 0.0 |
| PG/VG + Nic OECD (R) ^a^ | M | 8 | 1.0 | 7.9 | 0.0 | 0.1 |
| PG/VG + Nic + F-High OECD (R) ^a^ | M | 8 | 1.1 | 7.9 | 0.0 | 0.0 |
| PBS OECD | F | 10 | 3.7 | 8.7 | 0.0 | 0.0 |
| PG/VG + Nic OECD | F | 10 | 1.6 | 8.7 | 0.0 | 0.2 |
| PG/VG +F-Med OECD | F | 10 | 3.5 | 8.9 | 0.0 | 0.0 |
| PG/VG + Nic +F-Low OECD | F | 10 | 1.4 | 8.9 | 0.0 | 0.0 |
| PG/VG + Nic +F-Med OECD | F | 10 | 1.6 | 8.9 | 0.0 | 0.0 |
| PG/VG + Nic + F-High OECD | F | 10 | 1.6 | 8.9 | 0.0 | 0.1 |
| PG/VG + Nic OECD (R) ^a^ | F | 8 | 1.3 | 7.7 | 0.0 | 0.2 |
| PG/VG + Nic + F-High OECD (R) ^a^ | F | 8 | 1.2 | 7.9 | 0.0 | 0.0 |

*Remark:*

*Results are presented as average scores per parameter. The group observation scores were collected on a daily basis (Note: one observation/incidence equal to one score). These values were averaged over the entire exposure period. The higher average score indicated higher incidence of particular observation for the group during exposure phase.*

^a^ *(R) refers to recovery group.*

*Abbreviations: M, male; F, female.*

***B. Individual observations***

| **Group name** | **Sex** | **Reduced activity and decreased response** | **Touch response** | **Righting reflex** | **Reduced limb grip strength** |
| --- | --- | --- | --- | --- | --- |
| PBS OECD | M | 0 | 0 | 1 | 4 |
| PG/VG + Nic OECD | M | 1 | 3 | 3 | 6 |
| PG/VG +F-Med OECD | M | 2 | 0 | 0 | 7 |
| PG/VG + Nic +F-Low OECD | M | 0 | 5 | 0 | 9 |
| PG/VG + Nic +F-Med OECD | M | 2 | 1 | 0 | 7 |
| PG/VG + Nic + F-High OECD | M | 0 | 1 | 0 | 7 |
| PG/VG + Nic OECD (R) ^a^ | M | 1 | 0 | 1 | 7 |
| PG/VG + Nic + F-High OECD (R) ^a^ | M | 0 | 2 | 2 | 13 |
| PBS OECD | F | 0 | 0 | 0 | 2 |
| PG/VG + Nic OECD | F | 0 | 5 | 5 | 10 |
| PG/VG +F-Med OECD | F | 0 | 4 | 0 | 3 |
| PG/VG + Nic +F-Low OECD | F | 0 | 1 | 1 | 14 |
| PG/VG + Nic +F-Med OECD | F | 1 | 3 | 1 | 10 |
| PG/VG + Nic + F-High OECD | F | 0 | 1 | 0 | 3 |
| PG/VG + Nic OECD (R) ^a^ | F | 1 | 6 | 3 | 6 |
| PG/VG + Nic + F-High OECD (R) ^a^ | F | 0 | 0 | 4 | 8 |

*Remark:*

*Individual observations, total incidence of findings per group, i. e., the sum of all findings noted during the study, including finding repeated in the same rat on different observation days.*

^a^ *(R) refers to recovery group.*

*Abbreviations: M, male; F, female.*

**Supplemental Table 11** **Hematology and clinical chemistry**

| **Type** | **Parameter** | **Sex** | **Exposed** | | | | | |  | **Recovery** | |
| --- | --- | --- | --- | --- | --- | --- | --- | --- | --- | --- | --- |
|  |  |  | **PBS** | **PG/VG + Nic** | **PG/VG + F-Med** | **PG/VG + Nic**  **+ F-Low** | **PG/VG + Nic**  **+ F-Med** | **PG/VG + Nic**  **+ F-High** |  | **PG/VG + Nic** | **PG/VG + Nic**  **+ F-High** |
| Red blood cells | Erythrocyte (10^12^/L) | M | 8.703 ± 0.148 (10) | 8.581 ± 0.255 (10) | 8.590 ± 0.226 (10) | 8.427 ± 0.195 (10) | 8.831 ± 0.114 (10) | 8.754 ± 0.114 (10) |  | 8.777 ± 0.182 (8) | 8.816 ± 0.087 (8) |
|  |  | F | 8.093 ± 0.090 (10) | 8.214 ± 0.154 (10) | 8.037 ± 0.085 (10) | 7.886 ± 0.114 (10) | 7.790 ± 0.157 (10) | 7.829 ± 0.090 (10) |  | 7.830 ± 0.117 (8) | 7.964 ± 0.189 (8) |
|  | Reticulocyte (10^12^/L) | M | 0.286 ± 0.018 (10) | 0.281 ± 0.020 (10) | 0.277 ± 0.018 (10) | 0.286 ± 0.017 (10) | 0.295 ± 0.021 (10) | 0.273 ± 0.013 (10) |  | 0.327 ± 0.029 (8) | 0.301 ± 0.017 (8) |
|  |  | F | 0.222 ± 0.015 (10) | 0.255 ± 0.013 (10) | 0.224 ± 0.012 (10) | 0.286 ± 0.021 (10)* | 0.275 ± 0.028 (10) | 0.285 ± 0.019 (10)* |  | 0.290 ± 0.034 (8) | 0.316 ± 0.022 (8) |
|  | Reticulocyte/  Erythrocyte ratio (%) | M | 3.300 ± 0.236 (10) | 3.322 ± 0.302 (10) | 3.251 ± 0.223 (10) | 3.369 ± 0.161 (10) | 3.335 ± 0.240 (10) | 3.135 ± 0.175 (10) |  | 3.729 ± 0.333 (8) | 3.414 ± 0.170 (8) |
|  |  | F | 2.748 ± 0.198 (10) | 3.093 ± 0.135 (10) | 2.794 ± 0.163 (10) | 3.652 ± 0.287 (10)* | 3.544 ± 0.378 (10) | 3.650 ± 0.266 (10)* |  | 3.724 ± 0.483 (8) | 3.989 ± 0.295 (8) |
|  | Hematocrit (L/L) | M | 0.453 ± 0.004 (10) | 0.459 ± 0.013 (10) | 0.447 ± 0.010 (10) | 0.448 ± 0.011 (10) | 0.457 ± 0.005 (10) | 0.469 ± 0.006 (10)* |  | 0.453 ± 0.008 (8) | 0.449 ± 0.010 (8) |
|  |  | F | 0.423 ± 0.005 (10) | 0.450 ± 0.010 (10)* | 0.434 ± 0.005 (10) | 0.435 ± 0.004 (10) | 0.433 ± 0.011 (10) | 0.428 ± 0.005 (10) |  | 0.419 ± 0.004 (8)* | 0.421 ± 0.009 (8) |
|  | Hemoglobin (g/L) | M | 156.150 ± 1.866 (10) | 156.950 ± 5.005 (10) | 153.500 ± 3.720 (10) | 152.200 ± 4.25 (10) | 156.550 ± 1.95 (10) | 161.700 ± 2.41 (10) |  | 157.125 ± 2.682 (8) | 156.938 ± 3.357 (8) |
|  |  | F | 147.800 ± 1.719 (10) | 154.650 ± 3.566 (10) | 151.800 ± 1.635 (10) | 148.450 ± 1.67 (10) | 147.250 ± 3.92 (10) | 145.600 ± 1.87 (10) |  | 146.375 ± 1.352 (8) | 148.063 ± 3.605 (8) |
|  | Mean corpuscular volume (fL) | M | 52.185 ± 0.775 (10) | 53.525 ± 0.582 (10) | 52.170 ± 0.733 (10) | 53.180 ± 0.542 (10) | 51.795 ± 0.606 (10) | 53.585 ± 0.821 (10) |  | 51.688 ± 0.538 (8)* | 50.875 ± 0.851 (8)* |
|  |  | F | 52.325 ± 0.375 (10) | 54.760 ± 0.478 (10)*** | 54.115 ± 0.924 (10) | 55.245 ± 0.471 (10)*** | 55.500 ± 0.444 (10)*** | 54.650 ± 0.629 (10)** |  | 53.531 ± 0.584 (8) | 52.838 ± 0.405 (8)* |
|  | Mean corpuscular hemoglobin (pg) | M | 17.965 ± 0.230 (10) | 18.280 ± 0.139 (10) | 17.895 ± 0.221 (10) | 18.055 ± 0.197 (10) | 17.740 ± 0.209 (10) | 18.490 ± 0.290 (10) |  | 17.925 ± 0.187 (8) | 17.794 ± 0.287 (8) |
|  |  | F | 18.265 ± 0.118 (10) | 18.825 ± 0.143 (10)** | 18.895 ± 0.253 (10)* | 18.845 ± 0.135 (10)** | 18.890 ± 0.174 (10)** | 18.620 ± 0.228 (10) |  | 18.706 ± 0.223 (8) | 18.594 ± 0.113 (8) |
|  | Mean corpuscular hemoglobin concentration (g/dL) | M | 34.460 ± 0.160 (10) | 34.160 ± 0.229 (10) | 34.295 ± 0.101 (10) | 33.940 ± 0.123 (10)* | 34.260 ± 0.106 (10) | 34.505 ± 0.146 (10) |  | 34.669 ± 0.133 (8) | 34.981 ± 0.116 (8)* |
|  |  | F | 34.910 ± 0.132 (10) | 34.350 ± 0.126 (10)** | 34.950 ± 0.214 (10) | 34.105 ± 0.135 (10)** | 34.020 ± 0.162 (10)*** | 34.050 ± 0.166 (10)*** |  | 34.963 ± 0.085 (8)** | 35.194 ± 0.147 (8)*** |
| White blood cells (absolute) | Leukocyte counts (10^9^/L) | M | 10.137 ± 0.771 (10) | 8.182 ± 0.505 (10) | 9.986 ± 1.231 (10) | 8.579 ± 0.656 (10) | 9.667 ± 1.303 (10) | 9.365 ± 1.158 (10) |  | 8.754 ± 0.583 (8) | 11.628 ± 1.630 (8) |
|  |  | F | 7.249 ± 0.480 (10) | 7.018 ± 0.503 (10) | 8.471 ± 0.761 (10) | 7.399 ± 0.471 (10) | 7.446 ± 0.965 (10) | 7.219 ± 0.523 (10) |  | 6.154 ± 0.562 (8) | 7.535 ± 0.827 (8) |
|  | Basophil counts (10^9^/L) | M | 0.010 ± 0.002 (10) | 0.010 ± 0.002 (10) | 0.015 ± 0.005 (10) | 0.008 ± 0.002 (10) | 0.010 ± 0.001 (10) | 0.006 ± 0.001 (10) |  | 0.006 ± 0.001 (8) | 0.009 ± 0.002 (8) |
|  |  | F | 0.010 ± 0.001 (10) | 0.007 ± 0.001 (10) | 0.008 ± 0.002 (10) | 0.009 ± 0.001 (10) | 0.008 ± 0.002 (10) | 0.010 ± 0.001 (10) |  | 0.005 ± 0.002 (8) | 0.011 ± 0.003 (8) |
|  | Eosinophil counts (10^9^/L) | M | 0.118 ± 0.018 (10) | 0.118 ± 0.016 (10) | 0.124 ± 0.018 (9) | 0.128 ± 0.008 (10) | 0.097 ± 0.012 (9) | 0.144 ± 0.030 (9) |  | 0.166 ± 0.031 (8) | 0.207 ± 0.052 (8) |
|  |  | F | 0.113 ± 0.015 (10) | 0.096 ± 0.010 (10) | 0.177 ± 0.060 (9) | 0.079 ± 0.011 (10) | 0.119 ± 0.021 (9) | 0.098 ± 0.008 (9) |  | 0.093 ± 0.015 (8) | 0.088 ± 0.009 (8) |
|  | Lymphocyte counts (10^9^/L) | M | 7.573 ± 0.605 (10) | 5.942 ± 0.473 (10)* | 7.342 ± 0.852 (9) | 6.457 ± 0.559 (10) | 6.048 ± 0.459 (9) | 5.468 ± 0.640 (9)* |  | 6.724 ± 0.638 (8) | 8.984 ± 1.430 (8)* |
|  |  | F | 5.439 ± 0.324 (10) | 4.942 ± 0.357 (10) | 6.414 ± 0.468 (9) | 4.863 ± 0.332 (10) | 4.294 ± 0.628 (9) | 4.378 ± 0.310 (9)* |  | 4.702 ± 0.425 (8) | 5.681 ± 0.603 (8) |
|  | Monocyte counts (10^9^/L) | M | 0.510 ± 0.056 (10) | 0.407 ± 0.031 (10) | 0.456 ± 0.056 (9) | 0.462 ± 0.049 (10) | 0.489 ± 0.065 (9) | 0.444 ± 0.068 (9) |  | 0.523 ± 0.060 (8) | 0.547 ± 0.071 (8) |
|  |  | F | 0.383 ± 0.042 (10) | 0.320 ± 0.042 (10) | 0.346 ± 0.078 (9) | 0.436 ± 0.036 (10) | 0.357 ± 0.048 (9) | 0.416 ± 0.030 (9) |  | 0.298 ± 0.046 (8) | 0.411 ± 0.046 (8) |
|  | Neutrophil counts (10^9^/L) | M | 1.927 ± 0.257 (10) | 1.706 ± 0.241 (10) | 1.199 ± 0.127 (9)* | 1.525 ± 0.184 (10) | 1.843 ± 0.305 (9) | 2.790 ± 0.845 (9) |  | 1.336 ± 0.179 (8) | 1.881 ± 0.327 (8) |
|  |  | F | 1.304 ± 0.294 (10) | 1.618 ± 0.211 (10) | 1.062 ± 0.212 (9) | 1.973 ± 0.273 (10) | 2.183 ± 0.391 (9) | 2.129 ± 0.337 (9) |  | 1.058 ± 0.128 (8)* | 1.344 ± 0.227 (8) |
| White blood cells (relative) | Basophil counts (relative) (%) | M | 0.105 ± 0.024 (10) | 0.115 ± 0.020 (10) | 0.135 ± 0.030 (10) | 0.085 ± 0.028 (10) | 0.115 ± 0.017 (10) | 0.060 ± 0.015 (10) |  | 0.056 ± 0.015 (8)* | 0.081 ± 0.016 (8) |
|  |  | F | 0.120 ± 0.015 (10) | 0.090 ± 0.021 (10) | 0.100 ± 0.025 (10) | 0.120 ± 0.024 (10) | 0.105 ± 0.020 (10) | 0.135 ± 0.018 (10) |  | 0.088 ± 0.030 (8) | 0.163 ± 0.071 (8) |
|  | Eosinophil counts (relative) (%) | M | 1.160 ± 0.141 (10) | 1.400 ± 0.147 (10) | 1.389 ± 0.141 (9) | 1.545 ± 0.137 (10) | 1.122 ± 0.108 (9) | 1.622 ± 0.255 (9) |  | 1.969 ± 0.425 (8) | 1.975 ± 0.477 (8) |
|  |  | F | 1.515 ± 0.135 (10) | 1.425 ± 0.180 (10) | 2.167 ± 0.627 (9) | 1.035 ± 0.082 (10)** | 1.744 ± 0.216 (9) | 1.428 ± 0.137 (9) |  | 1.513 ± 0.221 (8) | 1.175 ± 0.091 (8) |
|  | Lymphocyte counts (relative) (%) | M | 74.560 ± 1.519 (10) | 72.265 ± 2.890 (10) | 79.756 ± 1.312 (9)* | 75.175 ± 2.080 (10) | 71.400 ± 2.665 (9) | 64.133 ± 3.975 (9)** |  | 76.106 ± 2.83 (8) | 76.444 ± 2.834 (8)* |
|  |  | F | 75.960 ± 3.328 (10) | 71.070 ± 2.273 (10) | 80.761 ± 2.216 (9) | 66.265 ± 2.868 (10)* | 61.894 ± 3.513 (9)** | 62.944 ± 3.061 (9)* |  | 76.531 ± 1.83 (8) | 75.994 ± 1.678 (8)** |
|  | Monocyte counts (relative) (%) | M | 5.080 ± 0.489 (10) | 5.065 ± 0.387 (10) | 5.256 ± 0.581 (9) | 5.400 ± 0.481 (10) | 5.756 ± 0.593 (9) | 5.428 ± 0.709 (9) |  | 6.000 ± 0.659 (8) | 4.850 ± 0.354 (8) |
|  |  | F | 5.265 ± 0.405 (10) | 4.685 ± 0.515 (10) | 4.083 ± 0.574 (9) | 6.165 ± 0.610 (10) | 5.611 ± 0.860 (9) | 6.144 ± 0.590 (9) |  | 4.775 ± 0.540 (8) | 5.556 ± 0.413 (8) |
|  | Neutrophil counts (relative) (%) | M | 19.095 ± 1.607 (10) | 21.155 ± 3.013 (10) | 13.489 ± 0.941 (9)** | 17.795 ± 1.874 (10) | 21.600 ± 2.599 (9) | 28.767 ± 4.283 (9)* |  | 15.869 ± 2.37 (8) | 16.650 ± 2.448 (8)** |
|  |  | F | 17.140 ± 2.986 (10) | 22.735 ± 2.144 (10)* | 12.889 ± 1.942 (9) | 26.420 ± 2.947 (10)* | 30.639 ± 3.516 (9)** | 29.344 ± 3.362 (9)* |  | 17.094 ± 1.448 (8)* | 17.112 ± 1.710 (8)** |
| Platelet | Platelet (10^9^/L) | M | 848.250 ± 68.642 (10) | 927.450 ± 33.574 (10) | 921.000 ± 64.095 (10) | 951.300 ± 43.590 (10) | 1013.150 ± 40.453 (10) | 848.050 ± 96.685 (10) |  | 937.313 ± 70.047 (8) | 1042.688 ± 82.812 (8)* |
|  |  | F | 956.150 ± 34.839 (10) | 857.150 ± 48.920 (10) | 859.450 ± 33.782 (10) | 865.150 ± 90.696 (10) | 715.100 ± 94.067 (10)* | 870.000 ± 65.821 (10) |  | 932.688 ± 105.221 (8) | 1187.063 ± 41.530 (8)** |
| Clotting potential | Prothrombin time (s) | M | 16.686 ± 0.365 (7) | 18.822 ± 0.669 (9)* | 17.510 ± 0.316 (10) | 18.589 ± 0.691 (9)** | 18.086 ± 0.396 (7)* | 18.370 ± 0.759 (10)* |  | 16.575 ± 0.51 (8)* | 16.488 ± 0.271 (8)* |
|  |  | F | 16.900 ± 0.601 (7) | 17.556 ± 0.713 (9) | 16.140 ± 0.364 (10) | 21.033 ± 3.543 (9) | 17.990 ± 0.385 (10)* | 17.529 ± 0.495 (7) |  | 15.729 ± 0.20 (7)* | 16.425 ± 0.220 (8) |
|  | Activated partial thromboplastin time (s) | M | 18.929 ± 0.733 (7) | 20.111 ± 0.928 (9) | 19.480 ± 0.904 (10) | 19.411 ± 0.837 (9) | 20.871 ± 0.655 (7) | 21.330 ± 1.140 (10) |  | 21.763 ± 0.37 (8) | 23.937 ± 1.328 (8) |
|  |  | F | 19.871 ± 2.551 (7) | 20.344 ± 0.475 (9) | 19.420 ± 0.639 (10) | 21.444 ± 2.934 (9) | 18.860 ± 0.942 (10) | 19.929 ± 1.245 (7) |  | 22.129 ± 0.846(7) | 20.163 ± 0.758 (8) |
|  | Fibrinogen (g/L) | M | 2.519 ± 0.030 (7) | 2.597 ± 0.211 (9) | 2.430 ± 0.174 (10) | 3.013 ± 0.222 (9) | 2.780 ± 0.151 (7) | 2.811 ± 0.311 (10) |  | 2.100 ± 0.265 (8) | 2.211 ± 0.310 (8) |
|  |  | F | 2.123 ± 0.404 (7) | 2.726 ± 0.134 (9) | 2.161 ± 0.201 (10) | 3.389 ± 0.203 (8)* | 3.144 ± 0.264 (10) | 2.789 ± 0.248 (7) |  | 1.897 ± 0.236 (7)* | 2.148 ± 0.138 (8)* |
| Bone marrow evaluation differential relative | Myeloid/Erythroid Ratio | M | 1.583 ± 0.123 (10) | 2.345 ± 0.226 (10)** | 1.860 ± 0.163 (10) | 2.953 ± 0.280 (10)*** | 2.337 ± 0.170 (10)*** | 2.041 ± 0.185 (10) |  | 1.599 ± 0.071 (8)** | 1.874 ± 0.155 (8) |
|  |  | F | 1.804 ± 0.149 (10) | 1.905 ± 0.241 (10) | 2.132 ± 0.244 (10) | 1.668 ± 0.084 (10) | 2.297 ± 0.226 (10) | 2.053 ± 0.113 (10) |  | 1.615 ± 0.165 (8) | 1.588 ± 0.137 (8)* |
| Minerals | Calcium (mmol/L) | M | 2.40 ± 0.027 (10) | 2.35 ± 0.036 (10) | 2.36 ± 0.024 (10) | 2.40 ± 0.041 (10) | 2.34 ± 0.021 (10) | 2.33 ± 0.035 (10) |  | 2.47 ± 0.029 (8)* | 2.44 ± 0.024 (8)* |
|  |  | F | 2.43 ± 0.026 (10) | 2.40 ± 0.065 (10) | 2.40 ± 0.050 (10) | 2.35 ± 0.018 (10)* | 2.39 ± 0.033 (10) | 2.38 ± 0.023 (10) |  | 2.54 ± 0.049 (8)* | 2.56 ± 0.034 (8)*** |
|  | Inorganic phosphate (mmol/L) | M | 2.20 ± 0.073 (10) | 2.32 ± 0.099 (10) | 2.56 ± 0.086 (10)** | 2.69 ± 0.146 (10)** | 2.59 ± 0.096 (10)** | 2.52 ± 0.070 (10)** |  | 2.68 ± 0.073 (8)** | 2.53 ± 0.064 (8) |
|  |  | F | 2.16 ± 0.074 (10) | 2.39 ± 0.165 (10) | 2.24 ± 0.110 (10) | 2.45 ± 0.116 (10)* | 2.34 ± 0.067 (10) | 2.50 ± 0.085 (10)** |  | 2.07 ± 0.088 (8) | 2.21 ± 0.092 (8)* |
| Proteins | Total protein (g/L) | M | 60.55 ± 0.677 (10) | 57.35 ± 1.121 (10)* | 57.15 ± 1.414 (10)* | 58.40 ± 1.211 (10) | 58.25 ± 0.549 (10)* | 56.85 ± 0.760 (10)** |  | 62.31 ± 0.422 (8)** | 61.81 ± 1.017 (8)** |
|  |  | F | 63.40 ± 1.185 (10) | 59.05 ± 1.369 (10)* | 63.65 ± 1.368 (10) | 59.25 ± 0.765 (10)** | 58.40 ± 1.154 (10)** | 60.40 ± 0.649 (10)* |  | 67.19 ± 1.835 (8)** | 68.06 ± 0.868 (8)*** |
|  | Albumin (g/L) | M | 34.72 ± 0.361 (10) | 33.30 ± 0.606 (10) | 33.71 ± 0.635 (10) | 34.00 ± 0.652 (10) | 34.33 ± 0.409 (10) | 34.02 ± 0.365 (10) |  | 35.94 ± 0.431 (8)** | 34.91 ± 0.414 (8) |
|  |  | F | 37.40 ± 0.701 (10) | 34.11 ± 0.748 (10)** | 37.91 ± 0.914 (10) | 33.51 ± 0.430 (10)*** | 33.32 ± 0.559 (10)*** | 34.34 ± 0.310 (10)** |  | 38.53 ± 1.079 (8)** | 38.74 ± 0.661 (8)*** |
|  | Globulin (g/L) | M | 25.84 ± 0.436 (10) | 24.06 ± 0.872 (10) | 23.45 ± 0.801 (10)* | 24.40 ± 0.772 (10) | 23.92 ± 0.326 (10)** | 22.84 ± 0.516 (10)*** |  | 26.38 ± 0.375 (8)* | 26.90 ± 0.776 (8)*** |
|  |  | F | 26.00 ± 0.734 (10) | 24.94 ± 0.765 (10) | 25.74 ± 0.686 (10) | 25.74 ± 0.859 (10) | 25.09 ± 0.667 (10) | 26.06 ± 0.616 (10) |  | 28.66 ± 0.912 (8)** | 29.32 ± 0.447 (8)*** |
|  | Urea (mmol/L) | M | 6.90 ± 0.301 (10) | 6.67 ± 0.288 (10) | 6.09 ± 0.170 (10)* | 5.97 ± 0.180 (10)* | 5.76 ± 0.175 (10)** | 6.65 ± 0.192 (10) |  | 6.33 ± 0.206 (8) | 6.48 ± 0.271 (8) |
|  |  | F | 6.15 ± 0.120 (10) | 5.65 ± 0.276 (10) | 5.75 ± 0.210 (10)* | 5.78 ± 0.171 (10) | 6.38 ± 0.840 (10) | 5.28 ± 0.230 (10)** |  | 5.96 ± 0.260 (8) | 6.44 ± 0.133 (8)*** |
| Electrolytes | Chloride (mmol/L) | M | 104.25 ± 0.602 (10) | 105.95 ± 1.010 (10) | 105.40 ± 0.884 (10) | 104.95 ± 0.508 (10) | 106.30 ± 0.831 (10) | 105.85 ± 0.789 (10) |  | 101.19 ± 0.52 (8)** | 100.00 ± 0.535 (8)*** |
|  |  | F | 104.95 ± 0.647 (10) | 106.70 ± 1.065 (10) | 105.45 ± 0.740 (10) | 105.60 ± 0.816 (10) | 107.05 ± 1.042 (10) | 106.75 ± 1.014 (10) |  | 100.94 ± 0.546(8)*** | 101.88 ± 0.603 (8)*** |
|  | Potassium (mmol/L) | M | 4.34 ± 0.137 (10) | 4.69 ± 0.298 (10) | 4.66 ± 0.166 (10) | 5.24 ± 0.425 (10) | 4.44 ± 0.130 (10) | 5.02 ± 0.363 (10) |  | 4.93 ± 0.358 (8) | 4.78 ± 0.223 (8) |
|  |  | F | 4.10 ± 0.138 (10) | 4.58 ± 0.499 (10) | 4.10 ± 0.136 (10) | 4.39 ± 0.140 (10) | 4.35 ± 0.271 (10) | 4.60 ± 0.284 (10) |  | 4.15 ± 0.145 (8) | 4.32 ± 0.257 (8) |
|  | Sodium (mmol/L) | M | 143.25 ± 0.455 (10) | 144.40 ± 0.552 (10) | 142.80 ± 0.455 (10) | 144.50 ± 0.601 (10) | 144.80 ± 0.327 (10)* | 143.75 ± 0.574 (10) |  | 143.56 ± 0.34 (8) | 142.38 ± 0.515 (8) |
|  |  | F | 142.75 ± 0.496 (10) | 143.65 ± 0.342 (10) | 141.80 ± 0.367 (10) | 144.05 ± 0.383 (10) | 142.95 ± 0.497 (10) | 144.50 ± 0.247 (10)** |  | 141.94 ± 0.333 (8)** | 141.75 ± 0.463 (8)*** |
| Muscle | Creatinine (µmol/L) | M | 39.99 ± 2.396 (10) | 37.26 ± 2.168 (10) | 37.80 ± 2.499 (10) | 37.47 ± 1.941 (10) | 33.47 ± 1.200 (10)* | 34.17 ± 2.441 (10) |  | 37.86 ± 1.187 (8) | 39.19 ± 1.634 (8) |
|  |  | F | 34.74 ± 1.622 (10) | 32.36 ± 3.170 (10) | 33.35 ± 0.965 (10) | 30.21 ± 1.495 (10) | 38.57 ± 6.817 (10) | 30.15 ± 0.835 (10)* |  | 36.49 ± 1.170 (8) | 35.64 ± 1.328 (8)** |
| Energy metabolism | Glucose (mmol/L) | M | 12.12 ± 0.491 (10) | 10.72 ± 0.646 (10) | 13.69 ± 1.214 (10) | 11.73 ± 1.205 (10) | 9.87 ± 0.313 (10)** | 10.70 ± 0.803 (10) |  | 11.05 ± 0.656 (8) | 13.84 ± 0.895 (8)* |
|  |  | F | 10.13 ± 0.269 (10) | 9.62 ± 0.606 (10) | 10.55 ± 0.270 (10) | 7.79 ± 0.298 (10)*** | 9.00 ± 0.410 (10)* | 9.00 ± 0.221 (10)** |  | 10.97 ± 0.479 (8)* | 10.80 ± 0.669 (8)* |
|  | Triglyceride (mmol/L) | M | 1.05 ± 0.126 (10) | 0.78 ± 0.154 (10) | 1.09 ± 0.128 (10) | 1.18 ± 0.198 (10) | 0.89 ± 0.131 (10) | 0.92 ± 0.105 (10) |  | 1.33 ± 0.164 (8)* | 1.50 ± 0.266 (8) |
|  |  | F | 0.84 ± 0.138 (10) | 0.86 ± 0.129 (10) | 1.07 ± 0.199 (10) | 0.70 ± 0.087 (10) | 0.67 ± 0.104 (10) | 0.63 ± 0.061 (10) |  | 1.45 ± 0.307 (8) | 1.38 ± 0.348 (8) |
|  | Total cholesterol (mmol/L) | M | 1.38 ± 0.089 (10) | 1.04 ± 0.070 (10)** | 1.24 ± 0.081 (10) | 0.98 ± 0.070 (10)** | 0.90 ± 0.051 (10)** | 0.97 ± 0.085 (10)** |  | 1.33 ± 0.102 (8)* | 1.34 ± 0.073 (8)* |
|  |  | F | 1.10 ± 0.046 (10) | 0.98 ± 0.073 (10) | 1.27 ± 0.086 (10) | 1.05 ± 0.047 (10) | 1.04 ± 0.063 (10) | 0.85 ± 0.041 (10)*** |  | 1.51 ± 0.120 (8)** | 1.61 ± 0.121 (8)*** |
|  | Total bilirubin (µmol/L) | M | 5.80 ± 0.428 (10) | 5.59 ± 0.417 (10) | 6.11 ± 0.689 (10) | 5.19 ± 0.606 (10) | 5.41 ± 0.296 (10) | 6.05 ± 0.479 (10) |  | 6.80 ± 0.657 (8) | 6.94 ± 0.381 (8) |
|  |  | F | 5.50 ± 0.561 (10) | 5.56 ± 0.845 (10) | 4.75 ± 0.422 (10) | 5.47 ± 0.794 (10) | 5.25 ± 0.314 (10) | 4.82 ± 0.348 (10) |  | 5.46 ± 0.451 (8) | 6.47 ± 0.599 (8)* |
| Liver | Aspartate aminotransferase (IU/L) | M | 111.40 ± 6.056 (10) | 148.05 ± 29.340 (10) | 120.05 ± 11.456 (10) | 138.40 ± 11.394 (10) | 122.55 ± 12.546(10) | 289.95 ± 114.777 (10)** |  | 96.50 ± 5.973 (8)* | 102.50 ± 5.324 (8)*** |
|  |  | F | 178.35 ± 40.156 (10) | 153.95 ± 16.854 (10) | 131.70 ± 14.366 (10) | 145.75 ± 13.411(10) | 139.20 ± 14.098(10) | 119.10 ± 6.063 (10)* |  | 93.63 ± 19.048 (8)** | 105.06 ± 16.268 (8) |
|  | Alkaline phosphatase (IU/L) | M | 176.60 ± 17.574 (10) | 167.25 ± 19.492 (10) | 173.90 ± 18.293 (10) | 200.85 ± 19.502 (10) | 221.35 ± 21.295(10) | 194.45 ± 21.654 (10) |  | 147.13 ± 16.720 (8) | 171.56 ± 13.469 (8) |
|  |  | F | 124.30 ± 9.791 (10) | 154.70 ± 23.148 (10) | 113.70 ± 10.366 (10) | 190.60 ± 19.125 (10)** | 186.00 ± 15.764(10)** | 165.15 ± 23.420 (10) |  | 74.00 ± 7.081 (8)** | 88.00 ± 9.246 (8)** |
|  | Alanine aminotransferase (IU/L) | M | 60.05 ± 4.089 (10) | 72.65 ± 14.652 (10) | 55.75 ± 6.344 (10) | 66.35 ± 5.888 (10) | 58.25 ± 3.613 (10) | 152.90 ± 66.575 (10)* |  | 44.75 ± 0.732 (8)* | 48.00 ± 3.572 (8)** |
|  |  | F | 55.30 ± 6.598 (10) | 65.20 ± 5.058 (10) | 58.90 ± 16.840 (10) | 64.00 ± 1.959 (10)** | 66.80 ± 5.076 (10)* | 65.10 ± 1.915 (10) |  | 43.69 ± 8.420 (8)* | 49.44 ± 9.066 (8) |

*Remarks:*

*Results represent mean ± SEM. The sample size is in parentheses.*

*The Exposed groups are compared against PBS during “Exposed,” while the Recovery groups are compared against the same treatment group from “Exposed.” Significance: *, p<0.05; **, p<0.01; ***, p<0.001.*

*Abbreviations: M, male; F, female.*

**Supplemental Table 12** **Differential counts of free lung cells and multiple analyte analysis in BALF**

| **Type** | **Parame-ter** | **Sex** | **Exposed** | | | | | |  | **Recovery** | |
| --- | --- | --- | --- | --- | --- | --- | --- | --- | --- | --- | --- |
|  |  |  | **PBS** | **PG/VG + Nic** | **PG/VG + F-Med** | **PG/VG + Nic + F-Low** | **PG/VG + Nic + F-Med** | **PG/VG + Nic + F-High** |  | **PG/VG + Nic** | **PG/VG + Nic + F-High** |
| Free lung cells collection | Total cell counts | M | 1407814 ± 275515.2 (10) | 1428578 ± 176163.2 (10) | 1438671 ± 285713.8 (10) | 1505442 ± 205176.7 (10) | 1384646 ± 157548.1 (10) | 984386 ± 105174.8 (10) |  | 2122736 ± 388892.4 (8) | 1704413 ± 233721.8 (8)* |
|  |  | F | 956664 ± 69238.3 (10) | 946458 ± 108443.6 (10) | 1284121 ± 161536.0 (10) | 1317909 ± 253606.9 (10) | 1320277 ± 113864.6 (10)* | 1085102 ± 182388.2 (10) |  | 1310824 ± 113634.9 (8)* | 1192119 ± 130544.5 (8) |
| Free lung cells event | Total | M | 49542.2 ± 2335.20 (10) | 48007.8 ± 1045.69 (10) | 49737.7 ± 2003.24 (10) | 50565.8 ± 570.59 (10) | 48964.0 ± 706.00 (10) | 47030.9 ± 2096.71 (10) |  | 47071.9 ± 2923.56 (8) | 49994.8 ± 4.46 (8) |
|  |  | F | 48026.4 ± 2374.50 (10) | 52105.1 ± 1997.29 (10) | 48783.2 ± 3695.53 (10) | 47980.6 ± 3291.61 (10) | 50303.8 ± 340.84 (10) | 47467.4 ± 2768.58 (10) |  | 53071.5 ± 3080.65 (8) | 49990.0 ± 5.12 (8) |
|  | Dead cells | M | 266.5 ± 88.01 (10) | 206.2 ± 20.40 (10) | 159.1 ± 19.37 (10) | 200.3 ± 23.83 (10) | 273.3 ± 53.41 (10) | 195.6 ± 29.54 (10) |  | 186.9 ± 27.50 (8) | 305.4 ± 76.37 (8) |
|  |  | F | 158.7 ± 19.68 (10) | 322.3 ± 146.71(10) | 202.3 ± 24.03 (10) | 250.5 ± 29.01 (10)* | 275.9 ± 73.05 (10) | 159.2 ± 16.08 (10) |  | 170.5 ± 31.45 (8) | 179.4 ± 21.64 (8) |
|  | Living cells | M | 5243.7 ± 809.84 (10) | 5181.6 ± 584.04 (10) | 4590.8 ± 696.62 (10) | 5137.6 ± 493.88 (10) | 5336.9 ± 574.65 (10) | 3285.9 ± 355.91 (10)* |  | 5912.0 ± 913.57 (8) | 5572.8 ± 897.66 (8)* |
|  |  | F | 3773.5 ± 274.99 (10) | 3822.1 ± 659.85 (10) | 5177.8 ± 519.85 (10)* | 5143.4 ± 696.62 (10) | 5595.3 ± 671.12 (10)* | 3374.1 ± 177.37 (10) |  | 4888.3 ± 520.31 (8)* | 3827.6 ± 431.38 (8) |
|  | Viability (%) | M | 95.6 ± 0.60 (10) | 95.9 ± 0.54 (10) | 96.2 ± 0.66 (10) | 96.2 ± 0.37 (10) | 95.3 ± 0.53 (10) | 94.0 ± 0.80 (10) |  | 96.9 ± 0.21 (8) | 94.9 ± 0.82 (8) |
|  |  | F | 95.8 ± 0.56 (10) | 92.5 ± 2.93(10) | 96.0 ± 0.58 (10) | 94.8 ± 0.84(10) | 95.7 ± 0.71 (10) | 95.4 ± 0.47 (10) |  | 96.7 ± 0.35 (8) | 95.4 ± 0.43 (8) |
|  | Eosinophil | M | 31.1 ± 8.52 (10) | 25.3 ± 3.53 (10) | 89.0 ± 46.80 (10) | 31.5 ± 11.66 (10) | 38.2 ± 8.35 (10) | 47.0 ± 16.90 (10) |  | 31.0 ± 5.42 (8) | 21.9 ± 4.35 (8) |
|  |  | F | 65.4 ± 20.88 (10) | 101.2 ± 59.82 (10) | 53.7 ± 13.36 (10) | 35.1 ± 8.78(10) | 80.7 ± 62.85 (10) | 40.9 ± 10.96 (10) |  | 34.4 ± 14.40 (8) | 23.9 ± 3.89 (8) |
|  | Lymphocyte | M | 576.8 ± 119.76 (10) | 439.3 ± 90.27 (10) | 985.8 ± 333.67 (10) | 503.1 ± 106.56(10) | 371.9 ± 70.75 (10) | 497.2 ± 131.91 (10) |  | 1121.8 ± 505.22 (8) | 763.3 ± 172.04 (8) |
|  |  | F | 427.5 ± 60.59 (10) | 1664.3 ± 1194.09 (10) | 862.6 ± 494.83 (10) | 479.4 ± 97.00(10) | 478.0 ± 191.44 (10) | 346.3 ± 50.76 (10) |  | 711.0 ± 150.75 (8) | 537.4 ± 113.48 (8) |
|  | Neutrophil | M | 765.5 ± 432.03 (10) | 534.5 ± 103.79 (10) | 853.8 ± 252.84 (10) | 877.9 ± 311.75(10) | 395.3 ± 64.55 (10) | 1610.2 ± 1091.09 (10) |  | 368.4 ± 106.33 (8) | 334.3 ± 63.14 (8) |
|  |  | F | 494.6 ± 151.35 (10) | 1530.4 ± 930.31 (10)* | 522.5 ± 118.21 (10) | 1658.4 ± 442.49(10)** | 510.3 ± 104.48 (10) | 800.7 ± 202.12 (10) |  | 416.9 ± 106.02 (8) | 315.0 ± 102.29 (8)* |
| Free lung cells count | Eosinophil count | M | 763 ± 150 (10) | 728 ± 115 (10) | 2844 ± 1292 (10) | 890 ± 300 (10) | 1117 ± 322 (10) | 972 ± 333 (10) |  | 1653 ± 489 (8) | 716 ± 150 (8) |
|  |  | F | 1344 ± 433 (10) | 2458 ± 1656 (10) | 1196 ± 204 (10) | 969 ± 317 (10) | 1642 ± 1146 (10) | 917 ± 227 (10) |  | 802 ± 314 (8) | 605 ± 144 (8) |
|  | Lymphocyte count | M | 16183 ± 3995 (10) | 11962 ± 2235 (10) | 40053 ± 19678 (10) | 15522 ± 4000 (10) | 10574 ± 2608 (10) | 9363 ± 2033 (10) |  | 69679 ± 40292 (8) | 23408 ± 3971 (8)** |
|  |  | F | 8978 ± 1751 (10) | 41343 ± 33144 (10) | 17647 ± 9285 (10) | 12832 ± 3639 (10) | 11213 ± 3382 (10) | 8335 ± 2099 (10) |  | 16911 ± 3230 (8) | 13565 ± 3498 (8) |
|  | Macrophage count | M | 1374870 ± 273216 (10) | 1400200 ± 174282 (10) | 1364021 ± 257460 (10) | 1463426 ± 202240 (10) | 1362424 ± 156248 (10) | 946671 ± 110874 (10) |  | 2030332 ± 382498 (8) | 1669382 ± 231704 (8)* |
|  |  | F | 935785 ± 69326 (10) | 866377 ± 87418 (10) | 1251668 ± 162304 (10) | 1265845 ± 250922 (10) | 1294608 ± 114726 (10)* | 1058799 ± 179720 (10) |  | 1283408 ± 114119 (8)* | 1171011 ± 128058 (8) |
|  | Neutrophil count | M | 15998 ± 6678 (10) | 15688 ± 3865 (10) | 31754 ± 14356 (10) | 25603 ± 11133 (10) | 10531 ± 1606 (10) | 27380 ± 16834 (10) |  | 21071 ± 7911 (8) | 10907 ± 2206 (8) |
|  |  | F | 10556 ± 3508 (10) | 36281 ± 26077 (10) | 13610 ± 3325 (10) | 38264 ± 9894 (10)** | 12814 ± 2258 (10) | 17051 ± 3798 (10) |  | 9703 ± 2260 (8) | 6938 ± 2092 (8)* |
| Free Lung cells relative count | Eosinophil count  (relative) (%) | M | 0.1 ± 0.01 (10) | 0.1 ± 0.01 (10) | 0.2 ± 0.09 (10) | 0.1 ± 0.02 (10) | 0.1 ± 0.02 (10) | 0.1 ± 0.03 (10) |  | 0.1 ± 0.02 (8) | 0.0 ± 0.01 (8) |
|  |  | F | 0.1 ± 0.04 (10) | 0.2 ± 0.11 10) | 0.1 ± 0.02 (10) | 0.1 ± 0.02 (10) | 0.2 ± 0.13 (10) | 0.1 ± 0.02(10) |  | 0.1 ± 0.03 (8) | 0.0 ± 0.01 (8) |
|  | Lymphocyte count (relative) (%) | M | 1.2 ± 0.24 (10) | 0.9 ± 0.19 (10) | 2.0 ± 0.67 (10) | 1.0 ± 0.21 (10) | 0.8 ± 0.15 (10) | 1.0 ± 0.26 (10) |  | 3.2 ± 2.00 (8) | 1.5 ± 0.34 (8) |
|  |  | F | 1.0 ± 0.22 (10) | 3.1 ± 2.17 (10) | 1.7 ± 0.99 (10) | 1.0 ± 0.18 (10) | 1.0 ± 0.38 (10) | 0.8 ± 0.15 (10) |  | 1.4 ± 0.30 (8) | 1.1 ± 0.23 (8) |
|  | Macrophage count (relative) (%) | M | 97.3 ± 0.86 (10) | 97.9 ± 0.35 (10) | 96.2 ± 1.16 (10) | 97.2 ± 0.76 (10) | 98.4 ± 0.25 (10) | 95.5 ± 2.44 (10) |  | 95.7 ± 2.38 (8) | 97.8 ± 0.45 (8) |
|  |  | F | 97.7 ± 0.67 (10) | 93.8 ± 3.96 (10) | 97.1 ± 1.08 (10) | 95.5 ± 0.94 (10)* | 97.9 ± 0.68 (10) | 97.4 ± 0.52 (10) |  | 97.8 ± 0.48 (8) | 98.2 ± 0.40 (8) |
|  | Neutrophil count (relative) (%) | M | 1.4 ± 0.75 (10) | 1.1 ± 0.20 (10) | 1.7 ± 0.49 (10) | 1.8 ± 0.62 (10) | 0.8 ± 0.13 (10) | 3.3 ± 2.17 (10) |  | 0.9 ± 0.38 (8) | 0.7 ± 0.13 (8) |
|  |  | F | 1.2 ± 0.46 (10) | 2.9 ± 1.69 (10)* | 1.1 ± 0.23 (10) | 3.4 ± 0.83 (10)** | 1.0 ± 0.21 (10) | 1.8 ± 0.44 (10) |  | 0.8 ± 0.22 (8)* | 0.6 ± 0.21 (8)* |
| BALF protein analysis | EGF (ng/L) | M | 0.2 ± 0.02 (10) | 0.2 ± 0.03 (10) | 0.2 ± 0.04 (10) | 2.5 ± 2.30 (10) | 0.2 ± 0.02 (10) | 0.2 ± 0.02 (10) |  | 0.2 ± 0.04 (8) | 0.2 ± 0.00 (8) |
|  |  | F | 0.2 ± 0.02 (10) | 0.2 ± 0.02 (10) | 0.2 ± 0.01 (10) | 0.5 ± 0.34 (10) | 0.3 ± 0.13 (10) | 0.2 ± 0.00 (10) |  | 0.2 ± 0.01 (8) | 0.2 ± 0.00 (8) |
|  | Eotaxin (ng/L) | M | 4.3 ± 1.10 (10) | 2.2 ± 0.25 (10) | 3.4 ± 0.40 (10) | 2.7 ± 0.58 (10) | 2.3 ± 0.31 (10) | 2.8 ± 0.47 (10) |  | 2.7 ± 0.56 (8) | 2.8 ± 0.34 (8) |
|  |  | F | 3.9 ± 0.65 (10) | 3.2 ± 0.49 (10) | 3.2 ± 0.37 (10) | 2.4 ± 0.35 (10) | 3.1 ± 0.38 (10) | 2.3 ± 0.30 (10)* |  | 2.3 ± 0.37 (8) | 3.2 ± 0.55 (8) |
|  | Fractalkine (ng/L) | M | 11.5 ± 2.43 (10) | 4.8 ± 0.93 (10)* | 5.8 ± 0.88 (10) | 6.9 ± 0.73 (10) | 5.4 ± 0.90 (10)* | 5.2 ± 0.83 (10)* |  | 6.2 ± 0.37 (8) | 7.0 ± 1.44 (8) |
|  |  | F | 9.9 ± 1.51 (10) | 6.8 ± 0.94 (10) | 7.9 ± 1.30 (10) | 6.0 ± 1.09 (10) | 6.6 ± 0.99 (10) | 5.1 ± 0.89 (10)* |  | 9.0 ± 1.68 (8) | 6.2 ± 1.08 (8) |
|  | G-CSF (ng/L) | M | 2.4 ± 0.08 (10) | 1.9 ± 0.27 (10) | 2.0 ± 0.28 (10) | 1.6 ± 0.24 (10)* | 2.3 ± 0.11 (10) | 2.2 ± 0.18 (10) |  | 1.7 ± 0.25 (8) | 2.0 ± 0.20 (8) |
|  |  | F | 2.0 ± 0.14 (10) | 2.2 ± 0.27 (10) | 2.2 ± 0.18 (10) | 1.8 ± 0.19 (10) | 1.9 ± 0.20 (10) | 1.6 ± 0.18 (10) |  | 1.7 ± 0.23 (8) | 2.2 ± 0.26 (8) |
|  | GM-CSF (ng/L) | M | 10.8 ± 3.08 (10) | 5.8 ± 1.53 (10) | 13.0 ± 6.16 (10) | 5.9 ± 1.87 (10) | 7.0 ± 2.84 (10) | 5.9 ± 1.91 (10) |  | 10.7 ± 4.48 (8) | 4.8 ± 1.42 (8) |
|  |  | F | 7.4 ± 2.34 (10) | 4.3 ± 0.91 (10) | 7.1 ± 2.06 (10) | 6.6 ± 2.43 (10) | 9.5 ± 3.59 (10) | 7.3 ± 3.17 (10) |  | 11.0 ± 4.94 (8) | 12.0 ± 4.09 (8) |
|  | KC (ng/L) | M | 22.6 ± 4.31 (10) | 16.1 ± 2.80 (10) | 21.4 ± 4.08 (10) | 19.6 ± 3.04 (10) | 15.3 ± 2.11 (10) | 17.5 ± 3.23 (10) |  | 14.0 ± 2.24 (8) | 16.3 ± 2.84 (8) |
|  |  | F | 23.0 ± 9.87 (10) | 23.2 ± 4.31 (10) | 18.6 ± 3.08 (10) | 14.9 ± 2.25 (10) | 17.5 ± 2.92 (10) | 18.3 ± 2.87 (10) |  | 21.7 ± 8.23 (8) | 22.3 ± 4.76 (8) |
|  | IFN-g (ng/L) | M | 7.3 ± 0.00 (10) | 7.3 ± 0.00 (10) | 7.3 ± 0.00 (10) | 7.4 ± 0.11 (10) | 7.3 ± 0.00 (10) | 7.3 ± 0.00 (10) |  | 7.3 ± 0.00 (8) | 7.3 ± 0.00 (8) |
|  |  | F | 7.3 ± 0.00 (10) | 7.3 ± 0.00 (10) | 7.3 ± 0.00 (10) | 7.3 ± 0.00 (10) | 7.3 ± 0.00 (10) | 7.3 ± 0.00 (10) |  | 7.3 ± 0.00 (8) | 7.3 ± 0.00 (8) |
|  | IL-1a (ng/L) | M | 8.3 ± 1.28 (10) | 6.3 ± 0.35 (10) | 5.9 ± 0.14 (10)* | 7.1 ± 1.32 (10) | 6.0 ± 0.31 (10) | 5.9 ± 0.24 (10) |  | 7.0 ± 0.72 (8) | 7.3 ± 0.82 (8) |
|  |  | F | 6.1 ± 0.90 (10) | 7.7 ± 0.91 (10) | 8.3 ± 1.33 (10) | 6.5 ± 0.41 (10) | 6.9 ± 0.83 (10) | 5.9 ± 0.23 (10) |  | 6.2 ± 0.81 (8) | 6.2 ± 0.47 (8) |
|  | IL-1b (ng/L) | M | 3.3 ± 1.13 (10) | 3.4 ± 1.54 (10) | 3.4 ± 0.95 (10) | 2.3 ± 0.49 (10) | 1.8 ± 0.29 (10) | 3.3 ± 1.03 (10) |  | 3.1 ± 0.89 (8) | 4.0 ± 1.58 (8) |
|  |  | F | 1.6 ± 0.15 (10) | 4.5 ± 2.05 (10) | 2.1 ± 0.42 (10) | 2.6 ± 0.79 (10) | 2.6 ± 0.63 (10) | 3.8 ± 1.25 (10) |  | 1.7 ± 0.33 (8) | 2.6 ± 0.85 (8) |
|  | IL-2 (ng/L) | M | 5.9 ± 0.41 (10) | 5.3 ± 0.53 (10) | 5.8 ± 0.90 (10) | 5.6 ± 1.04 (10) | 5.3 ± 0.47 (10) | 7.1 ± 0.98 (10) |  | 6.5 ± 0.75 (8) | 6.9 ± 1.17 (8) |
|  |  | F | 6.7 ± 1.25 (10) | 4.6 ± 0.40(10) | 5.3 ± 0.40 (10) | 5.6 ± 0.81 (10) | 5.8 ± 0.77 (10) | 6.2 ± 0.66 (10) |  | 5.3 ± 0.75 (8) | 6.8 ± 1.50 (8) |
|  | IL-4 (ng/L) | M | 9.1 ± 2.61 (10) | 8.0 ± 4.77(10) | 13.7 ± 4.97 (10) | 11.9 ± 8.06 (10) | 3.2 ± 1.70 (10)* | 6.8 ± 2.63 (10) |  | 6.5 ± 3.48 (8) | 10.5 ± 3.13 (8) |
|  |  | F | 5.8 ± 1.63 (10) | 15.4 ± 6.52 (10) | 12.0 ± 5.54 (10) | 6.4 ± 1.73 (10) | 5.9 ± 1.99 (10) | 12.0 ± 3.88 (10) |  | 13.9 ± 6.90 (8) | 8.8 ± 5.34 (8) |
|  | IL-5 (ng/L) | M | 13.0 ± 3.60 (10) | 14.2 ± 2.53 (10) | 11.7 ± 2.58 (10) | 7.2 ± 1.53 (10) | 9.6 ± 2.92 (10) | 8.7 ± 2.35 (10) |  | 13.7 ± 5.03 (8) | 10.6 ± 2.87 (8) |
|  |  | F | 9.2 ± 1.81 (10) | 9.9 ± 2.51 (10) | 12.7 ± 3.50 (10) | 12.4 ± 3.35 (10) | 7.2 ± 2.41 (10) | 10.4 ± 2.64 (10) |  | 9.2 ± 3.00 (8) | 8.1 ± 2.18 (8) |
|  | IL-6 (ng/L) | M | 118.8 ± 34.27 (10) | 94.3 ± 22.06 (10) | 93.0 ± 15.98 (10) | 105.9 ± 35.04 (10) | 65.3 ± 12.17 (10) | 95.4 ± 25.24 (10) |  | 97.5 ± 26.26 (8) | 88.9 ± 23.11 (8) |
|  |  | F | 122.5 ± 34.78 (10) | 110.0 ± 45.21 (10) | 120.4 ± 34.64 (10) | 76.2 ± 17.29 (10) | 86.8 ± 13.63 (10) | 106.3 ± 22.37 (10) |  | 110.7 ± 32.13 (8) | 96.8 ± 17.03 (8) |
|  | IL-10 (ng/L) | M | 4.6 ± 0.52 (10) | 3.1 ± 0.19 (10)** | 3.1 ± 0.48 (10)* | 3.1 ± 0.30 (10)* | 3.4 ± 0.29 (10)* | 3.1 ± 0.27 (10)* |  | 3.3 ± 0.28 (8) | 3.1 ± 0.31 (8) |
|  |  | F | 4.6 ± 0.73 (10) | 3.1 ± 0.31 (10) | 3.3 ± 0.17 (10) | 3.3 ± 0.29 (10) | 3.1 ± 0.26 (10) | 3.5 ± 0.61 (10) |  | 3.1 ± 0.38 (8) | 3.2 ± 0.26 (8) |
|  | IL-12p70 (ng/L) | M | 7.9 ± 1.25 (10) | 9.4 ± 2.58 (10) | 8.9 ± 2.14 (10) | 10.6 ± 1.91 (10) | 8.0 ± 1.48 (10) | 10.1 ± 1.44 (10) |  | 9.9 ± 2.39 (8) | 8.4 ± 1.06 (8) |
|  |  | F | 17.3 ± 3.98 (10) | 12.9 ± 2.07 (10) | 10.2 ± 2.30 (10) | 7.4 ± 1.34 (10)* | 9.9 ± 2.10 (10) | 10.6 ± 1.67 (10) |  | 13.0 ± 2.10 (8) | 10.3 ± 3.03 (8) |
|  | IL-13 (ng/L) | M | 3.0 ± 0.36 (10) | 4.5 ± 0.72 (10) | 5.1 ± 0.81 (10) | 4.4 ± 1.01 (10) | 3.6 ± 0.63 (10) | 4.8 ± 0.76 (10) |  | 4.0 ± 0.77 (8) | 4.9 ± 0.88 (8) |
|  |  | F | 3.3 ± 0.55 (10) | 5.5 ± 0.97 (10)* | 5.3 ± 1.05 (10) | 4.1 ± 0.72 (10) | 4.5 ± 0.72 (10) | 4.8 ± 0.69 (10) |  | 5.5 ± 1.25 (8) | 4.7 ± 1.52 (8) |
|  | IL-17a (ng/L) | M | 3.7 ± 0.47 (10) | 3.5 ± 0.18 (10) | 3.4 ± 0.30 (10) | 3.0 ± 0.27 (10) | 3.4 ± 0.20 (10) | 2.8 ± 0.41 (10) |  | 2.7 ± 0.39 (8) | 3.1 ± 0.34 (8) |
|  |  | F | 3.2 ± 0.45 (10) | 3.2 ± 0.25 (10) | 3.1 ± 0.33 (10) | 3.4 ± 0.25 (10) | 3.2 ± 0.35 (10) | 3.5 ± 0.47 (10) |  | 3.0 ± 0.31 (8) | 3.2 ± 0.29 (8) |
|  | IL-18 (ng/L) | M | 67.7 ± 9.64 (10) | 45.2 ± 5.14 (10) | 34.4 ± 10.30 (10)* | 40.8 ± 7.51 (10)* | 27.8 ± 2.92 (10)** | 49.2 ± 10.68 (10) |  | 39.5 ± 7.91 (8) | 48.4 ± 11.32 (8) |
|  |  | F | 59.2 ± 7.58 (10) | 51.6 ± 17.79 (10) | 38.6 ± 6.04 (10)* | 47.7 ± 8.07 (10) | 40.2 ± 6.37 (10) | 36.6 ± 8.08 (10) |  | 34.7 ± 4.29 (8) | 35.2 ± 5.24 (8) |
|  | IP-10 (ng/L) | M | 4.2 ± 1.29 (10) | 2.6 ± 0.62 (10) | 2.7 ± 1.36 (10) | 5.8 ± 3.12 (10) | 1.2 ± 0.34 (10)** | 1.8 ± 0.59 (10) |  | 2.3 ± 0.75 (8) | 3.3 ± 1.41 (8) |
|  |  | F | 5.0 ± 1.89 (10) | 1.2 ± 0.23 (10)*** | 1.8 ± 0.47 (10)* | 1.9 ± 0.35 (10)* | 2.0 ± 0.49 (10) | 1.4 ± 0.35 (10)** |  | 1.8 ± 0.38 (8) | 1.6 ± 0.44 (8) |
|  | Leptin (ng/L) | M | 7.1 ± 1.74 (10) | 6.1 ± 0.66 (10) | 6.3 ± 1.17 (10) | 5.6 ± 0.46 (10) | 7.1 ± 1.05 (10) | 8.5 ± 1.70 (10) |  | 14.5 ± 7.15 (8) | 9.4 ± 2.84 (8) |
|  |  | F | 8.5 ± 1.18 (10) | 8.0 ± 1.99 (10) | 5.1 ± 0.00 (10)* | 6.6 ± 0.86 (10) | 6.3 ± 0.62 (10) | 5.9 ± 0.79 (10) |  | 5.9 ± 0.84 (8) | 10.0 ± 2.63 (8) |
|  | LIX (ng/L) | M | 13.9 ± 1.88 (10) | 10.5 ± 0.00 (10) | 10.5 ± 0.00 (10) | 11.2 ± 0.74 (10) | 10.5 ± 0.00 (10) | 10.5 ± 0.00 (10) |  | 14.5 ± 4.07 (8) | 10.5 ± 0.00 (8) |
|  |  | F | 10.5 ± 0.00 (10) | 10.5 ± 0.00 (10) | 12.3 ± 1.34 (10) | 10.5 ± 0.00 (10) | 10.5 ± 0.00 (10) | 10.5 ± 0.00 (10) |  | 10.5 ± 0.00 (8) | 10.5 ± 0.00 (8) |
|  | MCP-1 (ng/L) | M | 109.1 ± 33.80 (10) | 100.7 ± 19.62 (10) | 99.0 ± 29.85 (10) | 43.7 ± 14.04 (10) | 78.1 ± 13.64 (10) | 60.4 ± 13.47 (10) |  | 124.1 ± 34.85 (8) | 81.6 ± 25.14 (8) |
|  |  | F | 74.1 ± 27.92 (10) | 68.9 ± 21.65 (10) | 103.5 ± 23.56 (10) | 93.5 ± 12.54 (10) | 68.8 ± 17.87 (10) | 103.5 ± 21.54 (10) |  | 83.5 ± 26.41 (8) | 68.9 ± 18.66 (8) |
|  | MIP-1a (ng/L) | M | 1.5 ± 0.17 (10) | 1.3 ± 0.03 (10) | 1.3 ± 0.05 (10) | 1.2 ± 0.03 (10) | 1.2 ± 0.06 (10) | 1.3 ± 0.12 (10) |  | 1.3 ± 0.08 (8) | 1.4 ± 0.16 (8) |
|  |  | F | 1.5 ± 0.13 (10) | 1.2 ± 0.01 (10) | 1.3 ± 0.08 (10) | 1.3 ± 0.05 (10) | 1.3 ± 0.14 (10) | 1.4 ± 0.15 (10) |  | 1.2 ± 0.07 (8) | 1.3 ± 0.10 (8) |
|  | MIP-2 (ng/L) | M | 16.5 ± 1.69 (10) | 12.9 ± 1.78 (10) | 11.7 ± 0.88 (10)* | 10.9 ± 0.89 (10)* | 12.7 ± 0.71 (10) | 10.1 ± 1.26 (10)** |  | 12.1 ± 0.61 (8) | 11.9 ± 0.44 (8) |
|  |  | F | 16.7 ± 2.13 (10) | 12.5 ± 1.73 (10) | 11.8 ± 0.52 (10)* | 10.4 ± 0.71 (10)* | 11.8 ± 0.91 (10)* | 12.0 ± 0.60 (10) |  | 12.1 ± 0.96 (8) | 11.7 ± 1.26 (8) |
|  | RANTES (ng/L) | M | 1.4 ± 0.13 (10) | 1.2 ± 0.16 (10) | 1.0 ± 0.16 (10) | 1.2 ± 0.16(10) | 1.1 ± 0.17 (10) | 1.1 ± 0.17 (10) |  | 1.2 ± 0.29 (8) | 1.1 ± 0.20 (8) |
|  |  | F | 1.5 ± 0.12 (10) | 1.0 ± 0.16 (10)* | 1.2 ± 0.17 (10) | 1.1 ± 0.16 (10) | 1.2 ± 0.20 (10) | 0.9 ± 0.14 (10)* |  | 1.0 ± 0.18 (8) | 1.1 ± 0.19 (8) |
|  | TNF-a (ng/L) | M | 1.0 ± 0.00 (10) | 1.0 ± 0.00 (10) | 1.0 ± 0.00 (10) | 1.0 ± 0.00 (10) | 1.0 ± 0.00 (10) | 1.0 ± 0.05 (10) |  | 1.0 ± 0.00 (8) | 1.1 ± 0.12 (8) |
|  |  | F | 1.0 ± 0.00 (10) | 1.0 ± 0.00 (10) | 1.0 ± 0.00 (10) | 1.0 ± 0.00 (10) | 1.0 ± 0.00 (10) | 1.0 ± 0.00 (10) |  | 1.0 ± 0.00 (8) | 1.0 ± 0.00 (8) |
|  | VEGF-a (ng/L) | M | 521.7 ± 88.04 (10) | 239.1 ± 24.56 (10)* | 256.1 ± 35.16 (10)* | 310.1 ± 63.53 (10) | 206.5 ± 24.94 (10)** | 192.3 ± 16.14 (10)** |  | 452.3 ± 81.64 (8)* | 307.6 ± 34.17 (8)* |
|  |  | F | 302.1 ± 82.59(10) | 173.8 ± 21.28 (10) | 468.5 ± 234.99 (10) | 243.4 ± 57.90 (10) | 182.1 ± 23.23 (10) | 215.9 ± 27.15 (10) |  | 224.3 ± 15.48 (8) | 270.6 ± 39.85 (8) |

*Remarks:*

*Relative cell count (%) = cell event/total cell events x 100*

*Absolute cell count = (relative cell count x total cell counts)/100*

**Supplemental Table 13** **Organ weights**

| **Type** | **Parameter** | **Sex** | **Exposed** | | | | | |  | **Recovery** | |
| --- | --- | --- | --- | --- | --- | --- | --- | --- | --- | --- | --- |
|  |  |  | **PBS** | **PG/VG + Nic** | **PG/VG + F-Med** | **PG/VG + Nic + F-Low** | **PG/VG + Nic + F-Med** | **PG/VG + Nic + F-High** |  | **PG/VG + Nic** | **PG/VG + Nic + F-High** |
| Absolute organ weight | Adrenal gland weight (g) | M | 0.05 ± 0.003 (10) | 0.07 ± 0.004 (10)** | 0.06 ± 0.005 (10) | 0.07 ± 0.004 (10)** | 0.07 ± 0.004 (10)* | 0.06 ± 0.003 (10) |  | 0.04 ± 0.002 (8)*** | 0.05 ± 0.004 (8)** |
|  |  | F | 0.06 ± 0.005 (10) | 0.10 ± 0.005 (10)*** | 0.07 ± 0.003 (10) | 0.11 ± 0.010 (10)*** | 0.09 ± 0.003 (10)** | 0.09 ± 0.003 (10)*** |  | 0.06 ± 0.005 (8)*** | 0.06 ± 0.004 (8)*** |
|  | Brain weight (g) | M | 2.22 ± 0.020 (10) | 2.16 ± 0.037 (10) | 2.21 ± 0.035 (10) | 2.17 ± 0.045 (10) | 2.13 ± 0.030 (10)* | 2.13 ± 0.036 (10) |  | 2.23 ± 0.033 (8) | 2.29 ± 0.030 (8)** |
|  |  | F | 2.05 ± 0.018 (10) | 2.03 ± 0.021 (10) | 2.00 ± 0.032 (10) | 2.04 ± 0.028 (10) | 2.02 ± 0.026 (10) | 2.08 ± 0.025 (10) |  | 2.04 ± 0.024 (8) | 2.05 ± 0.025 (8) |
|  | Epididymis weight (g) | M | 1.09 ± 0.024 (10) | 1.23 ± 0.079 (10) | 1.16 ± 0.085 (10) | 1.23 ± 0.053 (10)* | 1.15 ± 0.061 (10) | 1.12 ± 0.039 (10) |  | 1.52 ± 0.079 (8)* | 1.58 ± 0.044 (8)*** |
|  | Heart weight (g) | M | 1.33 ± 0.062 (10) | 1.33 ± 0.036 (10) | 1.33 ± 0.056 (10) | 1.30 ± 0.035 (10) | 1.28 ± 0.030 (10) | 1.22 ± 0.030 (10) |  | 1.60 ± 0.080 (8)* | 1.60 ± 0.074 (8)*** |
|  |  | F | 0.92 ± 0.029 (10) | 1.04 ± 0.035 (10)* | 0.94 ± 0.036 (10) | 1.09 ± 0.048 (10)* | 1.07 ± 0.030 (10)** | 1.05 ± 0.029 (10)** |  | 1.05 ± 0.035 (8) | 1.04 ± 0.024 (8) |
|  | Kidney weight (g) | M | 2.33 ± 0.086 (10) | 2.24 ± 0.060 (10) | 2.34 ± 0.167 (10) | 2.42 ± 0.093 (10) | 2.35 ± 0.059 (10) | 2.21 ± 0.061 (10) |  | 2.81 ± 0.137 (8)** | 2.81 ± 0.157 (8)** |
|  |  | F | 1.60 ± 0.061 (10) | 1.76 ± 0.071 (10) | 1.60 ± 0.061 (10) | 1.90 ± 0.078 (10)** | 1.82 ± 0.037 (10)** | 1.84 ± 0.031 (10)** |  | 1.86 ± 0.042 (8) | 1.77 ± 0.053 (8) |
|  | Liver weight (g) | M | 13.76 ± 0.513 (10) | 12.74 ± 0.459 (10) | 13.66 ± 1.062 (10) | 14.11 ± 0.355 (10) | 13.24 ± 0.206 (10) | 12.85 ± 0.537 (10) |  | 16.76 ± 0.835 (8)** | 16.47 ± 1.263 (8)* |
|  |  | F | 9.05 ± 0.293 (10) | 10.57 ± 0.387 (10)** | 8.38 ± 0.378 (10) | 11.86 ± 0.606 (10)** | 11.51 ± 0.564 (10)** | 10.85 ± 0.340 (10)*** |  | 9.55 ± 0.261 (8)* | 9.65 ± 0.302 (8)* |
|  | Lung, larynx, and trachea weight (g) | M | 1.60 ± 0.052 (10) | 1.57 ± 0.026 (10) | 1.57 ± 0.055 (10) | 1.59 ± 0.058 (10) | 1.65 ± 0.039 (10) | 1.58 ± 0.052 (10) |  | 1.86 ± 0.088 (8)* | 1.78 ± 0.058 (8)* |
|  |  | F | 1.28 ± 0.030 (10) | 1.36 ± 0.038 (10) | 1.34 ± 0.056 (10) | 1.43 ± 0.045 (10)* | 1.46 ± 0.069 (10)* | 1.39 ± 0.030 (10)* |  | 1.45 ± 0.054 (8) | 1.48 ± 0.041 (8) |
|  | Ovary weight (g) | F | 0.08 ± 0.007 (10) | 0.11 ± 0.007 (10)** | 0.09 ± 0.007 (10) | 0.13 ± 0.009 (10)** | 0.11 ± 0.009 (10) | 0.12 ± 0.009 (10)** |  | 0.09 ± 0.010 (8) | 0.10 ± 0.010 (8) |
|  | Spleen weight (g) | M | 0.70 ± 0.031 (10) | 0.61 ± 0.026 (10)* | 0.75 ± 0.062 (10) | 0.61 ± 0.025 (10)* | 0.59 ± 0.022 (10)** | 0.56 ± 0.036(10)** |  | 0.89 ± 0.039 (8)*** | 0.92 ± 0.044 (8)*** |
|  |  | F | 0.47 ± 0.022 (10) | 0.48 ± 0.021 (10) | 0.51 ± 0.015 (10) | 0.54 ± 0.027 (10) | 0.52 ± 0.021 (10) | 0.52 ± 0.020 (10) |  | 0.62 ± 0.042 (8)* | 0.66 ± 0.038 (8)* |
|  | Testes weight (g) | M | 2.96 ± 0.225 (10) | 3.30 ± 0.137 (10) | 3.06 ± 0.139 (10) | 3.30 ± 0.095 (10) | 3.13 ± 0.233 (10) | 3.37 ± 0.114 (10) |  | 3.80 ± 0.112 (8)* | 3.75 ± 0.090 (8)* |
|  | Thymus weight (g) | M | 0.20 ± 0.018 (10) | 0.15 ± 0.014 (10)* | 0.21 ± 0.031 (10) | 0.15 ± 0.006 (10)* | 0.14 ± 0.007 (10)** | 0.14 ± 0.013 (10)* |  | 0.22 ± 0.017 (8)** | 0.25 ± 0.032 (8)* |
|  |  | F | 0.17 ± 0.017 (10) | 0.15 ± 0.010 (10) | 0.20 ± 0.019 (10) | 0.13 ± 0.010 (10) | 0.14 ± 0.012 (10) | 0.14 ± 0.008 (10) |  | 0.21 ± 0.016 (8)** | 0.21 ± 0.023 (8)* |
|  | Thyroid and parathyroid weight (g) | M | 0.02 ± 0.002 (10) | 0.03 ± 0.004 (10) | 0.02 ± 0.002 (10) | 0.02 ± 0.002 (10) | 0.02 ± 0.002 (10) | 0.03 ± 0.004 (10) |  | 0.03 ± 0.003 (8) | 0.03 ± 0.003 (8) |
|  |  | F | 0.02 ± 0.001 (10) | 0.03 ± 0.002 (10)* | 0.02 ± 0.002 (10) | 0.02 ± 0.002 (10) | 0.02 ± 0.001 (10)* | 0.02 ± 0.002 (10)* |  | 0.03 ± 0.003 (8) | 0.03 ± 0.002 (8) |
|  | Uterus, and cervix weight (g) | F | 0.52 ± 0.033 (10) | 0.60 ± 0.106 (10) | 0.77 ± 0.091 (10)* | 0.48 ± 0.040 (10) | 0.41 ± 0.035 (10)* | 0.57 ± 0.096(10) |  | 0.96 ± 0.162(8) | 0.90 ± 0.131 (8)* |
| Weight relative to body weight | Adrenal gland weight | M | 0.013 ± 75E-5 (10) | 0.019 ± 0.001 (10)*** | 0.014 ± 0.001 (10) | 0.017 ± 67E-5 (10)*** | 0.018 ± 0.001 (10)** | 0.016 ± 67E-5 (10)** |  | 0.009 ± 35E-5 (8)*** | 0.009 ± 88E-5 (8)*** |
|  |  | F | 0.027 ± 0.002 (10) | 0.036 ± 0.002 (10)** | 0.031 ± 0.001 (10) | 0.038 ± 0.003 (10)** | 0.031 ± 0.001 (10) | 0.033 ± 91E-5 (10)* |  | 0.022 ± 0.002 (8)*** | 0.019 ± 0.001 (8)*** |
|  | Brain weight | M | 0.54 ± 0.012 (10) | 0.577 ± 0.011 (10)* | 0.555 ± 0.024 (10) | 0.559 ± 0.011 (10) | 0.571 ± 0.011 (10) | 0.577 ± 0.013 (10) |  | 0.458 ± 0.015 (8)*** | 0.465 ± 0.022 (8)*** |
|  |  | F | 0.858 ± 0.018 (10) | 0.767 ± 0.027 (10)* | 0.831 ± 0.02 (10) | 0.745 ± 0.021 (10)*** | 0.719 ± 0.021 (10)*** | 0.763 ± 0.021 (10)** |  | 0.708 ± 0.016 (8) | 0.694 ± 0.018 (8)* |
|  | Epididymis weight | M | 0.267 ± 0.011 (10) | 0.327 ± 0.021 (10)* | 0.285 ± 0.014 (10) | 0.321 ± 0.02 (10)* | 0.306 ± 0.014 (10)* | 0.303 ± 0.012 (10)* |  | 0.31 ± 0.011 (8) | 0.321 ± 0.016 (8) |
|  | Heart weight | M | 0.322 ± 0.008 (10) | 0.356 ± 0.01 (10)* | 0.33 ± 0.007 (10) | 0.334 ± 0.008 (10) | 0.341 ± 0.007 (10) | 0.329 ± 0.006 (10) |  | 0.328 ± 0.017 (8) | 0.325 ± 0.019 (8) |
|  |  | F | 0.385 ± 0.01 (10) | 0.389 ± 0.012 (10) | 0.386 ± 0.008 (10) | 0.393 ± 0.008 (10) | 0.379 ± 0.008 (10) | 0.384 ± 0.011 (10) |  | 0.362 ± 0.008 (8) | 0.35 ± 0.008 (8)* |
|  | Kidney weight | M | 0.566 ± 0.016 (10) | 0.597 ± 0.012 (10) | 0.582 ± 0.037 (10) | 0.622 ± 0.015 (10)* | 0.627 ± 0.015 (10)* | 0.597 ± 0.015 (10) |  | 0.573 ± 0.016 (8) | 0.562 ± 0.016 (8) |
|  |  | F | 0.665 ± 0.017 (10) | 0.658 ± 0.024 (10) | 0.661 ± 0.018 (10) | 0.689 ± 0.016 (10) | 0.647 ± 0.013 (10) | 0.675 ± 0.014 (10) |  | 0.646 ± 0.023 (8) | 0.596 ± 0.014 (8)*** |
|  | Liver weight | M | 3.334 ± 0.074 (10) | 3.386 ± 0.09 (10) | 3.34 ± 0.108 (10) | 3.632 ± 0.087 (10)* | 3.54 ± 0.062 (10)* | 3.454 ± 0.076 (10) |  | 3.409 ± 0.068 (8) | 3.272 ± 0.101 (8) |
|  |  | F | 3.776 ± 0.077 (10) | 3.946 ± 0.076 (10) | 3.449 ± 0.106 (10)* | 4.286 ± 0.123 (10)** | 4.064 ± 0.146 (10) | 3.957 ± 0.051 (10) |  | 3.314 ± 0.097 (8)*** | 3.251 ± 0.071 (8)*** |
|  | Lung, larynx, and trachea weight | M | 0.388 ± 0.006 (10) | 0.417 ± 0.008 (10)** | 0.389 ± 0.009 (10) | 0.407 ± 0.006 (10)* | 0.441 ± 0.009 (10)*** | 0.427 ± 0.011 (10)** |  | 0.382 ± 0.02 (8) | 0.36 ± 0.012 (8)*** |
|  |  | F | 0.537 ± 0.01 (10) | 0.508 ± 0.01 (10) | 0.555 ± 0.018 (10) | 0.518 ± 0.01 (10) | 0.515 ± 0.021 (10) | 0.507 ± 0.012 (10) |  | 0.501 ± 0.014 (8) | 0.498 ± 0.01 (8) |
|  | Ovary weight | F | 0.034 ± 0.003 (10) | 0.041 ± 0.003 (10) | 0.036 ± 0.002 (10) | 0.046 ± 0.003 (10)** | 0.037 ± 0.003 (10) | 0.043 ± 0.003 (10)* |  | 0.031 ± 0.003 (8)* | 0.034 ± 0.003 (8) |
|  | Spleen weight | M | 0.17 ± 0.006 (10) | 0.163 ± 0.007 (10) | 0.184 ± 0.008 (10) | 0.157 ± 0.006 (10) | 0.157 ± 0.005 (10) | 0.151 ± 0.008 (10) |  | 0.183 ± 0.009 (8) | 0.185 ± 0.007 (8)** |
|  |  | F | 0.198 ± 0.007 (10) | 0.182 ± 0.007 (10) | 0.212 ± 0.005 (10) | 0.195 ± 0.009 (10) | 0.184 ± 0.007 (10) | 0.192 ± 0.008 (10) |  | 0.214 ± 0.014 (8) | 0.221 ± 0.012 (8) |
|  | Testes weight | M | 0.729 ± 0.06 (10) | 0.883 ± 0.042 (10) | 0.76 ± 0.032 (10) | 0.85 ± 0.031 (10) | 0.834 ± 0.059 (10) | 0.915 ± 0.041 (10)* |  | 0.778 ± 0.021 (8)* | 0.76 ± 0.029 (8)** |
|  | Thymus weight | M | 0.049 ± 0.004 (10) | 0.039 ± 0.004 (10) | 0.05 ± 0.005 (10) | 0.039 ± 0.002 (10) | 0.037 ± 0.002 (10)* | 0.038 ± 0.003 (10) |  | 0.046 ± 0.004 (8) | 0.049 ± 0.005 (8) |
|  |  | F | 0.071 ± 0.007 (10) | 0.054 ± 0.003 (10)* | 0.083 ± 0.006 (10) | 0.049 ± 0.004 (10)* | 0.051 ± 0.004 (10)* | 0.051 ± 0.003 (10)* |  | 0.074 ± 0.005 (8)** | 0.071 ± 0.006 (8)* |
|  | Thyroid and parathyroid weight | M | 0.006 ± 38E-5 (10) | 0.007 ± 82E-5 (10) | 0.006 ± 42E-5 (10) | 0.006 ± 44E-5 (10) | 0.006 ± 56E-5 (10) | 0.008 ± 0.001 (10) |  | 0.006 ± 35E-5 (8) | 0.006 ± 56E-5 (8) |
|  |  | F | 0.008 ± 63E-5 (10) | 0.009 ± 72E-5 (10) | 0.01 ± 59E-5 (10)* | 0.009 ± 93E-5 (10) | 0.008 ± 56E-5 (10) | 0.009 ± 9E-4 (10) |  | 0.009 ± 73E-5 (8) | 0.009 ± 58E-5 (8) |
|  | Uterus, and cervix weight | F | 0.221 ± 0.017 (10) | 0.224 ± 0.039 (10) | 0.319 ± 0.039 (10)* | 0.174 ± 0.014 (10)* | 0.147 ± 0.014 (10)** | 0.215 ± 0.041 (10) |  | 0.331 ± 0.055 (8) | 0.303 ± 0.044 (8) |
| Weight relative to brain weight | Adrenal gland weight | M | 2.4 ± 0.13 (10) | 3.3 ± 0.25 (10)** | 2.5 ± 0.23 (10) | 3.0 ± 0.14 (10)** | 3.1 ± 0.22 (10)* | 2.9 ± 0.17 (10)* |  | 2.0 ± 0.10 (8)*** | 2.0 ± 0.17 (8)** |
|  |  | F | 3.1 ± 0.26 (10) | 4.8 ± 0.22 (10)*** | 3.7 ± 0.15 (10) | 5.2 ± 0.43 (10)*** | 4.3 ± 0.17 (10)** | 4.4 ± 0.16 (10)*** |  | 3.1 ± 0.29 (8)*** | 2.8 ± 0.16 (8)*** |
|  | Epididymis weight | M | 49.4 ± 1.31 (10) | 56.6 ± 3.26 (10) | 52.0 ± 3.17 (10) | 57.2 ± 3.01 (10)* | 54.0 ± 3.04 (10) | 52.5 ± 1.90 (10) |  | 68.1 ± 2.84 (8)* | 69.1 ± 1.66 (8)*** |
|  | Heart weight | M | 60.1 ± 2.46 (10) | 61.9 ± 2.22 (10) | 60.2 ± 2.17 (10) | 59.9 ± 1.66 (10) | 59.9 ± 1.45 (10) | 57.2 ± 1.21 (10) |  | 71.5 ± 2.73 (8)* | 70.1 ± 3.11 (8)** |
|  |  | F | 45.1 ± 1.48 (10) | 51.1 ± 2.05 (10)* | 46.8 ± 1.59 (10) | 53.3 ± 2.27 (10)** | 53.1 ± 1.77 (10)** | 50.6 ± 1.60 (10)* |  | 51.3 ± 1.82 (8) | 50.6 ± 1.00 (8) |
|  | Kidney weight | M | 105.1 ± 3.44 (10) | 103.7 ± 2.10 (10) | 105.6 ± 7.05 (10) | 111.3 ± 2.54 (10) | 110.2 ± 3.28 (10) | 103.7 ± 2.66 (10) |  | 126.0 ± 5.31 (8)** | 122.5 ± 5.95 (8)* |
|  |  | F | 78.0 ± 2.98 (10) | 86.5 ± 3.75 (10) | 79.9 ± 2.48 (10) | 93.1 ± 3.36 (10)** | 90.3 ± 2.08 (10)** | 88.8 ± 1.52 (10)* |  | 91.3 ± 2.65 (8) | 86.1 ± 2.40 (8) |
|  | Liver weight | M | 619.8 ± 19.35 (10) | 590.3 ± 23.15 (10) | 615.6 ± 41.45 (10) | 650.5 ± 13.90 (10) | 622.3 ± 14.58 (10) | 603.2 ± 25.45 (10) |  | 751.4 ± 33.89 (8)** | 716.9 ± 47.17 (8) |
|  |  | F | 442.8 ± 15.26 (10) | 521.1 ± 22.02 (10)** | 417.2 ± 15.37 (10) | 580.5 ± 26.56 (10)*** | 570.3 ± 29.02 (10)** | 522.8 ± 17.59 (10)** |  | 468.3 ± 11.05 (8) | 471.2 ± 16.82 (8) |
|  | Lung, larynx, and trachea weight | M | 72.2 ± 2.11 (10) | 72.5 ± 1.61 (10) | 70.8 ± 2.12 (10) | 73.1 ± 1.35 (10) | 77.5 ± 2.24 (10) | 74.2 ± 1.74 (10) |  | 83.7 ± 4.22 (8)* | 77.9 ± 2.25 (8) |
|  |  | F | 62.7 ± 1.16 (10) | 66.8 ± 1.98 (10)* | 67.0 ± 2.41 (10) | 69.9 ± 2.17 (10)* | 72.4 ± 4.22 (10) | 66.8 ± 1.94 (10) |  | 71.1 ± 2.98 (8) | 72.0 ± 1.94 (8) |
|  | Ovary weight | F | 4.0 ± 0.35 (10) | 5.4 ± 0.35 (10)** | 4.4 ± 0.32 (10) | 6.2 ± 0.41 (10)*** | 5.3 ± 0.46 (10)* | 5.6 ± 0.38 (10)** |  | 4.4 ± 0.53 (8) | 5.0 ± 0.45 (8) |
|  | Spleen weight | M | 31.6 ± 1.30 (10) | 28.3 ± 1.12 (10) | 33.8 ± 2.45 (10) | 28.2 ± 1.17 (10) | 27.6 ± 1.00 (10)* | 26.3 ± 1.46 (10)* |  | 40.0 ± 1.64 (8)*** | 40.1 ± 1.61 (8)*** |
|  |  | F | 23.2 ± 0.95 (10) | 23.8 ± 1.06 (10) | 25.5 ± 0.57 (10)* | 26.2 ± 1.23 (10) | 25.7 ± 1.13 (10) | 25.2 ± 1.00 (10) |  | 30.4 ± 2.15 (8)* | 31.9 ± 1.68 (8)** |
|  | Testes weight | M | 133.9 ± 10.21 (10) | 153.3 ± 7.18 (10) | 137.9 ± 4.98 (10) | 151.9 ± 3.93 (10) | 147.6 ± 11.73 (10) | 158.6 ± 5.99 (10) |  | 170.5 ± 4.47 (8) | 164.2 ± 4.37 (8) |
|  | Thymus weight | M | 9.1 ± 0.78 (10) | 6.8 ± 0.59 (10)* | 9.4 ± 1.29 (10) | 7.0 ± 0.36 (10)* | 6.5 ± 0.33 (10)* | 6.6 ± 0.50 (10)* |  | 9.9 ± 0.76 (8)** | 10.9 ± 1.41 (8)* |
|  |  | F | 8.4 ± 0.84 (10) | 7.2 ± 0.53 (10) | 10.1 ± 0.80 (10) | 6.5 ± 0.45 (10) | 7.1 ± 0.59 (10) | 6.7 ± 0.41 (10) |  | 10.5 ± 0.72 (8)** | 10.4 ± 1.22 (8)* |
|  | Thyroid and parathyroid weight | M | 1.1 ± 0.09 (10) | 1.3 ± 0.16 (10) | 1.1 ± 0.09 (10) | 1.1 ± 0.07 (10) | 1.0 ± 0.09 (10) | 1.3 ± 0.16 (10) |  | 1.3 ± 0.10 (8) | 1.4 ± 0.15 (8) |
|  |  | F | 0.9 ± 0.07 (10) | 1.2 ± 0.11 (10)* | 1.2 ± 0.08 (10)* | 1.2 ± 0.12 (10) | 1.1 ± 0.05 (10)* | 1.2 ± 0.11 (10)* |  | 1.2 ± 0.13 (8) | 1.2 ± 0.10 (8) |
|  | Uterus, and cervix weight | F | 25.6 ± 1.57 (10) | 29.3 ± 5.10 (10) | 38.4 ± 4.61 (10)* | 23.4 ± 1.84 (10) | 20.3 ± 1.73 (10)* | 27.6 ± 4.71 (10) |  | 47.1 ± 7.85(8)* | 44.1 ± 6.82(8)* |

**Supplemental Table 14** **Histopathological findings, all organs**

| **Organ** | **Observation** | **Sex** | **Exposed** | | | | | |  | **Recovery** | |
| --- | --- | --- | --- | --- | --- | --- | --- | --- | --- | --- | --- |
|  |  |  | **PBS** | **PG/VG + Nic** | **PG/VG + F-Med** | **PG/VG + Nic + F-Low** | **PG/VG + Nic + F-Med** | **PG/VG + Nic + F-High** |  | **PG/VG + Nic** | **PG/VG + Nic + F-High** |
| Larynx | Any microscopic findings (incidence) | M | 4/10 | 9/10 | 2/10 | 10/10* | 10/10* | 9/9* |  | 2/8* | 2/8** |
|  |  | F | 6/10 | 10/10 | 5/10 | 10/10 | 9/10 | 10/10 |  | 2/8** | 2/8** |
|  | Infiltration (score) | M | 0.0 ± 0.00 (10) | 0.3 ± 0.21 (10) | 0.0 ± 0.00 (10) | 0.3 ± 0.21 (10) | 1.0 ± 0.33 (10)** | 0.2 ± 0.15 (9) |  | 0.3 ± 0.16 (8) | 0.5 ± 0.38 (8) |
|  |  | F | 0.1 ± 0.10 (10) | 0.1 ± 0.10 (10) | 0.0 ± 0.00 (10) | 1.0 ± 0.33 (10)* | 0.1 ± 0.10 (10) | 0.0 ± 0.00 (10) |  | 0.3 ± 0.16 (8) | 0.4 ± 0.26 (8) |
|  | Metaplasia (score) | M | 0.4 ± 0.16 (10) | 0.9 ± 0.10 (10)* | 0.2 ± 0.13 (10) | 1.1 ± 0.10 (10)** | 1.3 ± 0.15 (10)** | 1.0 ± 0.00 (9)** |  | 0.0 ± 0.00 (8)*** | 0.0 ± 0.00 (8)*** |
|  |  | F | 0.5 ± 0.17 (10) | 1.0 ± 0.00 (10)* | 0.5 ± 0.17 (10) | 1.1 ± 0.10 (10)* | 0.9 ± 0.10 (10) | 1.0 ± 0.00 (10)* |  | 0.0 ± 0.00 (8)*** | 0.0 ± 0.00 (8)*** |
| Lung | Any microscopic findings (incidence) | M | 2/10 | 2/10 | 5/10 | 4/10 | 0/10 | 5/10 |  | 5/8 | 2/8 |
|  |  | F | 2/10 | 4/10 | 5/10 | 4/10 | 0/10 | 5/10 |  | 2/8 | 1/8 |
|  | Infiltration (score) | M | 0.1 ± 0.10 (10) | 0.2 ± 0.13 (10) | 0.2 ± 0.13 (10) | 0.1 ± 0.10 (10) | 0.0 ± 0.00 (10) | 0.2 ± 0.13 (10) |  | 0.5 ± 0.27 (8) | 0.1 ± 0.13 (8) |
|  |  | F | 0.1 ± 0.10 (10) | 0.3 ± 0.15 (10) | 0.4 ± 0.16 (10) | 0.2 ± 0.13 (10) | 0.0 ± 0.00 (10) | 0.0 ± 0.00 (10) |  | 0.3 ± 0.16 (8) | 0.0 ± 0.00 (8) |
|  | Metaplasia (score) | M | 0.1 ± 0.10 (10) | 0.0 ± 0.00 (10) | 0.0 ± 0.00 (10) | 0.0 ± 0.00 (10) | 0.0 ± 0.00 (10) | 0.0 ± 0.00 (10) |  | 0.0 ± 0.00 (8) | 0.0 ± 0.00 (8) |
|  |  | F | 0.0 ± 0.00 (10) | 0.0 ± 0.00 (10) | 0.0 ± 0.00 (10) | 0.0 ± 0.00 (10) | 0.0 ± 0.00 (10) | 0.0 ± 0.00 (10) |  | 0.0 ± 0.00 (8) | 0.0 ± 0.00 (8) |
|  | Aggregation (score) | M | 0.1 ± 0.10 (10) | 0.1 ± 0.10 (10) | 0.2 ± 0.13 (10) | 0.3 ± 0.15 (10) | 0.0 ± 0.00 (10) | 0.3 ± 0.15 (10) |  | 0.1 ± 0.13 (8) | 0.1 ± 0.13 (8) |
|  |  | F | 0.1 ± 0.10 (10) | 0.2 ± 0.13 (10) | 0.1 ± 0.10 (10) | 0.2 ± 0.13 (10) | 0.0 ± 0.00 (10) | 0.2 ± 0.13 (10) |  | 0.0 ± 0.00 (8) | 0.1 ± 0.13 (8) |
|  | Fibrosis (score) | M | 0.0 ± 0.00 (10) | 0.0 ± 0.00 (10) | 0.0 ± 0.00 (10) | 0.0 ± 0.00 (10) | 0.0 ± 0.00 (10) | 0.0 ± 0.00 (10) |  | 0.0 ± 0.00 (8) | 0.0 ± 0.00 (8) |
|  |  | F | 0.0 ± 0.00 (10) | 0.0 ± 0.00 (10) | 0.0 ± 0.00 (10) | 0.0 ± 0.00 (10) | 0.0 ± 0.00 (10) | 0.1 ± 0.10 (10) |  | 0.0 ± 0.00 (8) | 0.0 ± 0.00 (8) |
|  | Hemorrhage (score) | M | 0.0 ± 0.00 (10) | 0.0 ± 0.00 (10) | 0.3 ± 0.15 (10) | 0.1 ± 0.10 (10) | 0.0 ± 0.00 (10) | 0.1 ± 0.10 (10) |  | 0.4 ± 0.38 (8) | 0.0 ± 0.00 (8) |
|  |  | F | 0.0 ± 0.00 (10) | 0.0 ± 0.00 (10) | 0.0 ± 0.00 (10) | 0.0 ± 0.00 (10) | 0.0 ± 0.00 (10) | 0.3 ± 0.15 (10) |  | 0.0 ± 0.00 (8) | 0.0 ± 0.00 (8) |
| Nose level 1 | Any microscopic findings (incidence) | M | 1/10 | 1/10 | 1/10 | 0/10 | 0/10 | 1/10 |  | 0/8 | 1/8 |
|  |  | F | 0/10 | 0/10 | 0/10 | 0/10 | 0/10 | 0/10 |  | 2/8 | 1/8 |
|  | Infiltration (score) | M | 0.0 ± 0.00 (10) | 0.0 ± 0.00 (10) | 0.0 ± 0.00 (10) | 0.0 ± 0.00 (10) | 0.0 ± 0.00 (10) | 0.0 ± 0.00 (10) |  | 0.0 ± 0.00 (8) | 0.0 ± 0.00 (8) |
|  |  | F | 0.0 ± 0.00 (10) | 0.0 ± 0.00 (10) | 0.0 ± 0.00 (10) | 0.0 ± 0.00 (10) | 0.0 ± 0.00 (10) | 0.0 ± 0.00 (10) |  | 0.3 ± 0.16 (8) | 0.1 ± 0.13 (8) |
|  | Ectasia (score) | M | 0.0 ± 0.00 (10) | 0.1 ± 0.10 (10) | 0.0 ± 0.00 (10) | 0.0 ± 0.00 (10) | 0.0 ± 0.00 (10) | 0.1 ± 0.10 (10) |  | 0.0 ± 0.00 (8) | 0.0 ± 0.00 (8) |
|  |  | F | 0.0 ± 0.00 (10) | 0.0 ± 0.00 (10) | 0.0 ± 0.00 (10) | 0.0 ± 0.00 (10) | 0.0 ± 0.00 (10) | 0.0 ± 0.00 (10) |  | 0.0 ± 0.00 (8) | 0.0 ± 0.00 (8) |
|  | Hyperplasia (score) | M | 0.1 ± 0.10 (10) | 0.0 ± 0.00 (10) | 0.1 ± 0.10 (10) | 0.0 ± 0.00 (10) | 0.0 ± 0.00 (10) | 0.0 ± 0.00 (10) |  | 0.0 ± 0.00 (8) | 0.1 ± 0.13 (8) |
|  |  | F | 0.0 ± 0.00 (10) | 0.0 ± 0.00 (10) | 0.0 ± 0.00 (10) | 0.0 ± 0.00 (10) | 0.0 ± 0.00 (10) | 0.0 ± 0.00 (10) |  | 0.1 ± 0.13 (8) | 0.0 ± 0.00 (8) |
| Nose level 2 | Any microscopic findings (incidence) | M | 2/10 | 1/10 | 0/10 | 0/10 | 0/10 | 1/10 |  | 0/8 | 1/7 |
|  |  | F | 4/10 | 1/10 | 0/10 | 0/10 | 0/10 | 0/10 |  | 0/8 | 0/8 |
|  | Infiltration (score) | M | 0.0 ± 0.00 (10) | 0.0 ± 0.00 (10) | 0.0 ± 0.00 (10) | 0.0 ± 0.00 (10) | 0.0 ± 0.00 (10) | 0.0 ± 0.00 (10) |  | 0.0 ± 0.00 (8) | 0.0 ± 0.00 (7) |
|  |  | F | 0.0 ± 0.00 (10) | 0.2 ± 0.20 (10) | 0.0 ± 0.00 (10) | 0.0 ± 0.00 (10) | 0.0 ± 0.00 (10) | 0.0 ± 0.00 (10) |  | 0.0 ± 0.00 (8) | 0.0 ± 0.00 (8) |
|  | Ectasia (score) | M | 0.0 ± 0.00 (10) | 0.1 ± 0.10 (10) | 0.0 ± 0.00 (10) | 0.0 ± 0.00 (10) | 0.0 ± 0.00 (10) | 0.1 ± 0.10 (10) |  | 0.0 ± 0.00 (8) | 0.1 ± 0.14 (7) |
|  |  | F | 0.0 ± 0.00 (10) | 0.0 ± 0.00 (10) | 0.0 ± 0.00 (10) | 0.0 ± 0.00 (10) | 0.0 ± 0.00 (10) | 0.0 ± 0.00 (10) |  | 0.0 ± 0.00 (8) | 0.0 ± 0.00 (8) |
|  | Eosinophilic Globules (score) | M | 0.4 ± 0.27 (10) | 0.0 ± 0.00 (10) | 0.0 ± 0.00 (10) | 0.0 ± 0.00 (10) | 0.0 ± 0.00 (10) | 0.0 ± 0.00 (10) |  | 0.0 ± 0.00 (8) | 0.0 ± 0.00 (7) |
|  |  | F | 0.8 ± 0.33 (10) | 0.0 ± 0.00 (10)* | 0.0 ± 0.00 (10)* | 0.0 ± 0.00 (10)* | 0.0 ± 0.00 (10)* | 0.0 ± 0.00 (10)* |  | 0.0 ± 0.00 (8) | 0.0 ± 0.00 (8) |
| Nose level 3 | Any microscopic findings (incidence) | M | 2/10 | 0/10 | 0/10 | 0/10 | 0/10 | 1/10 |  | 0/8 | 0/8 |
|  |  | F | 4/10 | 0/10 | 0/10 | 0/10 | 0/10 | 0/10 |  | 0/8 | 0/8 |
|  | Ectasia (score) | M | 0.0 ± 0.00 (10) | 0.0 ± 0.00 (10) | 0.0 ± 0.00 (10) | 0.0 ± 0.00 (10) | 0.0 ± 0.00 (10) | 0.1 ± 0.10 (10) |  | 0.0 ± 0.00 (8) | 0.0 ± 0.00 (8) |
|  |  | F | 0.0 ± 0.00 (10) | 0.0 ± 0.00 (10) | 0.0 ± 0.00 (10) | 0.0 ± 0.00 (10) | 0.0 ± 0.00 (10) | 0.0 ± 0.00 (10) |  | 0.0 ± 0.00 (8) | 0.0 ± 0.00 (8) |
|  | Eosinophilic Globules (score) | M | 0.4 ± 0.27 (10) | 0.0 ± 0.00 (10) | 0.0 ± 0.00 (10) | 0.0 ± 0.00 (10) | 0.0 ± 0.00 (10) | 0.0 ± 0.00 (10) |  | 0.0 ± 0.00 (8) | 0.0 ± 0.00 (8) |
|  |  | F | 0.8 ± 0.33 (10) | 0.0 ± 0.00 (10)* | 0.0 ± 0.00 (10)* | 0.0 ± 0.00 (10)* | 0.0 ± 0.00 (10)* | 0.0 ± 0.00 (10)* |  | 0.0 ± 0.00 (8) | 0.0 ± 0.00 (8) |
| Nose level 4 | Any microscopic findings (incidence) | M | 0/10 | 0/10 | 0/10 | 0/10 | 0/10 | 0/10 |  | 0/8 | 0/8 |
|  |  | F | 0/10 | 0/10 | 0/10 | 0/10 | 0/10 | 0/10 |  | 0/8 | 0/8 |
| Trachea | Any microscopic findings (incidence) | M | 0/10 | 0/10 | 2/10 | 2/10 | 0/10 | 1/10 |  | 0/8 | 1/8 |
|  |  | F | 0/10 | 1/10 | 1/10 | 1/10 | 0/10 | 2/10 |  | 0/8 | 0/8 |
|  | Infiltration (score) | M | 0.0 ± 0.00 (10) | 0.0 ± 0.00 (10) | 0.2 ± 0.13 (10) | 0.2 ± 0.13 (10) | 0.0 ± 0.00 (10) | 0.0 ± 0.00 (10) |  | 0.0 ± 0.00 (8) | 0.1 ± 0.13 (8) |
|  |  | F | 0.0 ± 0.00 (10) | 0.1 ± 0.10 (10) | 0.1 ± 0.10 (10) | 0.1 ± 0.10 (10) | 0.0 ± 0.00 (10) | 0.1 ± 0.10 (10) |  | 0.0 ± 0.00 (8) | 0.0 ± 0.00 (8) |
|  | Cyst (score) | M | 0.0 ± 0.00 (10) | 0.0 ± 0.00 (10) | 0.0 ± 0.00 (10) | 0.0 ± 0.00 (10) | 0.0 ± 0.00 (10) | 0.1 ± 0.10 (10) |  | 0.0 ± 0.00 (8) | 0.0 ± 0.00 (8) |
|  |  | F | 0.0 ± 0.00 (10) | 0.0 ± 0.00 (10) | 0.0 ± 0.00 (10) | 0.0 ± 0.00 (10) | 0.0 ± 0.00 (10) | 0.0 ± 0.00 (10) |  | 0.0 ± 0.00 (8) | 0.0 ± 0.00 (8) |
|  | Exudate (score) | M | 0.0 ± 0.00 (10) | 0.0 ± 0.00 (10) | 0.0 ± 0.00 (10) | 0.0 ± 0.00 (10) | 0.0 ± 0.00 (10) | 0.0 ± 0.00 (10) |  | 0.0 ± 0.00 (8) | 0.0 ± 0.00 (8) |
|  |  | F | 0.0 ± 0.00 (10) | 0.0 ± 0.00 (10) | 0.0 ± 0.00 (10) | 0.0 ± 0.00 (10) | 0.0 ± 0.00 (10) | 0.1 ± 0.10 (10) |  | 0.0 ± 0.00 (8) | 0.0 ± 0.00 (8) |
|  | Ulceration (score) | M | 0.0 ± 0.00 (10) | 0.0 ± 0.00 (10) | 0.0 ± 0.00 (10) | 0.0 ± 0.00 (10) | 0.0 ± 0.00 (10) | 0.0 ± 0.00 (10) |  | 0.0 ± 0.00 (8) | 0.0 ± 0.00 (8) |
|  |  | F | 0.0 ± 0.00 (10) | 0.0 ± 0.00 (10) | 0.0 ± 0.00 (10) | 0.0 ± 0.00 (10) | 0.0 ± 0.00 (10) | 0.2 ± 0.20 (10) |  | 0.0 ± 0.00 (8) | 0.0 ± 0.00 (8) |
| Artery, aorta | Any microscopic findings (incidence) | M | 0/10 | 0/10 | 0/10 | 0/10 | 0/10 | 0/10 |  | 0/8 | 0/8 |
|  |  | F | 0/10 | 0/10 | 0/10 | 0/10 | 0/10 | 0/10 |  | 0/8 | 0/8 |
| Bone, sternum | Any microscopic findings (incidence) | M | 0/10 | 0/9 | 0/10 | 0/10 | 0/10 | 0/10 |  | 0/8 | 0/8 |
|  |  | F | 0/10 | 0/10 | 0/10 | 0/10 | 0/10 | 0/10 |  | 0/8 | 0/8 |
| Brain | Any microscopic findings (incidence) | M | 0/10 | 0/10 | 0/10 | 0/10 | 0/10 | 0/10 |  | 0/8 | 0/8 |
|  |  | F | 0/10 | 0/10 | 0/10 | 0/10 | 0/10 | 0/10 |  | 0/8 | 0/8 |
| Epididymis | Any microscopic findings (incidence) | M | 6/10 | 5/10 | 7/10 | 4/10 | 2/10 | 4/10 |  | 0/8* | 1/8 |
|  | Cell Debris (score) | M | 1.0 ± 0.33 (10) | 0.6 ± 0.22 (10) | 1.5 ± 0.37 (10) | 0.4 ± 0.16 (10) | 0.7 ± 0.47 (10) | 0.3 ± 0.15 (10) |  | 0.0 ± 0.00 (8)* | 0.1 ± 0.13 (8) |
|  | Infiltration (score) | M | 0.0 ± 0.00 (10) | 0.0 ± 0.00 (10) | 0.0 ± 0.00 (10) | 0.0 ± 0.00 (10) | 0.0 ± 0.00 (10) | 0.1 ± 0.10 (10) |  | 0.0 ± 0.00 (8) | 0.0 ± 0.00 (8) |
|  | Sperm Granuloma (score) | M | 0.0 ± 0.00 (10) | 0.0 ± 0.00 (10) | 0.5 ± 0.34 (10) | 0.0 ± 0.00 (10) | 0.0 ± 0.00 (10) | 0.0 ± 0.00 (10) |  | 0.0 ± 0.00 (8) | 0.0 ± 0.00 (8) |
| Esophagus | Any microscopic findings (incidence) | M | 0/10 | 0/10 | 0/10 | 0/10 | 0/10 | 0/10 |  | 0/8 | 0/8 |
|  |  | F | 0/10 | 0/10 | 0/10 | 0/10 | 0/10 | 0/10 |  | 0/8 | 0/8 |
| Eye | Any microscopic findings (incidence) | M | 0/10 | 0/10 | 0/10 | 0/10 | 0/10 | 0/10 |  | 0/8 | 0/8 |
|  |  | F | 0/10 | 0/10 | 0/10 | 0/10 | 0/10 | 0/10 |  | 0/8 | 0/8 |
| Gland, adrenal | Any microscopic findings (incidence) | M | 1/10 | 5/10 | 1/10 | 6/10 | 6/10 | 8/10** |  | 0/8* | 0/8** |
|  |  | F | 0/10 | 6/10* | 0/10 | 1/10 | 1/10 | 8/10** |  | 0/8* | 0/8 |
|  | Hemorrhage (score) | M | 0.0 ± 0.00 (10) | 0.0 ± 0.00 (10) | 0.0 ± 0.00 (10) | 0.0 ± 0.00 (10) | 0.0 ± 0.00 (10) | 0.1 ± 0.10 (10) |  | 0.0 ± 0.00 (8) | 0.0 ± 0.00 (8) |
|  |  | F | 0.0 ± 0.00 (10) | 0.7 ± 0.21 (10)** | 0.0 ± 0.00 (10) | 0.2 ± 0.20 (10) | 0.2 ± 0.20 (10) | 0.2 ± 0.20 (10) |  | 0.0 ± 0.00 (8)* | 0.0 ± 0.00 (8) |
|  | Vacuolation (score) | M | 0.1 ± 0.10 (10) | 0.5 ± 0.17 (10) | 0.1 ± 0.10 (10) | 0.7 ± 0.21 (10)* | 0.9 ± 0.28 (10)* | 0.9 ± 0.18 (10)** |  | 0.0 ± 0.00 (8)* | 0.0 ± 0.00 (8)** |
|  |  | F | 0.0 ± 0.00 (10) | 0.0 ± 0.00 (10) | 0.0 ± 0.00 (10) | 0.0 ± 0.00 (10) | 0.0 ± 0.00 (10) | 0.0 ± 0.00 (10) |  | 0.0 ± 0.00 (8) | 0.0 ± 0.00 (8) |
| Gland, harderian | Any microscopic findings (incidence) | M | 0/10 | 0/10 | 0/10 | 0/10 | 0/10 | 0/10 |  | 0/8 | 0/8 |
|  |  | F | 0/10 | 0/10 | 0/10 | 0/10 | 0/10 | 0/10 |  | 0/8 | 3/8 |
|  | Infiltration (score) | M | 0.0 ± 0.00 (10) | 0.0 ± 0.00 (10) | 0.0 ± 0.00 (10) | 0.0 ± 0.00 (10) | 0.0 ± 0.00 (10) | 0.0 ± 0.00 (10) |  | 0.0 ± 0.00 (8) | 0.0 ± 0.00 (8) |
|  |  | F | 0.0 ± 0.00 (10) | 0.0 ± 0.00 (10) | 0.0 ± 0.00 (10) | 0.0 ± 0.00 (10) | 0.0 ± 0.00 (10) | 0.0 ± 0.00 (10) |  | 0.0 ± 0.00 (8) | 0.4 ± 0.18 (8)* |
| Gland, lacrimal | Any microscopic findings (incidence) | M | 0/10 | 1/10 | 2/10 | 2/10 | 0/10 | 0/10 |  | 3/8 | 1/8 |
|  |  | F | 0/10 | 0/10 | 0/10 | 0/10 | 0/10 | 0/10 |  | 0/8 | 1/8 |
|  | Infiltration (score) | M | 0.0 ± 0.00 (10) | 0.1 ± 0.10 (10) | 0.3 ± 0.21 (10) | 0.2 ± 0.13 (10) | 0.0 ± 0.00 (10) | 0.0 ± 0.00 (10) |  | 0.4 ± 0.18 (8) | 0.1 ± 0.13 (8) |
|  |  | F | 0.0 ± 0.00 (10) | 0.0 ± 0.00 (10) | 0.0 ± 0.00 (10) | 0.0 ± 0.00 (10) | 0.0 ± 0.00 (10) | 0.0 ± 0.00 (10) |  | 0.0 ± 0.00 (8) | 0.1 ± 0.13 (8) |
| Gland, mammary | Any microscopic findings (incidence) | M | 0/8 | 0/9 | 0/6 | 0/6 | 0/7 | 0/7 |  | 0/5 | 0/5 |
|  |  | F | 0/9 | 0/10 | 0/9 | 0/10 | 0/10 | 0/9 |  | 0/7 | 0/8 |
| Gland, parathyroid | Any microscopic findings (incidence) | M | 0/10 | 0/9 | 0/10 | 0/9 | 0/10 | 0/9 |  | 0/8 | 0/8 |
|  |  | F | 0/10 | 0/10 | 0/9 | 0/9 | 0/10 | 0/10 |  | 0/7 | 0/8 |
| Gland, pituitary | Any microscopic findings (incidence) | M | 0/10 | 0/10 | 0/10 | 0/10 | 1/10 | 0/10 |  | 0/8 | 0/8 |
|  |  | F | 0/10 | 0/10 | 0/10 | 0/10 | 0/10 | 0/10 |  | 0/8 | 1/8 |
|  | Cyst (score) | M | 0.0 ± 0.00 (10) | 0.0 ± 0.00 (10) | 0.0 ± 0.00 (10) | 0.0 ± 0.00 (10) | 0.2 ± 0.20 (10) | 0.0 ± 0.00 (10) |  | 0.0 ± 0.00 (8) | 0.0 ± 0.00 (8) |
|  |  | F | 0.0 ± 0.00 (10) | 0.0 ± 0.00 (10) | 0.0 ± 0.00 (10) | 0.0 ± 0.00 (10) | 0.0 ± 0.00 (10) | 0.0 ± 0.00 (10) |  | 0.0 ± 0.00 (8) | 0.1 ± 0.13 (8) |
| Gland, prostate | Any microscopic findings (incidence) | M | 0/10 | 0/10 | 1/10 | 0/10 | 2/10 | 0/10 |  | 0/8 | 0/8 |
|  | Infiltration (score) | M | 0.0 ± 0.00 (10) | 0.0 ± 0.00 (10) | 0.0 ± 0.00 (10) | 0.0 ± 0.00 (10) | 0.4 ± 0.27 (10) | 0.0 ± 0.00 (10) |  | 0.0 ± 0.00 (8) | 0.0 ± 0.00 (8) |
|  | Inflammation (score) | M | 0.0 ± 0.00 (10) | 0.0 ± 0.00 (10) | 0.3 ± 0.30 (10) | 0.0 ± 0.00 (10) | 0.0 ± 0.00 (10) | 0.0 ± 0.00 (10) |  | 0.0 ± 0.00 (8) | 0.0 ± 0.00 (8) |
| Gland, salivary | Any microscopic findings (incidence) | F | 0/10 | 0/10 | 0/10 | 0/10 | 0/10 | 0/10 |  | 0/8 | 0/8 |
|  |  | M | 0/10 | 0/10 | 0/10 | 0/10 | 0/10 | 0/10 |  | 0/8 | 0/8 |
| Gland, seminal | Any microscopic findings (incidence) | M | 0/10 | 0/10 | 0/10 | 0/10 | 0/10 | 1/10 |  | 0/8 | 0/8 |
|  | Infiltration (score) | M | 0.0 ± 0.00 (10) | 0.0 ± 0.00 (10) | 0.0 ± 0.00 (10) | 0.0 ± 0.00 (10) | 0.0 ± 0.00 (10) | 0.1 ± 0.10 (10) |  | 0.0 ± 0.00 (8) | 0.0 ± 0.00 (8) |
| Gland, thyroid | Any microscopic findings (incidence) | M | 0/10 | 0/10 | 0/10 | 0/10 | 0/10 | 0/10 |  | 0/8 | 0/8 |
|  |  | F | 0/10 | 0/10 | 0/9 | 0/10 | 0/10 | 0/10 |  | 0/8 | 0/8 |
| Heart | Any microscopic findings (incidence) | M | 0/10 | 0/10 | 0/10 | 0/10 | 1/10 | 1/10 |  | 1/8 | 0/8 |
|  |  | F | 1/10 | 0/10 | 0/10 | 0/10 | 1/10 | 0/10 |  | 0/8 | 0/8 |
|  | Infiltration (score) | M | 0.0 ± 0.00 (10) | 0.0 ± 0.00 (10) | 0.0 ± 0.00 (10) | 0.0 ± 0.00 (10) | 0.1 ± 0.10 (10) | 0.1 ± 0.10 (10) |  | 0.1 ± 0.13 (8) | 0.0 ± 0.00 (8) |
|  |  | F | 0.1 ± 0.10 (10) | 0.0 ± 0.00 (10) | 0.0 ± 0.00 (10) | 0.0 ± 0.00 (10) | 0.1 ± 0.10 (10) | 0.0 ± 0.00 (10) |  | 0.0 ± 0.00 (8) | 0.0 ± 0.00 (8) |
| Joint, stifle | Any microscopic findings (incidence) | M | 0/10 | 0/10 | 0/10 | 0/10 | 0/10 | 0/10 |  | 0/8 | 0/8 |
|  |  | F | 0/10 | 0/10 | 0/10 | 0/10 | 0/10 | 0/10 |  | 0/8 | 0/8 |
| Kidney | Any microscopic findings (incidence) | M | 4/10 | 1/10 | 1/10 | 1/10 | 3/10 | 2/10 |  | 0/8 | 3/8 |
|  |  | F | 6/10 | 0/10* | 0/10* | 1/10 | 3/10 | 2/10 |  | 3/8 | 1/8 |
|  | Infiltration (score) | M | 0.3 ± 0.15 (10) | 0.0 ± 0.00 (10) | 0.0 ± 0.00 (10) | 0.0 ± 0.00 (10) | 0.0 ± 0.00 (10) | 0.2 ± 0.13 (10) |  | 0.0 ± 0.00 (8) | 0.3 ± 0.16 (8) |
|  |  | F | 0.2 ± 0.13 (10) | 0.0 ± 0.00 (10) | 0.0 ± 0.00 (10) | 0.1 ± 0.10 (10) | 0.0 ± 0.00 (10) | 0.0 ± 0.00 (10) |  | 0.0 ± 0.00 (8) | 0.1 ± 0.13 (8) |
|  | Cyst (score) | M | 0.0 ± 0.00 (10) | 0.0 ± 0.00 (10) | 0.0 ± 0.00 (10) | 0.0 ± 0.00 (10) | 0.0 ± 0.00 (10) | 0.0 ± 0.00 (10) |  | 0.0 ± 0.00 (8) | 0.0 ± 0.00 (8) |
|  |  | F | 0.0 ± 0.00 (10) | 0.0 ± 0.00 (10) | 0.0 ± 0.00 (10) | 0.0 ± 0.00 (10) | 0.2 ± 0.13 (10) | 0.1 ± 0.10 (10) |  | 0.0 ± 0.00 (8) | 0.0 ± 0.00 (8) |
|  | Inflammation (score) | M | 0.0 ± 0.00 (10) | 0.0 ± 0.00 (10) | 0.0 ± 0.00 (10) | 0.0 ± 0.00 (10) | 0.2 ± 0.20 (10) | 0.0 ± 0.00 (10) |  | 0.0 ± 0.00 (8) | 0.3 ± 0.25 (8) |
|  |  | F | 0.0 ± 0.00 (10) | 0.0 ± 0.00 (10) | 0.0 ± 0.00 (10) | 0.0 ± 0.00 (10) | 0.3 ± 0.21 (10) | 0.0 ± 0.00 (10) |  | 0.0 ± 0.00 (8) | 0.0 ± 0.00 (8) |
|  | Basophilia (score) | M | 0.2 ± 0.13 (10) | 0.2 ± 0.20 (10) | 0.1 ± 0.10 (10) | 0.1 ± 0.10 (10) | 0.4 ± 0.22 (10) | 0.1 ± 0.10 (10) |  | 0.0 ± 0.00 (8) | 0.5 ± 0.33 (8) |
|  |  | F | 0.0 ± 0.00 (10) | 0.0 ± 0.00 (10) | 0.0 ± 0.00 (10) | 0.0 ± 0.00 (10) | 0.1 ± 0.10 (10) | 0.2 ± 0.13 (10) |  | 0.1 ± 0.13 (8) | 0.0 ± 0.00 (8) |
|  | Infarction (score) | M | 0.0 ± 0.00 (10) | 0.0 ± 0.00 (10) | 0.0 ± 0.00 (10) | 0.0 ± 0.00 (10) | 0.0 ± 0.00 (10) | 0.0 ± 0.00 (10) |  | 0.0 ± 0.00 (8) | 0.0 ± 0.00 (8) |
|  |  | F | 0.0 ± 0.00 (10) | 0.0 ± 0.00 (10) | 0.0 ± 0.00 (10) | 0.0 ± 0.00 (10) | 0.0 ± 0.00 (10) | 0.0 ± 0.00 (10) |  | 0.5 ± 0.33 (8) | 0.0 ± 0.00 (8) |
|  | Mineralization (score) | M | 0.0 ± 0.00 (10) | 0.0 ± 0.00 (10) | 0.0 ± 0.00 (10) | 0.0 ± 0.00 (10) | 0.0 ± 0.00 (10) | 0.0 ± 0.00 (10) |  | 0.0 ± 0.00 (8) | 0.0 ± 0.00 (8) |
|  |  | F | 0.4 ± 0.16 (10) | 0.0 ± 0.00 (10)* | 0.0 ± 0.00 (10)* | 0.0 ± 0.00 (10)* | 0.0 ± 0.00 (10)* | 0.0 ± 0.00 (10)* |  | 0.0 ± 0.00 (8) | 0.0 ± 0.00 (8) |
| Large intestine, cecum | Any microscopic findings (incidence) | M | 0/10 | 0/10 | 0/10 | 0/10 | 0/10 | 0/10 |  | 0/8 | 0/8 |
|  |  | F | 0/10 | 0/10 | 0/10 | 0/10 | 0/10 | 0/10 |  | 0/8 | 0/8 |
| Large intestine, colon | Any microscopic findings (incidence) | M | 0/10 | 0/10 | 0/10 | 0/10 | 0/10 | 0/10 |  | 0/8 | 0/8 |
|  |  | F | 0/10 | 0/10 | 0/10 | 0/10 | 0/10 | 0/10 |  | 0/8 | 0/8 |
| Large intestine, rectum/large intestine, anus | Any microscopic findings (incidence) | M | 0/10 | 0/10 | 0/10 | 0/10 | 0/10 | 0/10 |  | 0/8 | 0/8 |
|  |  | F | 0/10 | 0/10 | 0/10 | 0/10 | 0/10 | 0/10 |  | 0/8 | 0/8 |
| Liver | Any microscopic findings (incidence) | M | 5/10 | 5/10 | 1/10 | 3/10 | 2/10 | 9/10 |  | 1/8 | 4/8 |
|  |  | F | 4/10 | 5/10 | 1/10 | 1/10 | 1/10 | 3/10 |  | 3/8 | 4/8 |
|  | Infiltration (score) | M | 0.5 ± 0.17 (10) | 0.4 ± 0.16 (10) | 0.0 ± 0.00 (10)* | 0.1 ± 0.10 (10) | 0.1 ± 0.10 (10) | 0.1 ± 0.10 (10) |  | 0.0 ± 0.00 (8) | 0.1 ± 0.13 (8) |
|  |  | F | 0.4 ± 0.16 (10) | 0.4 ± 0.16 (10) | 0.1 ± 0.10 (10) | 0.1 ± 0.10 (10) | 0.2 ± 0.20 (10) | 0.2 ± 0.13 (10) |  | 0.4 ± 0.18 (8) | 0.4 ± 0.26 (8) |
|  | Hemorrhage (score) | M | 0.0 ± 0.00 (10) | 0.0 ± 0.00 (10) | 0.0 ± 0.00 (10) | 0.0 ± 0.00 (10) | 0.0 ± 0.00 (10) | 0.8 ± 0.33 (10)* |  | 0.0 ± 0.00 (8) | 0.0 ± 0.00 (8) |
|  |  | F | 0.0 ± 0.00 (10) | 0.0 ± 0.00 (10) | 0.0 ± 0.00 (10) | 0.0 ± 0.00 (10) | 0.0 ± 0.00 (10) | 0.0 ± 0.00 (10) |  | 0.0 ± 0.00 (8) | 0.0 ± 0.00 (8) |
|  | Vacuolation (score) | M | 0.0 ± 0.00 (10) | 0.0 ± 0.00 (10) | 0.0 ± 0.00 (10) | 0.1 ± 0.10 (10) | 0.0 ± 0.00 (10) | 0.2 ± 0.20 (10) |  | 0.0 ± 0.00 (8) | 0.0 ± 0.00 (8) |
|  |  | F | 0.0 ± 0.00 (10) | 0.0 ± 0.00 (10) | 0.0 ± 0.00 (10) | 0.0 ± 0.00 (10) | 0.0 ± 0.00 (10) | 0.0 ± 0.00 (10) |  | 0.0 ± 0.00 (8) | 0.0 ± 0.00 (8) |
|  | Degeneration (score) | M | 0.0 ± 0.00 (10) | 0.0 ± 0.00 (10) | 0.0 ± 0.00 (10) | 0.0 ± 0.00 (10) | 0.0 ± 0.00 (10) | 0.7 ± 0.37 (10) |  | 0.0 ± 0.00 (8) | 0.0 ± 0.00 (8) |
|  |  | F | 0.0 ± 0.00 (10) | 0.0 ± 0.00 (10) | 0.0 ± 0.00 (10) | 0.0 ± 0.00 (10) | 0.0 ± 0.00 (10) | 0.0 ± 0.00 (10) |  | 0.0 ± 0.00 (8) | 0.0 ± 0.00 (8) |
|  | Extramedullary Hematopoiesis (score) | M | 0.0 ± 0.00 (10) | 0.5 ± 0.17 (10)* | 0.1 ± 0.10 (10) | 0.2 ± 0.20 (10) | 0.2 ± 0.13 (10) | 0.5 ± 0.17 (10)* |  | 0.1 ± 0.13 (8) | 0.4 ± 0.18 (8) |
|  |  | F | 0.0 ± 0.00 (10) | 0.3 ± 0.15 (10) | 0.0 ± 0.00 (10) | 0.0 ± 0.00 (10) | 0.0 ± 0.00 (10) | 0.1 ± 0.10 (10) |  | 0.1 ± 0.13 (8) | 0.6 ± 0.32 (8) |
|  | Necrosis (score) | M | 0.0 ± 0.00 (10) | 0.1 ± 0.10 (10) | 0.0 ± 0.00 (10) | 0.0 ± 0.00 (10) | 0.0 ± 0.00 (10) | 0.2 ± 0.13 (10) |  | 0.0 ± 0.00 (8) | 0.0 ± 0.00 (8) |
|  |  | F | 0.0 ± 0.00 (10) | 0.1 ± 0.10 (10) | 0.0 ± 0.00 (10) | 0.0 ± 0.00 (10) | 0.0 ± 0.00 (10) | 0.3 ± 0.21 (10) |  | 0.0 ± 0.00 (8) | 0.4 ± 0.38 (8) |
| Lymph node, bronchial | Any microscopic findings (incidence) | M | 0/10 | 0/10 | 0/10 | 1/10 | 2/9 | 1/10 |  | 0/8 | 2/8 |
|  |  | F | 0/10 | 0/10 | 1/9 | 2/8 | 1/10 | 1/8 |  | 1/8 | 0/8 |
|  | Erythrocytosis/ Erythrophagocytosis (score) | M | 0.0 ± 0.00 (10) | 0.0 ± 0.00 (10) | 0.0 ± 0.00 (10) | 0.2 ± 0.20 (10) | 0.3 ± 0.24 (9) | 0.2 ± 0.20 (10) |  | 0.0 ± 0.00 (8) | 0.3 ± 0.16 (8) |
|  |  | F | 0.0 ± 0.00 (10) | 0.0 ± 0.00 (10) | 0.1 ± 0.11 (9) | 0.3 ± 0.16 (8) | 0.1 ± 0.10 (10) | 0.1 ± 0.13 (8) |  | 0.1 ± 0.13 (8) | 0.0 ± 0.00 (8) |
| Lymph node, mediastinal | Any microscopic findings (incidence) | M | 0/10 | 0/9 | 0/9 | 1/10 | 1/10 | 0/10 |  | 0/8 | 1/8 |
|  |  | F | 1/10 | 1/10 | 1/10 | 4/10 | 2/9 | 2/10 |  | 3/8 | 6/8 |
|  | Erythrocytosis/ Erythrophagocytosis (score) | M | 0.0 ± 0.00 (10) | 0.0 ± 0.00 (9) | 0.0 ± 0.00 (9) | 0.2 ± 0.20 (10) | 0.2 ± 0.20 (10) | 0.0 ± 0.00 (10) |  | 0.0 ± 0.00 (8) | 0.1 ± 0.13 (8) |
|  |  | F | 0.2 ± 0.20 (10) | 0.1 ± 0.10 (10) | 0.0 ± 0.00 (10) | 0.7 ± 0.33 (10) | 0.4 ± 0.29 (9) | 0.4 ± 0.27 (10) |  | 0.6 ± 0.38 (8) | 1.1 ± 0.30 (8) |
| Lymph node, mesenteric | Any microscopic findings (incidence) | M | 0/10 | 0/10 | 0/10 | 0/10 | 0/10 | 2/10 |  | 1/8 | 0/8 |
|  |  | F | 0/10 | 1/10 | 0/10 | 0/10 | 0/10 | 1/10 |  | 1/8 | 0/8 |
|  | Erythrocytosis/ Erythrophagocytosis (score) | M | 0.0 ± 0.00 (10) | 0.0 ± 0.00 (10) | 0.0 ± 0.00 (10) | 0.0 ± 0.00 (10) | 0.0 ± 0.00 (10) | 0.3 ± 0.21 (10) |  | 0.1 ± 0.13 (8) | 0.0 ± 0.00 (8) |
|  |  | F | 0.0 ± 0.00 (10) | 0.1 ± 0.10 (10) | 0.0 ± 0.00 (10) | 0.0 ± 0.00 (10) | 0.0 ± 0.00 (10) | 0.3 ± 0.30 (10) |  | 0.1 ± 0.13 (8) | 0.0 ± 0.00 (8) |
| Nerve, optic | Any microscopic findings (incidence) | M | 0/10 | 0/10 | 0/10 | 0/10 | 0/10 | 0/10 |  | 0/8 | 0/7 |
|  |  | F | 0/10 | 0/10 | 0/10 | 0/10 | 0/10 | 0/10 |  | 0/8 | 0/8 |
| Ovary | Any microscopic findings (incidence) | F | 0/10 | 0/10 | 0/10 | 0/10 | 0/10 | 0/10 |  | 0/8 | 0/8 |
| Pancreas | Any microscopic findings (incidence) | M | 0/10 | 1/10 | 1/10 | 0/10 | 0/10 | 0/10 |  | 1/8 | 0/8 |
|  |  | F | 0/10 | 0/10 | 0/10 | 0/10 | 0/10 | 0/10 |  | 0/8 | 0/8 |
|  | Infiltration (score) | M | 0.0 ± 0.00 (10) | 0.0 ± 0.00 (10) | 0.1 ± 0.10 (10) | 0.0 ± 0.00 (10) | 0.0 ± 0.00 (10) | 0.0 ± 0.00 (10) |  | 0.0 ± 0.00 (8) | 0.0 ± 0.00 (8) |
|  |  | F | 0.0 ± 0.00 (10) | 0.0 ± 0.00 (10) | 0.0 ± 0.00 (10) | 0.0 ± 0.00 (10) | 0.0 ± 0.00 (10) | 0.0 ± 0.00 (10) |  | 0.0 ± 0.00 (8) | 0.0 ± 0.00 (8) |
|  | Atrophy (score) | M | 0.0 ± 0.00 (10) | 0.1 ± 0.10 (10) | 0.0 ± 0.00 (10) | 0.0 ± 0.00 (10) | 0.0 ± 0.00 (10) | 0.0 ± 0.00 (10) |  | 0.1 ± 0.13 (8) | 0.0 ± 0.00 (8) |
|  |  | F | 0.0 ± 0.00 (10) | 0.0 ± 0.00 (10) | 0.0 ± 0.00 (10) | 0.0 ± 0.00 (10) | 0.0 ± 0.00 (10) | 0.0 ± 0.00 (10) |  | 0.0 ± 0.00 (8) | 0.0 ± 0.00 (8) |
| Sciatic nerve | Any microscopic findings (incidence) | M | 0/10 | 1/10 | 0/10 | 0/10 | 0/10 | 0/10 |  | 0/8 | 0/8 |
|  |  | F | 0/10 | 0/10 | 0/10 | 0/10 | 0/10 | 0/10 |  | 0/8 | 0/8 |
|  | Hemorrhage (score) | M | 0.0 ± 0.00 (10) | 0.1 ± 0.10 (10) | 0.0 ± 0.00 (10) | 0.0 ± 0.00 (10) | 0.0 ± 0.00 (10) | 0.0 ± 0.00 (10) |  | 0.0 ± 0.00 (8) | 0.0 ± 0.00 (8) |
|  |  | F | 0.0 ± 0.00 (10) | 0.0 ± 0.00 (10) | 0.0 ± 0.00 (10) | 0.0 ± 0.00 (10) | 0.0 ± 0.00 (10) | 0.0 ± 0.00 (10) |  | 0.0 ± 0.00 (8) | 0.0 ± 0.00 (8) |
| Skeletal, muscle | Any microscopic findings (incidence) | M | 0/10 | 0/10 | 0/10 | 0/10 | 0/10 | 0/10 |  | 0/8 | 0/8 |
|  |  | F | 0/10 | 0/10 | 0/10 | 0/10 | 0/10 | 1/10 |  | 0/8 | 0/8 |
|  | Degeneration (score) | M | 0.0 ± 0.00 (10) | 0.0 ± 0.00 (10) | 0.0 ± 0.00 (10) | 0.0 ± 0.00 (10) | 0.0 ± 0.00 (10) | 0.0 ± 0.00 (10) |  | 0.0 ± 0.00 (8) | 0.0 ± 0.00 (8) |
|  |  | F | 0.0 ± 0.00 (10) | 0.0 ± 0.00 (10) | 0.0 ± 0.00 (10) | 0.0 ± 0.00 (10) | 0.1 ± 0.10 (10) | 0.0 ± 0.00 (10) |  | 0.0 ± 0.00 (8) | 0.0 ± 0.00 (8) |
| Skin | Any microscopic findings (incidence) | M | 0/10 | 0/10 | 0/10 | 0/10 | 0/10 | 0/10 |  | 0/8 | 0/8 |
|  |  | F | 0/10 | 0/10 | 0/9 | 0/10 | 0/10 | 0/10 |  | 0/8 | 0/8 |
| Skin/subcutaneous | Any microscopic findings (incidence) | M |  | 2/2 | 1/1 | 3/3 | 3/3 | 1/1 |  |  |  |
|  |  | F | 1/1 | 3/10 | 2/9 | 7/10 | 7/10 | 7/10 |  | 2/8 | 2/8 |
|  | Inflammation (score) | M |  | 3.5 ± 0.50 (2) | 3.0 ± .(1) | 3.7 ± 0.33 (3) | 3.3 ± 0.33 (3) | 2.0 ± .(1) |  |  |  |
|  |  | F | 3.0 ± .(1) | 0.9 ± 0.46 (10) | 0.9 ± 0.59 (9) | 2.9 ± 0.64 (10) | 2.7 ± 0.60 (10) | 2.8 ± 0.61 (10) |  | 0.9 ± 0.58 (8) | 0.8 ± 0.49 (8)* |
|  | Ulceration (score) | M |  | 0.0 ± 0.00 (2) | 0.0 ± .(1) | 0.0 ± 0.00 (3) | 0.0 ± 0.00 (3) | 0.0 ± .(1) |  |  |  |
|  |  | F | 3.0 ± .(1) | 0.0 ± 0.00 (10)** | 0.0 ± 0.00 (9)** | 0.0 ± 0.00 (10)** | 0.0 ± 0.00 (10)** | 0.0 ± 0.00 (10)** |  | 0.0 ± 0.00 (8) | 0.0 ± 0.00 (8) |
| Small intestine, duodenum | Any microscopic findings (incidence) | M | 0/10 | 0/10 | 0/10 | 0/10 | 0/10 | 0/10 |  | 0/8 | 0/8 |
|  |  | F | 0/10 | 0/10 | 0/10 | 0/10 | 0/10 | 0/10 |  | 0/8 | 0/8 |
| Small intestine, ileum | Any microscopic findings (incidence) | M | 0/10 | 0/10 | 0/10 | 0/10 | 0/10 | 0/10 |  | 0/8 | 0/8 |
|  |  | F | 0/10 | 0/10 | 0/10 | 0/10 | 0/10 | 0/10 |  | 0/8 | 0/8 |
| Small intestine, jejunum | Any microscopic findings (incidence) | M | 0/10 | 0/10 | 0/10 | 0/10 | 0/10 | 0/10 |  | 0/8 | 0/8 |
|  |  | F | 0/10 | 0/10 | 0/10 | 0/10 | 0/10 | 0/10 |  | 0/8 | 0/8 |
| Spinal cord | Any microscopic findings (incidence) | M | 0/10 | 0/10 | 0/10 | 0/9 | 0/10 | 0/10 |  | 0/8 | 0/8 |
|  |  | F | 0/10 | 0/10 | 0/10 | 0/10 | 0/10 | 0/10 |  | 0/8 | 0/8 |
| Spleen | Any microscopic findings (incidence) | M | 0/10 | 0/10 | 0/10 | 0/10 | 0/10 | 0/10 |  | 0/8 | 0/8 |
|  |  | F | 1/10 | 0/10 | 0/10 | 0/10 | 0/10 | 0/10 |  | 0/8 | 0/8 |
|  | Depletion (score) | M | 0.0 ± 0.00 (10) | 0.0 ± 0.00 (10) | 0.0 ± 0.00 (10) | 0.0 ± 0.00 (10) | 0.0 ± 0.00 (10) | 0.0 ± 0.00 (10) |  | 0.0 ± 0.00 (8) | 0.0 ± 0.00 (8) |
|  |  | F | 0.1 ± 0.10 (10) | 0.0 ± 0.00 (10) | 0.0 ± 0.00 (10) | 0.0 ± 0.00 (10) | 0.0 ± 0.00 (10) | 0.0 ± 0.00 (10) |  | 0.0 ± 0.00 (8) | 0.0 ± 0.00 (8) |
| Stomach | Any microscopic findings (incidence) | M | 0/10 | 0/10 | 1/10 | 0/10 | 0/10 | 1/10 |  | 0/8 | 0/8 |
|  |  | F | 0/10 | 0/10 | 0/10 | 0/10 | 0/10 | 0/10 |  | 0/8 | 0/8 |
|  | Hemorrhage (score) | M | 0.0 ± 0.00 (10) | 0.0 ± 0.00 (10) | 0.0 ± 0.00 (10) | 0.0 ± 0.00 (10) | 0.1 ± 0.10 (10) | 0.0 ± 0.00 (10) |  | 0.0 ± 0.00 (8) | 0.0 ± 0.00 (8) |
|  |  | F | 0.0 ± 0.00 (10) | 0.0 ± 0.00 (10) | 0.0 ± 0.00 (10) | 0.0 ± 0.00 (10) | 0.0 ± 0.00 (10) | 0.0 ± 0.00 (10) |  | 0.0 ± 0.00 (8) | 0.0 ± 0.00 (8) |
|  | Abscess (score) | M | 0.0 ± 0.00 (10) | 0.1 ± 0.10 (10) | 0.0 ± 0.00 (10) | 0.0 ± 0.00 (10) | 0.0 ± 0.00 (10) | 0.0 ± 0.00 (10) |  | 0.0 ± 0.00 (8) | 0.0 ± 0.00 (8) |
|  |  | F | 0.0 ± 0.00 (10) | 0.0 ± 0.00 (10) | 0.0 ± 0.00 (10) | 0.0 ± 0.00 (10) | 0.0 ± 0.00 (10) | 0.0 ± 0.00 (10) |  | 0.0 ± 0.00 (8) | 0.0 ± 0.00 (8) |
| Testis | Any microscopic findings (incidence) | M | 6/10 | 3/10 | 7/10 | 3/10 | 2/10 | 2/10 |  | 1/8 | 1/8 |
|  | Degeneration (score) |  |  |  |  |  |  |  |  |  |  |
| Thymus | Any microscopic findings | M | 0/10 | 3/10 | 3/10 | 2/10 | 7/10** | 6/10* |  | 1/8 | 2/8 |
|  |  | F | 3/10 | 4/10 | 8/10 | 6/10 | 2/10 | 3/10 |  | 0/8 | 2/8 |
|  | Hemorrhage (score) | M | 0.0 ± 0.00 (10) | 0.4 ± 0.31 (10) | 0.4 ± 0.22 (10) | 0.3 ± 0.21 (10) | 0.4 ± 0.22 (10) | 0.4 ± 0.22 (10) |  | 0.0 ± 0.00 (8) | 0.1 ± 0.13 (8) |
|  |  | F | 0.0 ± 0.00 (10) | 0.6 ± 0.27 (10)* | 0.9 ± 0.23 (10)** | 0.7 ± 0.26 (10)* | 0.1 ± 0.10 (10) | 0.2 ± 0.20 (10) |  | 0.0 ± 0.00 (8) | 0.3 ± 0.16 (8) |
|  | Atrophy (soce) | M | 0.0 ± 0.00 (10) | 0.1 ± 0.10 (10) | 0.3 ± 0.15 (10) | 0.0 ± 0.00 (10) | 0.7 ± 0.15 (10)** | 0.6 ± 0.16 (10)** |  | 0.1 ± 0.13 (8) | 0.1 ± 0.13 (8) |
|  |  | F | 0.3 ± 0.15 (10) | 0.0 ± 0.00 (10) | 0.3 ± 0.15 (10) | 0.2 ± 0.13 (10) | 0.1 ± 0.10 (10) | 0.2 ± 0.13 (10) |  | 0.0 ± 0.00 (8) | 0.0 ± 0.00 (8) |
| Urinary bladder | Any microscopic findings (incidence) | M | 0/10 | 0/10 | 0/10 | 0/10 | 0/10 | 0/10 |  | 0/8 | 0/8 |
|  |  | F | 0/10 | 0/10 | 0/10 | 1/10 | 0/10 | 0/10 |  | 0/8 | 0/8 |
|  | Infiltration (score) | M | 0.0 ± 0.00 (10) | 0.0 ± 0.00 (10) | 0.0 ± 0.00 (10) | 0.0 ± 0.00 (10) | 0.0 ± 0.00 (10) | 0.0 ± 0.00 (10) |  | 0.0 ± 0.00 (8) | 0.0 ± 0.00 (8) |
|  |  | F | 0.0 ± 0.00 (10) | 0.0 ± 0.00 (10) | 0.0 ± 0.00 (10) | 0.1 ± 0.10 (10) | 0.0 ± 0.00 (10) | 0.0 ± 0.00 (10) |  | 0.0 ± 0.00 (8) | 0.0 ± 0.00 (8) |
| Uterus | Any microscopic findings (incidence) | F | 0/10 | 0/10 | 0/10 | 0/10 | 0/10 | 1/10 |  | 0/8 | 0/8 |
|  | Hemorrhage (score) | F | 0.0 ± 0.00 (10) | 0.0 ± 0.00 (10) | 0.0 ± 0.00 (10) | 0.0 ± 0.00 (10) | 0.0 ± 0.00 (10) | 0.2 ± 0.20(10) |  | 0.0 ± 0.00 (8) | 0.0 ± 0.00 (8) |

*Abbreviations: M, male; F, female.*

**Supplemental Table 15 Larynx morphometry results**

| **Organ** | **Parameter** | **Sex** | **Exposed** | | | | | |  | **Recovery** | |
| --- | --- | --- | --- | --- | --- | --- | --- | --- | --- | --- | --- |
|  |  |  | **PBS** | **PG/VG + Nic** | **PG/VG + F-Med** | **PG/VG+ Nic + F-Low** | **PG/VG + Nic + F-Med** | **PG/VG + Nic + F-High** |  | **PG/VG + Nic** | **PG/VG + Nic + F-High** |
| Larynx | Epithelial thickness at mid base of epiglottis (µm) | M | 14.3 ± 0.87 (10) | 15.1 ± 0.58 (9) | 13.5 ± 0.80 (9) | 17.3 ± 3.03 (10) | 15.8 ± 1.05 (10) | 14.4 ± 1.14 (9) |  | 21.1 ± 0.91 (8)*** | 17.6 ± 1.93 (8) |
|  |  | F | 13.4 ± 0.70 (10) | 13.3 ± 1.33 (10) | 13.1 ± 0.95 (10) | 16.6 ± 1.47 (10) | 13.4 ± 0.56 (10) | 16.2 ± 1.51 (10) |  | 14.7 ± 0.88 (8) | 16.9 ± 1.70 (7) |
|  | Epithelial thickness at ventrolateral floor of arytenoid (µm) | M | 6.6 ± 0.43 (10) | 9.4 ± 0.74 (9)** | 7.2 ± 0.65 (10) | 9.5 ± 1.25 (10) | 9.0 ± 1.03 (10) | 7.6 ± 0.79 (9) |  | 6.8 ± 0.75 (8)* | 6.7 ± 1.50 (8) |
|  |  | F | 5.7 ± 0.45 (10) | 8.8 ± 1.05 (10)* | 6.4 ± 0.42 (10) | 8.3 ± 0.72 (10)** | 6.4 ± 0.52 (9) | 7.7 ± 0.61 (10)* |  | 5.6 ± 0.41 (8)* | 6.6 ± 0.53 (8) |
|  | Epithelial thickness at arytenoid projections (µm) | M | 12.7 ± 0.72 (10) | 13.8 ± 0.63 (10) | 12.7 ± 0.48 (9) | 14.9 ± 1.30 (10) | 15.5 ± 1.24 (10) | 13.8 ± 0.63 (9) |  | 14.9 ± 1.60 (8) | 12.0 ± 0.82 (8) |
|  |  | F | 11.1 ± 0.62 (9) | 14.6 ± 1.34 (10)* | 11.7 ± 0.69 (10) | 15.1 ± 0.64 (10)*** | 14.3 ± 0.88 (9)** | 13.2 ± 0.77 (10) |  | 12.1 ± 0.76 (8) | 12.6 ± 0.56 (8) |

*Remarks:*

*Results represent mean ± SEM. The sample size is in parentheses.*

*The Exposed groups are compared against PBS during ‘Exposed’, while the Recovery groups are compared against the same treatment group from ‘Exposed’. Significance: *, p < 0.05; **, p < 0.01; ***, p < 0.001.*

*Abbreviations: M, male; F, female*

# References

Ackermann M, Strimmer K (2009) A general modular framework for gene set enrichment analysis BMC bioinformatics 10:47 doi:10.1186/1471-2105-10-47

Bolstad B, Bolstad MB, BiocGenerics I, biocViews Microarray O, Preprocessing Q (2013) Package ‘affyPLM’

Dai M et al. (2005) Evolving gene/transcript definitions significantly alter the interpretation of GeneChip data Nucleic acids research 33:e175-e175

Gentleman RC et al. (2004) Bioconductor: open software development for computational biology and bioinformatics Genome biology 5:R80

Liberzon A, Birger C, Thorvaldsdottir H, Ghandi M, Mesirov JP, Tamayo P (2015) The Molecular Signatures Database (MSigDB) hallmark gene set collection Cell systems 1:417-425 doi:10.1016/j.cels.2015.12.004

McCall MN, Bolstad BM, Irizarry RA (2010) Frozen robust multiarray analysis (fRMA) Biostatistics 11:242-253

Phillips B et al. (2015) Toxicity of aerosols of nicotine and pyruvic acid (separate and combined) in Sprague–Dawley rats in a 28-day OECD 412 inhalation study and assessment of systems toxicology Inhalation toxicology 27:405-431

Phillips B et al. (2017) Toxicity of the main electronic cigarette components, propylene glycol, glycerin, and nicotine, in Sprague-Dawley rats in a 90-day OECD inhalation study complemented by molecular endpoints Food and Chemical Toxicology 109:315-332

Smyth GK (2004) Linear models and empirical bayes methods for assessing differential expression in microarray experiments Statistical applications in genetics and molecular biology 3:Article3 doi:10.2202/1544-6115.1027

Wong ET et al. (2016) Evaluation of the Tobacco Heating System 2.2. Part 4: 90-day OECD 413 rat inhalation study with systems toxicology endpoints demonstrates reduced exposure effects compared with cigarette smoke Regulatory Toxicology and Pharmacology 81:S59-S81

Wu D, Lim E, Vaillant F, Asselin-Labat ML, Visvader JE, Smyth GK (2010) ROAST: rotation gene set tests for complex microarray experiments Bioinformatics (Oxford, England) 26:2176-2182 doi:10.1093/bioinformatics/btq401

Wu D, Smyth GK (2012) Camera: a competitive gene set test accounting for inter-gene correlation Nucleic Acids Res 40:e133 doi:10.1093/nar/gks461
