# Supplementary material for: Evaluation of toxicity of aerosols from flavored e-liquids in Sprague–Dawley rats in a 90-day OECD inhalation study, complemented by transcriptomics analysis
Source: Arch Toxicol. 2020 May 5;94(6):2179–206. doi: 10.1007/s00204-020-02759-6 (PMC7303093; doi:10.1007/s00204-020-02759-6)
Supplement: Supplementary file 8 — Supplementary file8 (PDF 281 kb) [file 204_2020_2759_MOESM8_ESM.pdf]

EU Chemical Group	Subgroup	Name	CAS Register Number	Cramer Class	TOPKAT Rat Inhalational LC50 (mg/m^3.h)	TOPKAT Ocular Irritancy (True=1; False=0)	TOPKAT Weight of Evidence Rodent Carcinogenicity Prediction (True=1; False=0)	TOPKAT Chronic LOAEL (mg/kg b.w.)	TOPKAT Developmental Toxicity Potential Prediction (True=1; False=0)	ECHA and others LD50 Acute Toxicity Oral (mg/kg)	Vapor Pressure (Torr)	Cramer Rules (Extension)	TOPKAT Rat Inhalational LC50 (mg/m^3.h)	TOPKAT Ocular Irritancy (True=1; False=0)	TOPKAT Weight of Evidence Rodent Carcinogenicity Prediction (True=1; False=0)	TOPKAT Chronic LOAEL (mg/kg b.w.)	TOPKAT Developmental Toxicity Potential Prediction (True=1; False=0)	ECHA and others LD50 Acute Toxicity Oral (mg/kg)	Vapor Pressure (Torr)	Total Score	Comments
1	1A	ETHYL FORMATE	109-94-4	Low (Class I)	12955.8369363087	true	true	91.317746135352	true	1850 mg/kg	242	0	2	1	1	2	1	2	2	11	
1	1A	ETHYL ACETATE	141-78-6	Low (Class I)	25572.7954912467	true	false	130.645883805029	false	10200 mg/kg ; 4100 mg/kg (ECHA Supp study)(mouse)	112	0	1	1	0	1	0	0	1	4	
1	1A	ETHYL 2-METHYLBUTYRATE	7452-79-1	Low (Class I)	61098.4106632592	true	false	134.332934227467	true	> 2000 mg/kg	7.85	0	0	1	0	0	1	1	0	3	
1	1A	ETHYL ISOBUTYRATE	97-62-1	Low (Class I)	65010.7279607403	true	true	118.281659626619	false	> 5000 mg/kg		0		1	1		0			2	
1	1A	AMYL FORMATE	638-49-3	Low (Class I)	35316.5471543871	true	false	530.318576307751	true	> 5000 mg/kg		0		1	0		1			2	
1	1A	BUTYL ISOVALERATE	109-19-3	Low (Class I)	150843.208440923	false	false	376.501521927849	true	5000 mg/kg		0		0	0		1			1	
1	1A	METHYL CAPROATE	106-70-7	Low (Class I)	46450.8454316349	false	false	395.772139205749	false	> 2000 mg/kg	3.95	0	0	0	0	0	0	1	0	1	
1	1A	ETHYL ISOVALERATE	108-64-5	Low (Class I)	59862.517587321	false	true	176.272371954212	false	> 5000 mg/kg		0		0	1		0			1	
1	1A	ETHYL BUTYRATE	105-54-4	Low (Class I)	43280.3235353232	false	false	315.353972307173	false	> 2000 mg/kg	13.9	0	0	0	0	0	0	1	0	1	
1	1A	ETHYL PROPIONATE	105-37-3	Low (Class I)	22641.4479547669	false	true	169.111437145403	false	8732 mg/kg		0		0	1		0			1	
1	1A	BUTYL BUTYRATE	109-21-7	Low (Class I)	55577.4174267657	false	false	494.709010457073	false	> 5000 mg/kg		0		0	0		0			0	
1	1A	OCTYL ISOBUTYRATE	109-15-9	Low (Class I)	114924.607439846	false	false	660.315746115602	false	>5000 mg/kg		0		0	0		0			0	
1	1A	AMYL CAPROATE	540-07-8	Low (Class I)	42338.6212234335	false	false	891.026179608917	false	>5000 mg/kg		0		0	0		0			0	
1	1A	AMYL BUTYRATE	540-18-1	Low (Class I)	51142.9897500926	false	false	719.858392284366	false	12210 mg/kg		0		0	0		0			0	
1	1A	BUTYL ACETATE	123-86-4	Low (Class I)	69895.2502054561	false	false	406.885118954188	false	12760 mg/kg		0		0	0		0			0	
1	1A	HEXYL ACETATE	142-92-7	Low (Class I)	61025.0885006901	false	false	825.642832625582	false	41500 mg/kg		0		0	0		0			0	
1	1A	ETHYL HEXANOATE	123-66-0	Low (Class I)	40995.0723564655	false	false	693.226025219689	false	> 5000 mg/kg		0		0	0		0			0	
1	1A	ETHYL HEPTANOATE	106-30-9	Low (Class I)	37720.4918120952	false	false	721.347592562023	false	5000 mg/kg		0		0	0		0			0	
1	1A	ETHYL NONANOATE	123-29-5	Low (Class I)	31229.8577531915	false	false	892.842346646324	false	>43000 mg/kg		0		0	0		0			0	
1	1B	BUTYRIC ACID	107-92-6	Low (Class I)	35752	true	false	126.730091135282	false	1632 mg/kg	1.35	0	0	1	0	2	0	2	2	7	
1	1B	DECANOIC ACID	334-48-5	Low (Class I)	26371	false	false	854.632876650764	false	> 2000 mg/kg	0.00355	0	2	0	0	0	0	1	0	3	
1	1B	HEXANOIC ACID	142-62-1	Low (Class I)	35958	true	false	579.245089409164	false	3000 mg/kg	0.158	0	0	1	0	1	0	0	1	3	
1	1B	NONANOIC ACID	112-05-0	Low (Class I)	28887	false	false	827.819817943701	false	> 2000 mg/kg	0.00867	0	1	0	0	0	0	1	0	2	
1	1B	OCTANOIC ACID	124-07-2	Low (Class I)	31395	false	false	795.547455773696	false	> 2000 mg/kg	0.022	0	0	0	0	0	0	1	0	1	
1	1B	LAURIC ACID	143-07-7	Low (Class I)	21565	false	false	893.731293440345	false	> 5000 mg/kg	0.000661	0	0	0	0	0	0	0	0	0	
1	1B	MYRISTIC ACID	544-63-8	Low (Class I)	17288	false	false	916.316409103496	false	10000 mg/kg	0.000139	0	0	0	0	0	0	0	0	0	
2		ISOBUTYL ALCOHOL	78-83-1	Low (Class I)	27832.957080813	true	true	114.655544166283	true	2830 mg/kg (ECHA)	16.4	0	1	1	1	2	1	1	2	9	
2		ISOVALERIC ACID	503-74-2	Low (Class I)	23472.0316374211	true	true	136.82068716917	true	2500 mg/kg	0.554	0	2	1	1	0	1	2	0	7	
2		METHYLBUTYRALDEHYDE, 3-	590-86-3	Low (Class I)	23020.8807746228	true	true	121.528634415055	true	5740 mg/kg		0		1	1		1			3	
2		2-METHYLBUTANAL	96-17-3	Low (Class I)	51198.1425883594	true	true	132.314608768892	true	6884 mg/kg		0		1	1		1			3	
2		3-METHYLBUTYL ISOBUTYRATE	2050-01-3	Low (Class I)	34922.946791677	true	true	144.435504631295	false	.	1.89	0	0	1	1	0	0	0	1	3	
2		ISOAMYL FORMATE	110-45-2	Low (Class I)	21984.5727009869	true	false	155.288483668826	true	9840 mg/kg(RTECS)		0		1	0		1			2	
2		3-METHYLPENTANOIC ACID	105-43-1	Low (Class I)	48778.9224909808	true	false	130.975623193404	false	.	0.147	0	0	1	0	1	0	0	0	2	
2		ISOAMYL ISOVALERATE	659-70-1	Low (Class I)	31236.0210428995	false	true	195.042331959228	false	> 5000 mg/kg		0		0	1		0			1	
2		ISOAMYL ACETATE	123-92-2	Low (Class I)	40895.3946827759	true	false	176.671583727873	false	7400 mg/kg		0		1	0		0			1	
2		ISOBUTYL ACETATE	110-19-0	Low (Class I)	36988.1394500405	false	false	138.33866165344	false	13413 mg/kg		0		0	0		0			0	
2		ISOAMYL BUTYRATE	106-27-4	Low (Class I)	139041.792078615	false	false	325.179965052773	false	5000 mg/kg (RTECS)		0		0	0		0			0	
3		ALLYL HEXANOATE	123-68-2	High (Class III)	49543.0566163758	false	true	272.044964735374	false	218 mg/kg		2	0	0	1	0	0	2		5	
3		GERANIOL	106-24-1	Low (Class I)	8543.05023472415	false	true	109.346569167854	true	3600 mg/kg		0	0	0	1	2	1	0		4	
3		2-DODECENAL, (2E)-	20407-84-5	Low (Class I)	25426.5133090489	false	false	577.525106518451	false	> 2000 mg/kg		0	2	0	0	0	0	1		3	
3		NEROL	106-25-2	Low (Class I)	8543.05000665609	false	true	109.346574967924	true	4500 mg/kg		0	0	0	1	1	1	0		3	
3		2,4-DECADIENAL, (E,E)-	25152-84-5	Low (Class I)	32297.0957367309	false	true	351.339324480803	false	.		0	1	0	1	0	0	0		2	
3		(E,Z)-2,6-NONADIENAL	557-48-2	Low (Class I)	16079.178502974	true	true	144.589351741841	false	>5000 mg/kg Moreno (1976b)		0		1	1		0			2	
3		CITRAL	5392-40-5	Low (Class I)	8814.57469784542	false	true	97.7090957137348	true	6800 mg/kg		0		0	1		1			2	
3		NERYL ACETATE	141-12-8	Low (Class I)	8550.25143757266	false	true	112.520567266649	false	>2000 mg/kg		0	0	0	1	0	0	1		2	
3		GERANYL TIGLATE	7785-33-3	Low (Class I)	7560.13617298946	false	true	91.822740070017	true	5000 mg/kg (RTECS)		0		0	1		1			2	
3		GERANYL BUTYRATE	106-29-6	Low (Class I)	30630.7875175214	false	true	136.664286695086	true	10660 mg/kg		0		0	1		1			2	
3		GERANYL FORMATE	105-86-2	Low (Class I)	6138.73068142279	false	true	137.871957407487	true	5532 mg/kg		0		0	1		1			2	
3		GERANYL ACETATE	105-87-3	Low (Class I)	8550.25109060035	false	true	112.520571672838	false	>4550 mg/kg		0	0	0	1	0	0	0		1	
4		CITRONELLOL, D-L-	106-22-9	Low (Class I)	21510.8293464264	true	true	115.382548805368	true	3450 mg/kg	1.83E-02	0	2	1	1	2	1	2	1	10	
4		CIS-3-HEXEN-1-YL ACETATE	3681-71-8	Low (Class I)	24865.1604784382	true	true	123.051178564545	false	> 2000 mg/kg	1.22	0	0	1	1	1	0	1	2	6	
4		CITRONELLYL ISOBUTYRATE	97-89-2	Low (Class I)	22843.2055722091	false	true	136.023614139491	true	> 2000 mg/kg	1.97E-02	0	1	0	1	0	1	1	0	4	
4		CITRONELLYL FORMATE	105-85-1	Low (Class I)	18266.1095124756	true	true	183.203853533884	true	> 6800 mg/kg		0		1	1		1			3	
4		RHODINOL	6812-78-8	Low (Class I)	31586.6627405337	false	true	341.181296906864	false	> mg/kg 5000		0		0	1		0			1	
4		CITRONELLYL BUTYRATE	141-16-2	Low (Class I)	90956.4697875259	false	false	154.51733129595	true	> 5000 mg/kg		0		0	0		1			1	
4		CITRONELLYL ACETATE	150-84-5	Low (Class I)	25421.8240580898	false	false	149.400123467478	false	6800 mg/kg		0		0	0		0			0	
5		HEPTANONE, 2-	110-43-0	Intermediate (Class II)	55996.2038786746	true	false	533.234861807137	false	1600 mg/kg	4.73E+00	1								1	
5		DIMETHYL-5,9-UNDECADIEN-2-ONE, 6,10-	3796-70-1	Low (Class I)	8061.33399666436	false	false	101.253240860991	true	5000 mg/kg	1.57E-02	0								0	
6		LINALOOL	78-70-6	Low (Class I)	15022.237205719	true	true	16.7731535146753	true	2200 mg/kg	9.05E-02	0	1	1	1	2	1	2	1	9	
6		TERPINEOL, ALPHA-	98-55-5	Low (Class I)	12191.5153973991	false	true	18.8460843573644	true	4300 mg/kg	3.92E-02	0	2	0	1	1	1	1	0	6	
6		2-METHYL-4-PHENYL-2-BUTANOL	103-05-9	Low (Class I)	6820.59974228985	true	false	116.226852195553	true	.	9.77E-02	0	0	1	0	0	1	0	2	4	
6		LINALYL BENZOATE	126-64-7	Intermediate (Class II)	5558.20637566992	false	true	83.4796995701753	true	5000 mg/kg		1		0	1		1			3	
6		LINALYL ISOBUTYRATE	78-35-3	Intermediate (Class II)	30469.6928378661	false	true	21.8377480600811	true	> 15100 mg/kg		1		0	1		1			3	
6		ISOPHYTOL	505-32-8	Low (Class I)	14533.0009832959	false	true	42.9272845771606	true	> 5400 mg/kg		0		0	1		1			2	
6		TERPINYL ACETATE, ALPHA-	80-26-2	Low (Class I)	10797.2860042479	false	true	25.9015165383416	true	5075 mg/kg		0		0	1		1			2	
8		MENTHONE	89-80-5	Intermediate (Class II)	37151.272627593	true	true	134.239381460448	false	1631-2347 mg/kg	0.256	1	0	1	1	0	0	2	2	7	
8		ALPHA-DAMASCONE	43052-87-5	Low (Class I)	7668.37342885506	true	true	10.464946561413	true		8.30E-03	0	1	1	1	2	1	0	0	6	
8		DAMASCENONE, BETA- (1-(2,6,6-TRIMETHYL-1,3-CYCLOHEXADIEN-1-YL)-2-BUTEN-1-ONE)	23696-85-7	Low (Class I)	6728.89953045203	true	true	11.706648712836	true	.	5.03E-03	0	0	1	1	1	1	0	0	4	
8		DAMASCONE, BETA- (1-(2,6,6-TRIMETHYL-1-CYCLOHEXEN-1-YL)-2-BUTEN-1-ONE), E	23726-91-2	Low (Class I)	7594.68161857695	false	true	26.932000692616	false	> 2000 mg/kg	6.55E-03	0	2	0	1	0	0	1	0	4	
8		PIPERITONE	89-81-6	Intermediate (Class II)	38845.5553258594	false	true	52.2035434473715	true	3550 mg/kg	5.72E-02	1	0	0	1	0	1	1	0	4	
8		CARVONE, L-	6485-40-1	Intermediate (Class II)	18287.4228462023	true	true	37.1884253872895	false	5400 mg/kg	6.56E-02	1	0	1	1	0	0	0	0	3	L-Carvone was included as aerosol marker.
8		MEGASTIGMATRIENONE (TABANON)	13215-88-8	Intermediate (Class II)	8704.4360175112	true	true	12.8742002377498	false	.	2.23E-03	1	0	1	1	0	0	0	0	3	
8		IRONE, ALPHA-	79-69-6	Low (Class I)	7976.58880540779	false	true	7.24271880300311	true	>5000 mg/kg		0		0	1	0	1		0	2	
8		MENTHYL ACETATE	29066-34-0 || 262	Low (Class I)	37794.738097699	true	false	83.1740246752778	false	.	7.07E-02	0	0	1	0	0	0	0	1	2	
8		IONONE, ALPHA-	127-41-3	Low (Class I)	8248.90520188754	false	true	12.5700446679012	false	4590 mg/kg	1.44E-02	0	0	0	1	0	0	0	0	1	
8		IONONE, BETA-	14901-07-6	Low (Class I)	8005.15552978416	false	true	32.5566823108265	false	4590 mg/kg	1.69E-02	0	0	0	1	0	0	0	0	1	
9		VALEROLACTONE, GAMMA-	108-29-2	Low (Class I)	15661.3120596248	false	true	53.8295392937011	false	> 5000 mg/kg	0.235	0	1	0	1	1	0		2	5	
9		HEXALACTONE, GAMMA-	695-06-7	Low (Class I)	38615.5415737465	false	false	52.151052449574	false	.	0.152	0	0	0	0	2	0		1	3	
9		OMEGA-PENTADECALACTONE	106-02-5	Low (Class I)	7117.40106091632	false	false	91.0309471123553	false	> 5000 mg/kg	6.43E-05	0	2	0	0	0	0		0	2	
9		HEPTALACTONE, GAMMA-	105-21-5	Low (Class I)	72877.7156814134	false	true	109.995589776296	false	.	8.27E-02	0	0	0	1	0	0		0	1	
9		OCTALACTONE, DELTA-	698-76-0	Low (Class I)	74330.1519265931	false	true	119.174249655449	false	> 5000 mg/kg	3.94E-02	0	0	0	1	0	0		0	1	
9		OCTALACTONE, GAMMA-	104-50-7	Low (Class I)	73562.2309255301	false	true	124.484226201575	false	.	4.08E-02	0	0	0	1	0	0		0	1	
9		WHISKEY LACTONE	39212-23-2	Low (Class I)	73311.8463335439	false	true	135.208200682512	false	.	2.70E-02	0	0	0	1	0	0		0	1	Lactones are all characterized with a LD50 ≥ 5000 mg/kg, the initial filter was not applied here.
9		DECALACTONE, DELTA-	705-86-2	Low (Class I)	67891.0286950143	false	false	166.250124938421	false	> 5000 mg/kg	8.25E-03	0	0	0	0	0	0		0	0	
9		DECALACTONE, GAMMA-	706-14-9	Low (Class I)	61932.9626728839	false	false	188.08712704556	false	> 5000 mg/kg	8.52E-03	0	0	0	0	0	0		0	0	
9		DELTA NONALACTONE	3301-94-8	Low (Class I)	74291.0581478307	false	false	114.638990941437	false	.	1.84E-02	0	0	0	0	0	0		0	0	
9		DODECALACTONE, DELTA-	713-95-1	Low (Class I)	55606.3655060178	false	false	174.149450844827	false	> 5000 mg/kg	1.55E-03	0	0	0	0	0	0		0	0	
9		DODECALACTONE, GAMMA-	2305-05-7	Low (Class I)	50726.3923592875	false	false	197.024031699815	false	.	1.59E-03	0	0	0	0	0	0		0	0	
9		NONALACTONE, GAMMA-	104-61-0	Low (Class I)	67771.3303162327	false	false	155.673409682126	false	> 5000 mg/kg	8.58E-03	0	0	0	0	0	0		0	0	
9		UNDECALACTONE, GAMMA-	104-67-6	Low (Class I)	56213.3800309416	false	false	193.063831451394	false	> 5000 mg/kg	2.71E-03	0	0	0	0	0	0		0	0	
10		3-METHYL-2,4-NONANEDIONE	113486-29-6	High (Class III)	95992.2933585944	true	false	609.359801778697	false	.				1	0		0			1	
12		ETHYL MALTOL	4940-11-8	Intermediate (Class II)	15213.79650931	true	false	9.387206085854	false	1220 mg/kg				1	0		0			1	
13		FURANEOL	3658-77-3	High (Class III)	9065.00466533064	true	false	19.0984328739199	true	>2320 mg/kg	0.032	2	2	1	0	0	1	2	1	9	
13		2-METHYLTETRAHYDROFURAN-3-ONE	3188-00-9	High (Class III)	14772.8801912216	true	false	46.3869993677948	true	.	6.56	2	0	1	0	0	1	1	2	7	
13		5-ETHYL-3-HYDROXY-4-METHYL-2(5H)-FURANONE	698-10-2	High (Class III)	22965.2741725309	true	false	10.7661715667658	true	.	3.47E-05	2	0	1	0	2	1	1	0	7	
13		4,5-DIMETHYL-3-HYDROXY-2,5-DIHYDROFURAN-2-ONE	28664-35-9	High (Class III)	9571.15678457096	true	false	17.1066320774191	true	.	4.81E-05	2	1	1	0	0	1	1	0	6	
13		5-ETHYL-4-HYDROXY-2-METHYL-3(2H)-FURANONE	27538-09-6	High (Class III)	19514.5023189497	true	false	11.3122073887759	false	.	1.21E-02	2	0	1	0	1	0	1	0	5	
15		PHENETHYL ALCOHOL	60-12-8	Low (Class I)	2862.46724184574	true	false	187.859955946827	true	1609 mg/kg	7.41E-02	0	1	1	0	0	1	2	1	6	
15		METHYL PHENYLACETATE	101-41-7	Low (Class I)	5972.98961026161	true	true	105.531380901807	false	2550 mg/kg	0.129	0	0	1	1	1	0	0	2	5	
15		PHENYLACETIC ACID	103-82-2	Low (Class I)	2343.50541586914	true	false	182.383975103952	false	2250 mg/kg	4.56E-03	0	2	1	0	0	0	1		4	
15		PHENETHYL CINNAMATE	103-53-7	Low (Class I)	2884.93519782088	false	false	66.2833258479253	true	4500 mg/kg	8.49E-07	0	0	0	0	2	1	0		3	
15		ISOAMYL PHENYLACETATE	102-19-2	High (Class III)|Intermediate (Class II)	15413.644693363	false	false	612.34602672254	false	> 5000 mg/kg	5.28E-03	2		0	0		0			2	
15		PHENETHYL ISOVALERATE	140-26-1	High (Class III)|Intermediate (Class II)	15413.644693363	false	false	639.764391743254	false	> 5000 mg/kg	3.47E-03	2		0	0		0			2	
15		ETHYL PHENYLACETATE	101-97-3	Low (Class I)	11337.7716676281	true	false	394.333955963931	false	3300 mg/kg	8.97E-02	0	0	1	0	0	0	0		1	
15		PHENETHYL PHENYLACETATE	102-20-5	Low (Class I)	3500.50265405244	false	false	272.109776088706	true	3190 mg/kg	2.30E-05	0	0	0	0	0	1	0		1	
15		PHENETHYL ACETATE	103-45-7	Low (Class I)	7744.7084156464	true	false	522.242730466617	false	>5000 mg/kg	5.64E-02	0		1	0		0			1	
15		PHENYLETHYL ESTER PROPANIOC AICD	122-70-3	Low (Class I)	13990.3338930398	false	false	731.549330896381	true	4000 mg/kg	1.99E-02	0	0	0	0	0	1	0		1	
16		EUCALYPTOL	470-82-6	High (Class III)	25053.8577345876	false	true	23.3247617555139	false	2480 mg/kg				0	1		0			1	
18		EUGENYL ACETATE	93-28-7	Low (Class I)	9554.72416656287	true	false	44.9148738151113	true	1670 mg/kg				1	0		1			2	
20		3-METHYLTHIOPROPIONALDEHYDE	3268-49-3	High (Class III)	3720.43255366247	true	false	112.369182463394	false	490 mg/kg	7.91E-03	2	2	1	0	1	0	2	0	8	
20		P-MENTHA-8-THIOL-3-ONE	38462-22-5	High (Class III)	7799.49469056957	true	false	101.115860251229	false	.	1.64E+00	2	1	1	0	2	0	0	1	7	
20		2-METHYL-4-PROPYL-1.3-OXATHIANE	67715-80-4	High (Class III)	89760.8389926421	true	false	198.655831823492	false	2500 mg/kg	5.87E+00	2	0	1	0	0	0	1	2	6	
20		METHYL THIOBUTYRATE	2432-51-1	High (Class III)	24875.4558079843	false	true	146.760080002549	true	.	7.91E-03	2	0	0	1	0	1	0	0	4	
21		BUTAN-2-ONE, 4-(PARA-HYDROXYPHENYL)-	5471-51-2	Low (Class I)	4866.57177587593	true	false	257.914458604562	true	> 2000 mg/kg	1.06E-03	0	2	1	0	0	1	2	0	6	Acetanisole and Butan-2-one, 4-(paraphydroxyphenyl) have the same score. Butan-2-one, 4-(parahydroxyphenyl) has been selected as FGR because of the higher predicted LC50.
21		ACETANISOLE	100-06-1	Low (Class I)	6000.07557217749	true	false	82.2208961682557	true	.	1.55E-02	0	1	1	0	2	1	0	1	6	
21		ALPHA-METHYLBENZYL ACETATE	93-92-5	Low (Class I)	14275.7595263598	false	false	255.951113445974	false	> 5000 mg/kg	2.03E-01	0	0	0	0	1	0	0	2	3	
22		CINNAMYL ALCOHOL	104-54-1	Low (Class I)	2480.99473622179	false	true	64.3225567555851	true	2675 mg/kg	1.17E-02	0	0	0	1	1	1	1	1	5	Cinnamyl alcohol and cinnamyl cinnamate have the same score and similar predicted LC50. Cinnamyl alcohol has been selectd as FGR because of higher volatility.
22		CINNAMYL CINNAMATE	122-69-0	Low (Class I)	2397.46938873957	false	true	47.2659668333059	true	4200 (Rat) Wohl (1974)	8.09E-08	0	1	0	1	2	1	0	0	5	
22		METHYL CINNAMATE	103-26-4	Low (Class I)	4450.99139660833	false	true	68.588409959274	true	2610 mg/kg	1.12E-02	0	0	0	1	0	1	2	0	4	
22		CINNAMIC ACID	621-82-9	Low (Class I)	1759.67248949688	false	true	144.251091500641	true	2500 mg/kg(RTECS); 3400 (Rat) Zaitsev and Rakhmanina (1974)	4.71E-03	0	2	0	1	0	1	0	0	4	
22		METHYLCINNAMALDEHYDE, ALPHA-	101-39-3	Low (Class I)	4770.63054946302	false	true	253.851687710877	true	2050 mg/kg	1.71E-02	0	0	0	1	0	1	0	2	4	
22		ISOAMYL CINNAMATE	7779-65-9	High (Class III)|Intermediate (Class II)	13233.3850479984	false	true	136.659325403542	true	.	5.05E-04	2	0	0	1	0	1	0	0	4	
22		ETHYL CINNAMATE	103-36-6	Low (Class I)	9870.34115777323	false	true	101.72418933381	true	4000 (Rat) Zaitsev and Rakhmanina (1974)	7.29E-03	0	0	0	1	0	1	0	0	2	
22		ALPHA-AMYLCINNAMALDEHYDE	122-40-7	Intermediate (Class II)	16230.2218689422	false	true	1020.63912890617	false	3730 mg/kg	2.33E-03	1	0	0	1	0	0	0	0	2	
22		3-PHENYLPROPYL ACETATE	122-72-5	Low (Class I)	7648.23468222289	true	false	932.761759137142	false	4700 mg/kg	1.06E-02	0	0	1	0	0	0	0	0	1	
22		ALPHA-HEXYLCINNAMIC ALDEHYDEALPHA-HEXYLCINNAMIC ALDEHYDE	101-86-0	Intermediate (Class II)	14555.1333570063	false	false	1089.02579069613	false	.	6.97E-04	1	0	0	0	0	0	0	0	1	
23		METHYL SALICYLATE	120-14-9	Low (Class I)	4055.19808380703	true	false	290.517489420476	true	887 mg/kg	7.00E-02	0	1	1	0		1	2		5	
23		ETHYL VANILLIN	121-32-4	Intermediate (Class II)	9061.00614886788	true	false	245.919562900366	true	3160 mg/kg	8.84E-04	1		1	0		1			3	
23		VERATRALDEHYDE	120-14-9	Low (Class I)	5908.08295634353	true	false	124.657715911407	true	2000 mg/kg	3.66E-03	0		1	0		1	1		3	
23		ANISYL PROPIONATE	7549-33-9	Low (Class I)	11001.1656052347	false	false	53.8421234284294	true	.	7.38E-03	0		0	0	2	1			3	
23		ANISE ALCOHOL	105-13-5	Low (Class I)	5243.92187459713	true	false	48.4185358042281	true	> 5000 mg/kg	6.79E-03	0		1	0		1			2	
23		BENZYL BENZOATE	120-51-4	Low (Class I)	3918.7627109557	false	false	164.253646127219	false	> 2000 mg/kg	2.50E-04	0	2	0	0		0			2	
23		BENZYL ACETATE	140-11-4	Low (Class I)	7227.4287632041	false	false	372.75175776146	false	> 2000 mg/kg	1.64E-01	0		0	0		0		2	2	
23		ANISYL BUTYRATE	6963-56-0	Low (Class I)	19825.2309092504	false	false	78.8598232808263	true	.	2.64E-03	0		0	0	1	1			2	
23		VANILLIN	121-33-5	Low (Class I)	4952.11880006871	true	false	169.35961357443	true	3978 mg/kg	1.94E-03	0		1	0		1			2	
23		4-HYDROXY-BENZENEMETHANOL	623-05-2	Low (Class I)	2096.11259131249	true	false	103.399933078779	true	.	1.04E-02	0		1	0		1			2	
23		BENZYL PROPIONATE	122-63-4	Low (Class I)	13151.8715875459	false	false	461.474842107257	false	> 2000 mg/kg	1.05E-01	0		0	0		0		1	1	
23		ISOAMYL BENZOATE	94-46-2	Low (Class I)	18444.1968878325	false	true	378.505641380451	false	6330 mg/kg	1.25E-02	0		0	1		0			1	
23		ANISYL ACETATE	104-21-2	Low (Class I)	6132.83100224292	false	false	44.1184097547114	true	>5000 mg/kg	2.05E-02	0		0	0		1			1	
23		BENZYL ISOBUTYRATE	103-28-6	Low (Class I)	16539.4295249031	false	false	341.639685498664	false	2850 mg/kg	5.13E-02	0		0	0		0			0	
23		BENZYL BUTYRATE	103-37-7	Low (Class I)	23992.9323631196	false	false	684.223368381777	false	2330 mg/kg	3.66E-02	0		0	0		0			0	
24		ETHYL-3,5-DIMETHYLPYRAZINE, 2-	13925-07-0	High (Class III)	59740.5968731215	true	false	56.9159591112723	false	500 (Moran et al) JECFA	0.814	2	0	1	0	2	0	2	0	7	
24		2-METHYLPYRAZINE	109-08-0	Intermediate (Class II)	14849.814652094	true	false	67.8965715793703	false	.	9.69	1	1	1	0	0	0	0	2	5	
24		ACETYLPYRAZINE	22047-25-2	High (Class III)	11350.0739313507	true	false	71.124499601512	false	>3000 (Rat)(Posternak et al)	0.169	2	2	1	0	0	0	0	0	5	
24		5-METHYL-6,7-DIHYDRO-5H-CYCLOPENTA[B]PYRAZINE	23747-48-0	High (Class III)	22381.9443934885	true	false	58.7358490265365	false	820 Wheldon et al. (1967) JECFA	0.291	2	0	1	0	1	0	0	0	4	
24		TETRAMETHYLPYRAZINE, 2,3,5,6-	1124-11-4	High (Class III)	26834.7814933976	true	true	58.9217719847117	false	1900(Rat)(Oser et al) JECFA	0.671	2	0	1	1	0	0	0	0	4	
24		ETHYL-3-METHYLPYRAZINE, 2-	15707-23-0	High (Class III)	38206.0032156172	true	false	69.4080183359102	false	600 mg/kg	1.69	2	0	1	0	0	0	1	0	4	
24		2-METHOXY-3(5 OR 6)-METHYLPYRAZINE (MIXTURE OF ISOMERS)	2847-30-5	High (Class III)	14597.944400879	true	false	88.8464807384837	false	.	3.27	2	0	1	0	0	0	0	1	4	
24		TRIMETHYLPYRAZINE, 2,3,5-	14667-55-1	High (Class III)	24437.8291599387	true	false	61.3056110344043	false	810(Rat)(Moran et al) JECFA	1.72	2	0	1	0	0	0	0	0	3	
24		ETHYL-3,6-DIMETHYLPYRAZINE, 2-	13360-65-1	High (Class III)	59740.5968731215	true	false	68.3154682258348	false	.	1.21	2	0	1	0	0	0	0	0	3	
24		2-ACETYL-3-ETHYLPYRAZINE	32974-92-8	High (Class III)	27663.8125341062	true	false	94.608740000888	false	.	2.58E-02	2	0	1	0	0	0	0	0	3	
24		2-METHOXY-3-(1-METHYLPROPYL)PYRAZINE	24168-70-5	High (Class III)	81401.5249136287	true	false	95.2943030046001	false	2000 (quest international)	9.95E-02	2	0	1	0	0	0	0	0	3	
24		2,3-DIETHYL-5-METHYL-PYRAZINE	18138-04-0	High (Class III)	56396.2777934278	true	false	109.974735615234	false	.	0.515	2	0	1	0	0	0	0	0	3	
24		2-ISOBUTYL-3-METHOXYPYRAZINE	24683-00-9	High (Class III)	36605.1619070845	true	false	135.454859130855	false	>4000 (Rat) Roure, Inc. (1974) 2000 Quest International (1983a) JECFA	0.273	2	0	1	0	0	0	0	0	3	
24		DIETHYLPYRAZINE, 2,3-	15707-24-1	High (Class III)	32705.8795739783	true	false	138.691806511356	false	.	1.03	2	0	1	0	0	0	0	0	3	
25		GUAIACOL	90-05-1	Low (Class I)	5655.00159983502	true	false	224.786755153682	true	725 mg/kg	0.179	0	2	1	0	0	1	2	2	8	
25		PROPENYLGUAETHOL	94-86-0	High (Class III)	13418.0699427917	true	false	119.304737469362	true	2400 mg/kg(RTECS)	2.81E-04	2	0	1	0	1	1	1	0	6	
25		2,3,6-TRIMETHYLPHENOL	2416-94-6	High (Class III)	8070.61756432882	true	false	92.9242376307008	false	3600 mg/kg	6.61E-02	2	1	1	0	2	0	0	0	6	
25		2-METHOXY-4-METHYLPHENOL	93-51-6	Low (Class I)	10951.909403295	true	false	208.282346697151	true	2600 mg/kg(RTECS)	7.83E-02	0	0	1	0	0	1	0	0	2	
25		4-PROPYLPHENOL	645-56-7	Low (Class I)	29763.0373012517	true	false	179.752314377361	true	.	3.85E-02	0	0	1	0	0	1	0	0	2	
25		4-ETHYL-2-METHOXY-PHENOL	2785-89-9	Low (Class I)	22497.3030045829	true	false	222.443882560946	true	.	1.73E-02	0	0	1	0	0	1	0	0	2	
25		2-METHOXY-4-PROPYL-PHENOL	2785-87-7	Low (Class I)	41300.6930956401	true	false	372.039498221135	true	2600 mg/kg	6.24E-03	0	0	1	0	0	1	0	0	2	
25		2-ISOPROPYLPHENOL	88-69-7	Low (Class I)	16773.2079267484	true	false	451.393705174516	false	.	0.112	0	0	1	0	0	0	0	1	2	
27		METHYL ANTHRANILATE	134-20-3	High (Class III)	2141.92659125771	false	true	106.796320475053	false	2910 mg/kg				0	1		0			1	
28		2-ACETYLPYRIDINE	1122-62-9	High (Class III)	9949.23262082338	true	false	107.880846970861	false	2280 mg/kg (RTECS) Posternak et al. (1975)	0.481	2	2	1	0	1	0	2	1	9	Very similar molecules. 2-Acetylpyridine has been selected as representative.
28		3-ETHYLPYRIDINE	536-78-7	High (Class III)	28419.4690182312	true	false	87.1981282860642	false	.	2.42	2	1	1	0	2	0	1	2	9	
29		2-ACETYLTHIAZOLE	24295-03-2	High (Class III)	9308.07769923692	true	false	65.4323472564146	false	.				1	0		0			1	
30		METHYL CYCLOPENTENOLONE (CYCLOTENE)	80-71-7	High (Class III)	9264.41444148709	true	true	81.7912715910939	true	>2000 mg/kg	4.84E-03	2	2	1	1	1	1	2	1	11	
30		METHYLCYCLOHEXANE-1,2-DIONE, 3- (COFFEE DIONE)	3008-43-3	Intermediate (Class II)	10965.972456994	true	true	215.24287400764	true	2700(PAFA)	0.321	1	1	1	1	0	1	1	2	8	
30		HEDIONE (METHYL DIHYDROJASMONATE)	24851-98-7	High (Class III)	31701.6602038796	false	false	115.469044368897	true	>5000 mg/kg	7.10E-04	2		0	0		1		0	3	
30		TRIETHYL CITRATE	77-93-0	High (Class III)	3505.57525373527	false	false	80.6904651808743	false	4000 mg/kg	1.75E-04	2	0	0	0	2	0	0	0	4	
31		ALPHA-PINENE	80-56-8	Low (Class I)	16647.0144757202	true	true	5.56045372351247	true	500 mg/kg	3.49	0	2	1	1	2	1	2	2	11	
31		PHELLANDRENE, ALPHA-	99-83-2	Low (Class I)	46511.5293265118	true	true	13.6390232507799	false	3740 mg/kg bw. (ECHA key study)	1.86	0	0	1	1	1	0	1	0	4	
31		CIS-OCIMENE	13877-91-3	Low (Class I)	23023.9632837629	true	true	24.9379545940219	true	5000 mg/kg	1.56	0	0	1	1	0	1	0	0	3	
31		PINENE, BETA-	127-91-3	Low (Class I)	20288.7199414204	false	true	30.6934393574685	true	> 5 000 mg/kg	2.4	0	0	0	1	0	1	0	1	3	
31		TERPINOLENE	586-62-9	Low (Class I)	18177.1692063614	false	true	67.5785521161654	false	3740 mg/kg	1.13	0	1	0	1	0	0	1	0	3	
31		CARYOPHYLLENE, BETA-	87-44-5	Low (Class I)	19918.8359702256	false	true	14.6385094946671	true	> 5000 mg/kg	1.28E-02	0	0	0	1	0	1	0	0	2	
31		LIMONENE, D-	5989-27-5	Low (Class I)	23211.2376456005	false	false	66.3159973779931	false	> 2000 mg/kg	1.54	0	0	0	0	0	0	0	0	0	
